# An Ocean of Opsins

**DOI:** 10.1093/gbe/evaf189

**Published:** 2025-11-04

**Authors:** Giacinto De Vivo, Eric Pelletier, Roberto Feuda, Salvatore D’Aniello

**Affiliations:** Department of Biology and Evolution of Marine Organisms (BEOM), Stazione Zoologica Anton Dohrn, Napoli, Italy; Metabolic Genomics, Genoscope, Institut de Biologie François-Jacob, CEA, CNRS, Université Paris Saclay, Evry, France; Research Federation for Global Ocean System Ecology & Evolution, CNRS, Paris, France; Department of Biology and Evolution of Marine Organisms (BEOM), Stazione Zoologica Anton Dohrn, Napoli, Italy; Neurogenetics Group, Division of Genetics and Genome Biology, School of Biology and Biomedical Sciences, College of Life Sciences, University of Leicester, Leicester LE1 7RH, UK; Department of Biology, Geology and Environmental Science, University of Bologna, Bologna, Italy; Department of Biology and Evolution of Marine Organisms (BEOM), Stazione Zoologica Anton Dohrn, Napoli, Italy

**Keywords:** phylogenetics, metatranscriptomics, metagenomics, photoreception, plankton, marine biodiversity

## Abstract

In this study, we explored the diversity and evolution of opsins using meta-omic data from the Tara Oceans and Tara Polar Circle expeditions, one of the largest marine datasets available. By using sequence similarity methods and phylogenetic analyses, we identified opsins across the different metazoan groups. Our results indicate that most of the opsin sequences belong to arthropods and vertebrates. We also detected sequences from all known opsin subfamilies, including r-opsin, c-opsin, xenopsin, and Group-4 opsins. Despite the broad taxonomic scope, no new opsin families were discovered; however, we provide valuable taxonomic insights into known opsin subfamilies and reinforce existing phylogenetic hypotheses. Additionally, we present novel opsin sequences from less-studied taxa, such as chaetognaths, rotifers, acoelomates, and tunicates, and which may serve as a valuable resource for future research into opsin function and diversity.

Significance In this study, we performed a large-scale survey of metagenomic and metatranscriptomic datasets from the Tara Oceans and Tara Polar Circle expeditions to identify opsins, the key genes underlying animal vision. Our results reveal the global distribution of opsin families across major marine zooplankton lineages, including several understudied taxa, and refine current models of opsin evolution. This work provides one of the most comprehensive resources to date for investigating the molecular basis and evolutionary history of photoreception in complex marine metazoan communities.

## Introduction

Plankton forms the foundation of the marine food web and harbors a vast portion of the biodiversity of the oceans. To better understand this diversity, the Tara Oceans and Tara Polar Circle expeditions embarked on a global journey, collecting and characterizing plankton from ecological, morphological, and molecular perspectives (Karsenti et al. 2011; Carradec et al. 2018; Salazar et al. 2019; Sunagawa et al. 2020). With over 210 sampling locations worldwide and depths reaching up to 1,000 meters, Tara expeditions have generated one of the most taxonomically rich genomic and transcriptomic datasets available. This remarkable resource provides an unprecedented opportunity to explore the evolution of gene families across a wide array of marine zooplankton.

Despite extensive research into zooplankton diversity, much of the marine biome remains unexplored at the molecular level, especially in terms of gene families crucial for ecological adaptations. As key proteins required for animal vision and photoreception in general (Terakita 2005; Porter et al. 2012), opsins play an especially important role in shaping species interactions and ecology. Traditionally, animal opsins have been categorized in three main paralogue groups: ciliary opsin (c-opsin), rhabdomeric opsin (r-opsin), and Group-4 opsins (Feuda et al. 2012; Fleming et al. 2020). Group-4 is composed of Go-opsins, neuropsin, Retinal G protein-coupled receptor (RGR), peropsin, and retinochrome. However, recent studies have challenged this view, suggesting the existence of additional paralog groups. Indeed, new subfamilies such as xenopsin, non-canonical r-opsin, bathyopsin, chaopsin, anthozoan-specific opsins (ASO-I and ASO-II), and pseudospin have been proposed (D’Aniello et al. 2015; Ramirez et al. 2016; Gornik et al. 2020; De Vivo et al. 2023). The relationships between these subfamilies are still subject to debate, with some phylogenies suggesting the monophyly of Group-4 opsins and c-opsins with r-opsins sister group to them (Davies et al. 2010; Feuda et al. 2012, 2014; Yoshida et al. 2015; Vöcking et al. 2017; Rawlinson et al. 2019; Bonadè et al. 2020; Fleming et al. 2020; McCulloch et al. 2023), while others support the monophyly of Group-4 and r-opsins (Porter et al. 2012). In contrast, xenopsins are recovered in a variety of positions, sometimes closer to c-opsins (Yoshida et al. 2015; Vöcking et al. 2017, 2021; McCulloch et al. 2023) or closer to Group-4 opsins (Ramirez et al. 2016; Rawlinson et al. 2019; Bonadè et al. 2020). Depending on the organism, an opsin can have a different role, for example, c-opsins are involved in image-forming vision in vertebrates, while most invertebrates (e.g. cephalopods and arthropods) use r-opsins (Terakita 2005). Similarly, depending on the organism, there are some opsins that might serve as photoisomerases, able to restore the original configuration of the chromophore; this is the case of retinochromes in cephalopods and RGRs in vertebrates. Furthermore, recently, studies have shown that opsins are not only involved in photoreception but also in other mechanisms, such as mechanotransduction and temperature sensitivity (Leung and Montell 2017; Feuda et al. 2022; Chandel et al. 2024).

A key limitation in understanding opsin diversity and evolution stems from a focus on well-characterized species, whose genomes or transcriptomes have been sequenced. This narrow taxonomic scope may obscure the full range of opsin diversity and hamper a comprehensive understanding of their evolutionary history. To address these gaps, this study investigates opsin diversity within the metatranscriptomic (MATOU) and metagenomic (SMAGs) datasets of the Tara Oceans and Tara Arctic expeditions—the largest -omics dataset of marine plankton (Karsenti et al. 2011; Carradec et al. 2018; Salazar et al. 2019; Sunagawa et al. 2020). This dataset offers an unprecedented chance to examine opsin diversity across a taxonomically and ecologically diverse range of marine organisms, providing new insights into the evolution and function of this critical gene family. Our results provide a quantitative description of animal opsin diversity within the Tara datasets, examining their taxonomic distribution and subfamily composition and addressing their phylogenetic relationships. By doing that, we aim to provide a framework for future research on metazoan vision and contribute to a better understanding of opsin evolution across marine biomes.

## Results

We analyzed over 158 million eukaryotic gene sequences obtained from plankton samples collected at more than 210 stations globally. Using a combination of BLAST, clustering, and annotation methods (Fig. S1), we identified 2091 opsin sequences (see Methods). To ensure robust taxonomic classification and phylogenetic resolution, we integrated these with 738 well-annotated reference opsins from across metazoan diversity (2,829 sequences in total; File S1). This dataset (Dataset 1) was used to reconstruct the phylogenetic relationships using maximum likelihood under the GTR20+G model in IQ-TREE2 (Minh et al. 2020), followed by RogueNaRok (Aberer et al. 2013) to remove unstable sequences (Fig. S2).

**Fig. 1.**
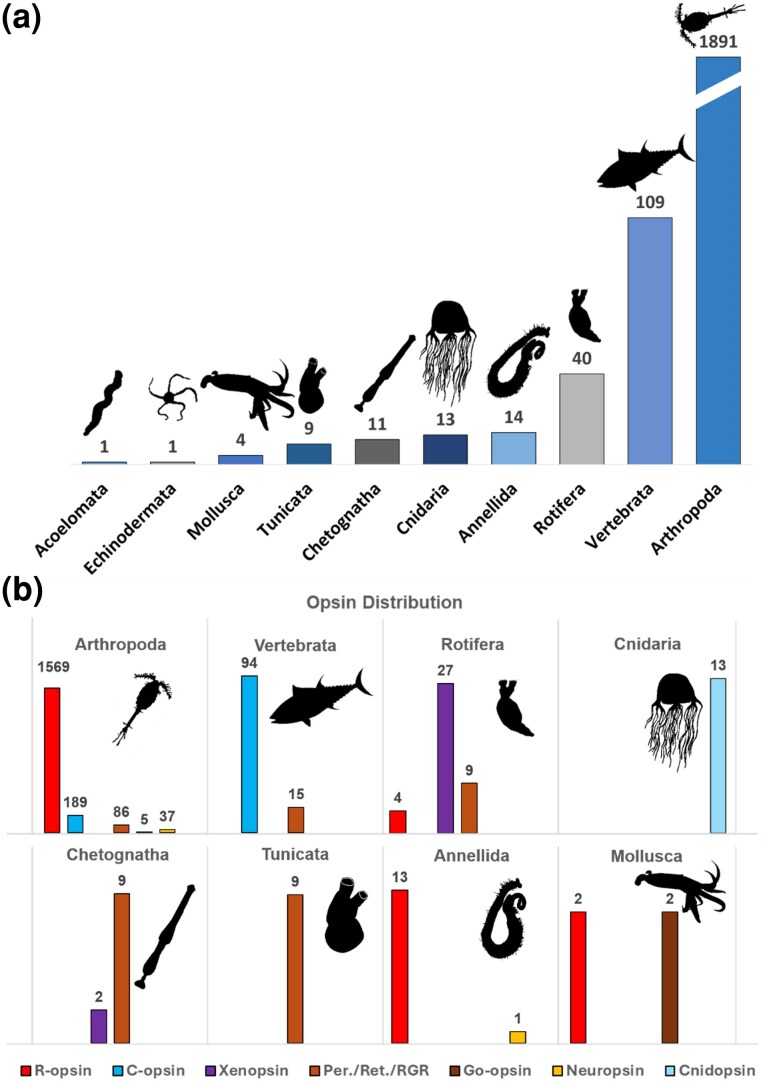
Opsin taxonomic distribution mined in Tara. a) A plot showing the estimated number of opsin genes in Tara per taxonomical group. b) Distribution of opsin subfamilies within different taxonomical groups. Numbers indicate the assembled opsin sequences found. Animal silhouettes from Phylopic.

Our results indicate that the vast majority of opsin (1,891 sequences, ∼80%) were assigned to arthropods, which dominate zooplankton communities. However, we identified sequences from other phyla such as cnidarians, acoelomates, mollusks, annelids, chaetognaths, rotifers, echinoderms, and vertebrates (Fig. 1a). The sequence diversity spanned across all r-opsin, c-opsin, xenopsin, and Group-4 clades (Fig. 1b). In particular, 1,569 arthropod opsins are r-opsins, 189 c-opsins, 86 peropsins, 5 Go-opsins, and 37 neuropsins. Vertebrates, the second most represented group with a total of 109 opsin sequences, comprise 94 c-opsins and 15 peropsins. Rotifers were the third most abundant group, with 40 sequences in total: 4 r-opsins, 27 xenopsins, and 9 peropsins. Annelids, the fourth most represented group, have 13 r-opsins and 1 neuropsin. Cnidarians have 13 sequences, all of which clustered with a group containing annotated cnidarian sequences referred to as c-opsin in Fleming et al. (2020) and as cnidopsins in McCulloch et al. (2023). Among mollusks, we identified four sequences: two r-opsins and two Go-opsins. In chaetognaths, we found two xenopsins and nine peropsins. Despite the presence of eyes in chaetognaths, this observation is consistent with previous studies, which only found one xenopsin and one peropsin, highlighting the absence of canonical visual opsins (e.g. r- and c-opsin) (Wollesen et al. 2023). Additionally, nine tunicate sequences form a monophyletic group belonging to the retinochromes/RGR. The single opsin found in acoelomates belongs to the r-opsin clade. The only echinoderm sequence in our dataset forms a monophyletic group with a clade of early divergent cnidarian opsin.

**Fig. 2.**
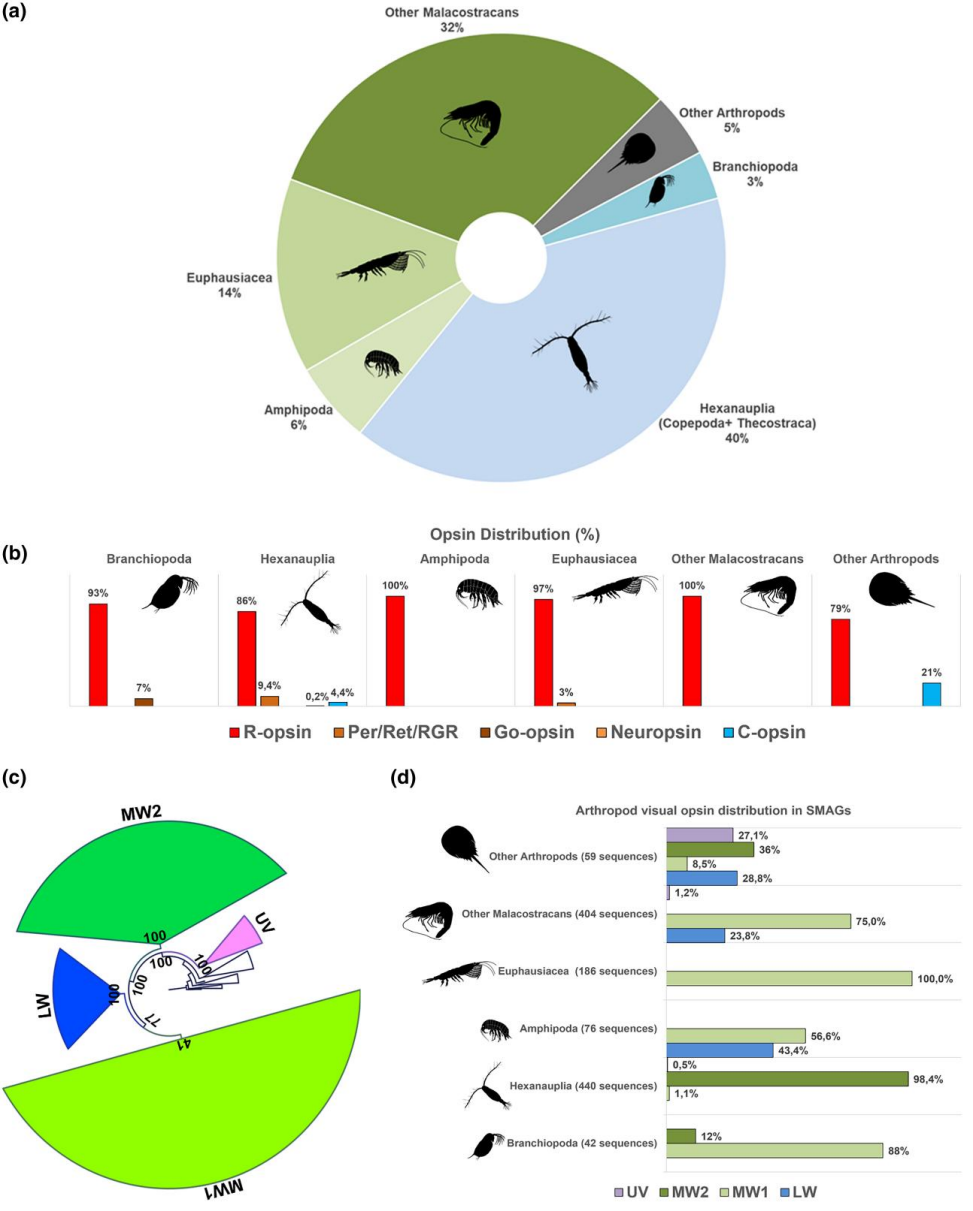
Opsin taxonomic distribution in arthropods using SMAGs annotations. a) A plot showing the estimated number of opsin genes in Tara per taxonomical group. b) Distribution of opsin subfamilies in TARA within different taxonomical groups. c) Phylogenetic relationship of arthropods’ r-opsin in TARA and their distribution within the different taxa d). Numbers indicate the percentage of assembled opsin sequences. Color code in a): Malacostracans in green, Hexanauplia in light blue, Brachiopoda in sapphire, and other Arthropods in gray. In d), outgroups are represented by r-opsin sequences from other taxa; UFB values are indicated at nodes. Animal silhouettes from Phylopic.

Given the prevalence of arthropods in our dataset, we next focused our analysis on this group. Using SMAG annotations, we identified sequences from Malacostraca (e.g. Euphausiacea, such as krill, and Amphipoda), Hexanauplia (e.g. copepods), and Branchiopoda (Fig. 2a and b). To better understand their phylogenetic relationships, we performed a phylogenic analysis combining these sequences with the visual opsins from Fleming et al. (2021). This dataset was analyzed using maximum likelihood under the GTR20+G model as implemented in IQ-TREE2, and then, RogueNaRok was used to remove unstable sequences (Dataset 2, File S2). Our results (Fig. 2c; Fig. S3) suggest the vast majority of arthropod opsins from TARA belong to MW2 (medium wavelength opsin 2) from copepods and MW1 (medium wavelength opsin 1) from malacostracans (Fig. 2d).

**Fig. 3.**
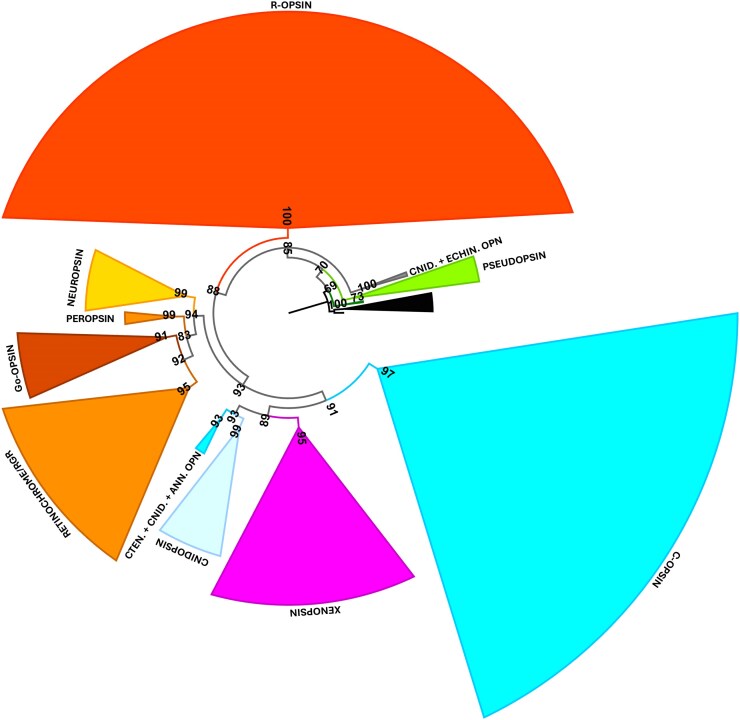
Maximum likelihood phylogenetic tree (GTR20+G) showing the relationships between the main opsin subfamilies: r-opsin (red), Group-4 opsin (orange, yellow, brown), xenopsin (magenta), ctenopsin and c-opsin (cyan), and cnidopsin (light cyan). Divergent cnidarian opsins are represented in gray, while the outgroups placopsin and pseudopsin are in dark and light green, respectively. In this tree, 95% of arthropod sequences have been removed. UFB values are indicated at nodes.

To investigate the relationships between Tara opsins, we generated a third dataset, by removing 95% of arthropod sequences (Fig. 3). This dataset composed of 298 Tara sequences + 738 reference sequences (File S3) was subject to a phylogenetic analysis using maximum likelihood under the GTR20+G model as implemented in IQ-TREE2 and then RogueNaRok to remove unstable sequenced. The phylogenetic tree (Fig. 3; Fig. S4) supports the existence of the four monophyletic groups: r-opsin (UFB = 100), c-opsin (UFB = 97), xenopsin (UFB = 95), and Group-4 opsin (UFB = 94). Group-4 includes peropsin (UFB = 99), retinochrome/RGR (UFB = 95), Go-opsins (UFB = 91), and neuropsins (UFB = 99). The earliest emerging opsin group (UFB = 100) contains sequences from cnidarians and one sequence from echinoderm. This clade includes sequences classified in chaopsin in Ramirez et al. (2016), cnidarian r-opsins in Fleming et al. (2020) and as ASO-I in Gornik et al. (2020). Furthermore, we identified two groups: the first includes cnidopsins (UFB = 99) and the second comprises ctenophore opsins, often referred to as ctenopsins (e.g. McCulloch et al. 2023), which form a monophyletic clade together with c-opsins from cnidarians and annelids (UFB = 93). This clade is recovered as sister to xenopsins (UFB = 89), and together, they form the sister group to the canonical c-opsins (UFB = 97). Furthermore, our phylogeny supports Go-opsins, and not peropsin, as sister group of RGR/retinochromes (UFB = 92). Notably, this RGR/retinochrome clade also includes sequences (e.g. those from annelids and chaetognaths) that have been classified as peropsins in other studies (De Vivo et al. 2023; Wollesen et al. 2023). Finally, our phylogenetic analysis helps to clarify the relationships between r-opsins, xenopsins, c-opsins, and Group-4, suggesting r-opsins as the first diverging group (UFB = 88) and Group-4 in sister relationship to the xenopsin/c-opsin clade (UFB = 93).

## Discussion

Our results provide new insights into the diversity and evolution of metazoan opsins. Mining 158 million unbiased eukaryotic gene sequences from plankton revealed no compelling evidence for additional, previously unknown opsin subfamilies. Rather, all opsins recovered from the TARA dataset fell within well-supported monophyletic groups corresponding to known opsin paralogs.

However, our study also highlights that, despite its scale, this dataset may underrepresent certain opsin subfamilies. For instance, excluding the highly expressed visual opsins (c- and r-opsins), subfamilies such as peropsins and xenopsins are notably underrepresented. This could be linked to the extensive gene duplications observed in opsins involved in visual tasks, such as those that mediate color vision. For instance, 77 out of 106 vertebrate c-opsins identified in this study belong to green-sensitive opsins of the rhodopsin-like 2 (Rh2) group, which have undergone substantial duplications in fishes (Hagen et al. 2023).

From the phylogenetic point of view, our results suggest that ctenopsins, cnidopsins, and xenopsins might represent an extended c-opsin group spanning within all the different phyla, respectively, the c-opsin of ctenophores, of cnidarian, and of spiralia. Overall, our results are consistent with previous topologies, placing r-opsins as an early-diverging lineage and c-opsins as sister to Group-4 opsins (Feuda et al. 2012, 2014; Fleming et al. 2020, 2021; De Vivo et al. 2023). Nevertheless, we also highlight that the evolutionary relationships, particularly between cnidarian and non-vertebrate deuterostome opsins, remain unresolved. This phylogenetic instability emphasizes the need for continued investigation into highly divergent opsin lineages and their evolutionary trajectories. A limitation of this study is that our findings are based on meta-omics data rather than full genomes or transcriptomes. Therefore, we were unable to produce a robust species tree and use methods to reconstruct the pattern of gene loss and duplication (such as implementing GeneRax; Morel et al. 2020), which would improve the inferred opsin evolutionary relationships.

In summary, while the classification of known opsin subfamilies appears robust and well-supported, continued investigation into the functional roles and evolutionary history of opsin diversity will be crucial to clarify their function as visual and non-visual proteins (Feuda et al. 2022).

## Methods

### Data Filtering

We initially mined opsin genes from a database containing about 158 million gene sequences derived from metagenomic and metatranscriptomic samples collected during the Tara Oceans and Tara Arctic expeditions (available at https://www.genoscope.cns.fr/tara/). Translated sequences from the Tara dataset, both genomic (SMAGs) and transcriptomic (MATOU-v1.5), were blasted (BLASTp) using Diamond against a precompiled opsin dataset representative of all known opsin subfamilies as a query composed of 50 sequences. As a result, 14,176 putative opsin sequences were obtained (SMAGs: 5,266; MATOU: 8,910).

To filter out all non-opsin sequences that were collected by the initial BLAST search and to check if potential new opsin gene families were present, we proceeded through the following steps: (i) The sequences were merged with a larger opsin dataset consisting of 35,745 sequences obtained from UniProt and NCBI. (ii) Sequences shorter than 150 amino acids were removed from the dataset. (iii) Only sequences with more than four and less than nine transmembrane domains (TM), as predicted by SCAMPI (Peters et al., 2016), were retained for subsequent analysis. After these steps, a total of 43,228 sequences remained, of which 7,836 were from the Tara dataset.

To classify putative opsin in Tara dataset and given the computational limitation of performing a phylogenetic analysis using 43,228 sequences, we opted to split the dataset into subgroups based on protein similarity. The rationale behind this approach is that by grouping sequences with similar proteins, we should be able to identify all Tara sequences that exhibit some level of similarity to known opsins. To achieve this goal, we conducted an all-versus-all BLASTp in Diamond (e-value threshold: 1e^−10^). Sequences were then clustered based on their similarity using the Markov Cluster Algorithm (van Dongen and Abreu-Goodger 2012) with an inflation value set to 1.4. Each cluster was annotated using Eggnog v5.0 (Huerta-Cepas et al. 2019), and only the clusters that contained at least an annotated opsin were retained. This filtering process resulted in 369 clusters out of 735, of which 127 contained at least 1 opsin from Tara and 16 clusters were exclusively from Tara. This resulted in 2,317 putative opsins (1,801 from MATOU and 516 from SMAG). To confirm the identity of these 2,317 sequences, we BLASTP against the NCBI database. A total of 223 sequences were discarded, leaving a final set of 2,091 opsin sequences for subsequent analysis. The entire pipeline is outlined in Fig. S1.

### Phylogenetic Analysis

For the phylogenetic analysis, we generated a combined dataset that included all 2,091 Tara opsin sequences and 738 previously annotated opsin sequences collected from NCBI, Fleming et al. (2021), and De Vivo et al. (2023), from which duplicates have been removed (Dataset 1; File S1). These reference sequences span the entirety of metazoan diversity, and comprise sequences belonging to acoelomates, vertebrates, lophotrochozoans (platyhelminths, bryozoans, annelids, mollusks, nemerteans brachiopods, and phoronids), ecdysozoans (arthropods, tardigrades, onychophorans, and priapulids), ctenophores, cnidarians, and placozoans, including melatonin receptors as outgroup (Feuda et al. 2012; De Vivo et al. 2023; McElroy et al. 2023). The datasets were aligned using MAFFT-DASH v7.3 (server https://mafft.cbrc.jp/alignment/software/, option mafft –averagelinkage –reorder –anysymbol –allowshift –unalignlevel 0.8 –dash –dashserver https://sysimm.ifrec.osaka-u.ac.jp/dash/REST1.0/–maxiterate 0 –globalpair input) and trimmed using TrimAL with gap threshold of 0.1 (Capella-Gutiérrez et al. 2009; Rozewicki et al. 2019). Finally, phylogenetic analyses have been performed using IQ-TREE2 (-m GRT20+G4 –B 1000) (Minh et al. 2020) and RogueNaRok (Aberer et al. 2013). GTR+G has shown to be the best fitting model in previous opsin studies and outperforms the others (Feuda et al. 2012; Fleming et al. 2021; De Vivo et al. 2023). Finally, taxonomic data were assigned by looking the tree topology and confirmed using reverse BLASTp on NCBI database.

Subsequently, we constructed a new dataset containing only r-opsin sequences from Arthropoda by integrating these with the visual opsin dataset from Fleming et al. (2021), resulting in Dataset 2 (File S2). To infer the taxonomic distribution of the arthropod opsins, we combined our phylogenetic results with the taxonomic annotations provided by the public available SMAGs dataset. The SMAG taxonomic annotations were generated using a combination of phylogenetic placement, average nucleotide identity (ANI), and gene marker comparisons, as described by Delmont et al. (2022). These annotations provide taxonomic resolution ranging from phylum to species, depending on the completeness of each metagenome-assembled genome (MAG).

Additionally, to address the potential impact of the large number of similar sequences from closely related organisms—especially arthropods—on the phylogenetic results (e.g. node support values), we made a third dataset. In this dataset (Dataset 3), we removed 95% of the Tara sequences assigned to arthropods (a total of 298 sequences from Tara datasets retained) from Dataset 1 (File S3). The only arthropod r-opsin dataset (Dataset 2, File S2) and the 95% removed arthropod dataset (Dataset 3, File S3) were used to perform phylogenetic analyses with IQ-TREE2 (-m GRT20+G4 –B 1000). To avoid local optima, we decreased smaller perturbation strength (-pers 0.2) and used a larger number of stop iterations (-nstop 500). Files S4 and S5 contain information of the taxonomic and sampling metadata as in the original MATOU-v1.5 and SMAGs dataset (Sunagawa et al. 2020). The Newick and .log files of all the phylogenetic analyses can be found in File S6.

## Supplementary Material

evaf189_Supplementary_Data

## Data Availability

Data are available on GitHub: https://github.com/Xodroont/Tara_Opsins. MATOU-v1.5 and SMAGs data are available at http://www.genoscope.cns.fr/tara/.
