# Supplementary figures and images for: An Ocean of Opsins
Source: Genome Biol Evol. 2025 Nov 4;17(11):evaf189. doi: 10.1093/gbe/evaf189 (PMC12584886; doi:10.1093/gbe/evaf189)

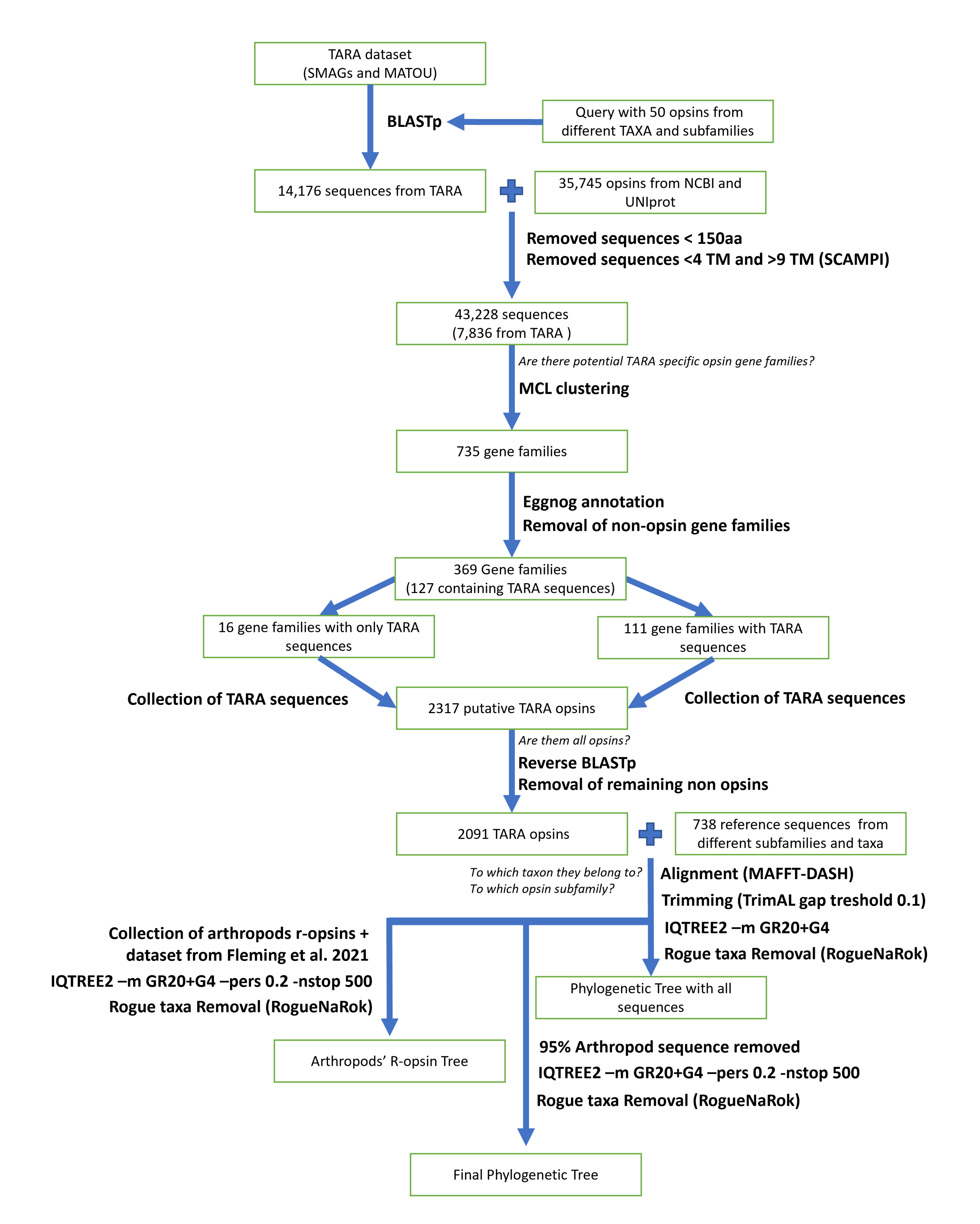

Supplement: evaf189_Supplementary_Data [file evaf189_supplementary_data.zip › Figure_S1.png]
