# Supplementary material for: An Ocean of Opsins
Source: Genome Biol Evol. 2025 Nov 4;17(11):evaf189. doi: 10.1093/gbe/evaf189 (PMC12584886; doi:10.1093/gbe/evaf189)

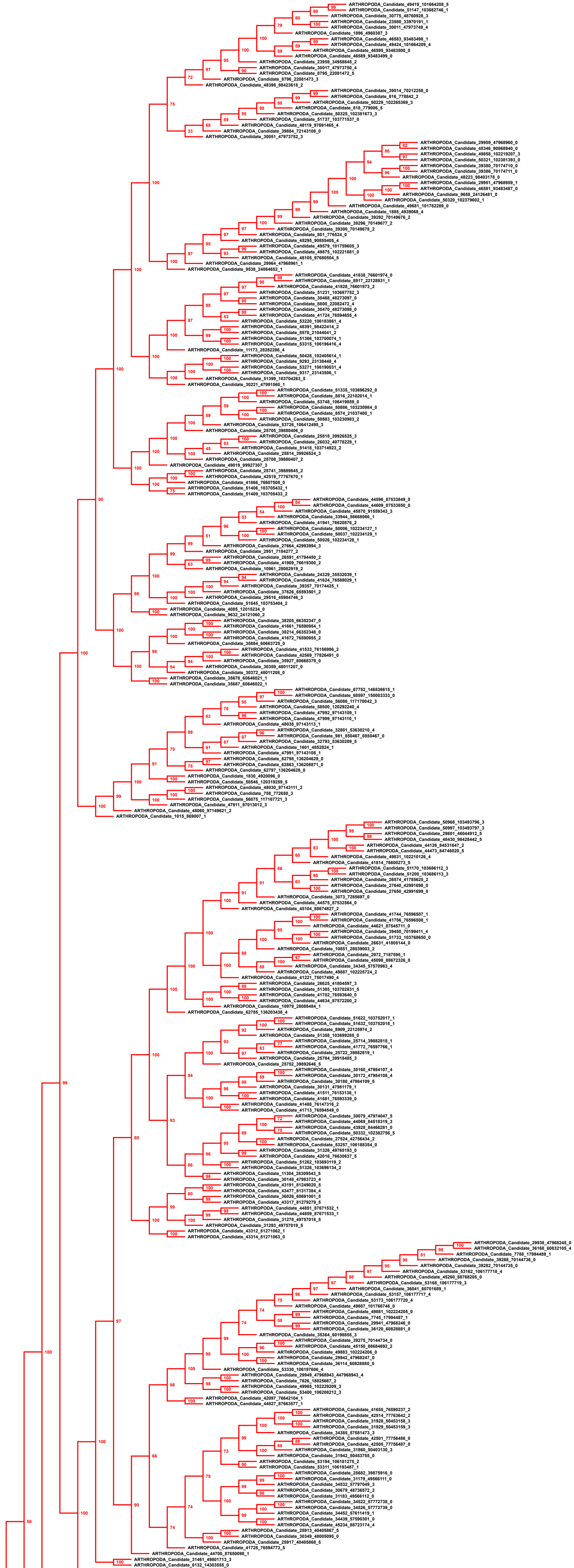

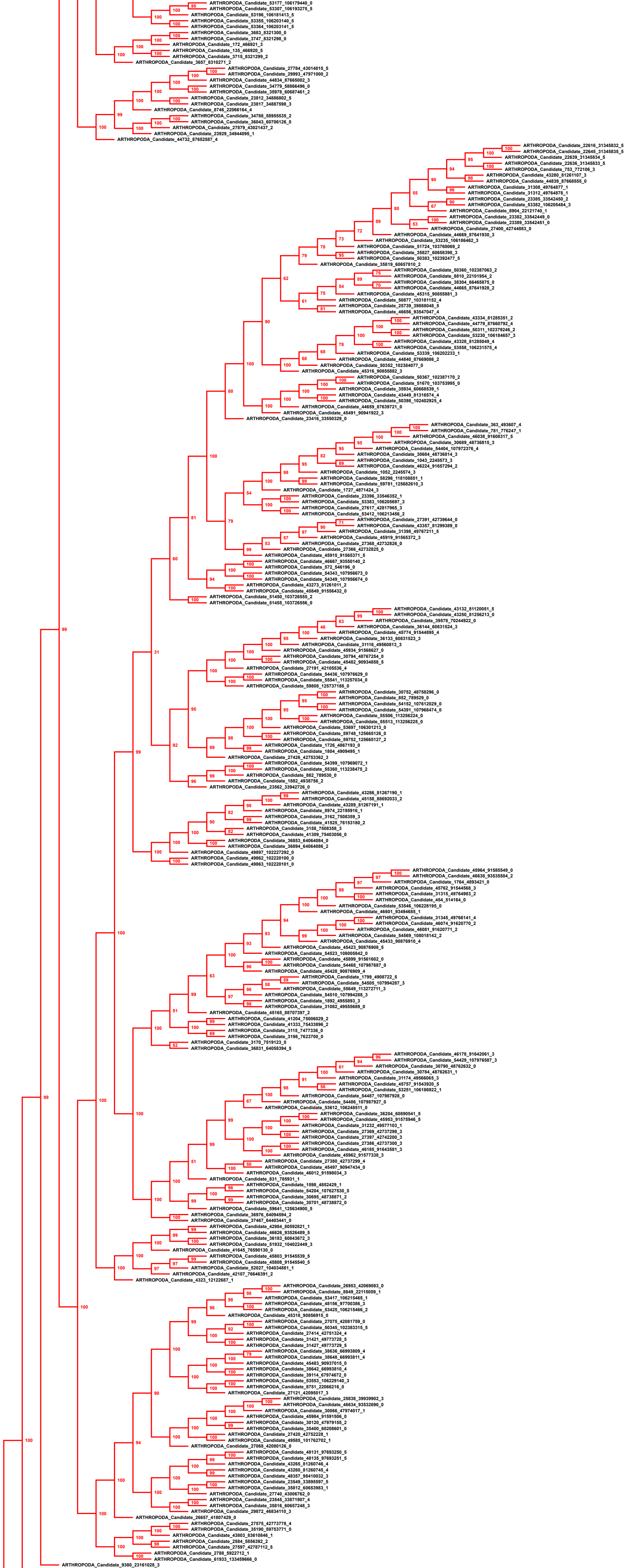

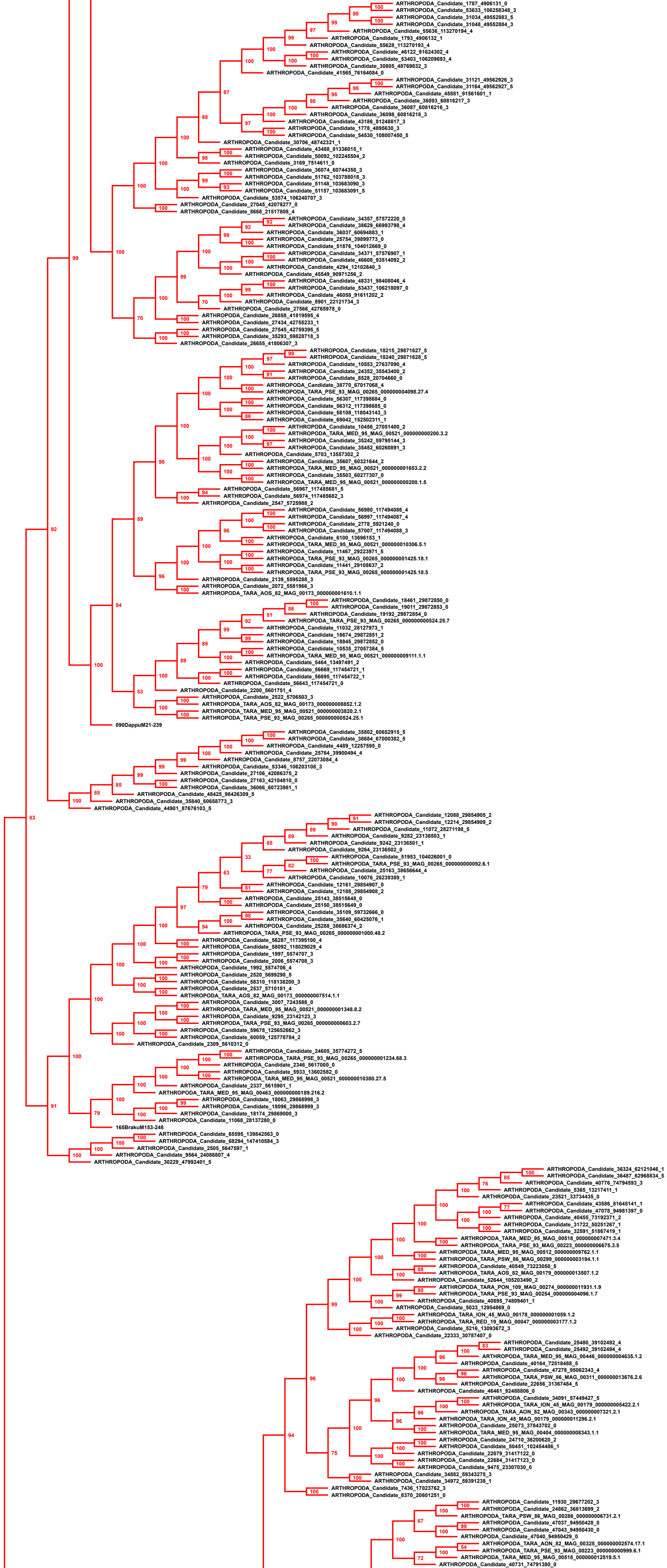

ARTHROPODA_Candidate_1787_4906131_0
ARTHROPODA_Candidate_53633_106258348_3
ARTHROPODA_Candidate_31034_49552883_5
ARTHROPODA_Candidate_31048_49552884_3
ARTHROPODA_Candidate_55636_113270194_4
ARTHROPODA_Candidate_1793_4906132_1
ARTHROPODA_Candidate_55628_113270193_4
ARTHROPODA_Candidate_46122_91624302_4
ARTHROPODA_Candidate_53403_106209693_4
ARTHROPODA_Candidate_30805_48769832_3
ARTHROPODA_Candidate_41565_76164084_0
ARTHROPODA_Candidate_31121_49562926_3
ARTHROPODA_Candidate_31164_49562927_5
ARTHROPODA_Candidate_45881_91561601_1
ARTHROPODA_Candidate_36093_60816217_3
ARTHROPODA_Candidate_36087_60816216_3
ARTHROPODA_Candidate_36098_60816218_3
ARTHROPODA_Candidate_43186_81248817_3
ARTHROPODA_Candidate_1778_4895630_3
ARTHROPODA_Candidate_54530_108007450_5
ARTHROPODA_Candidate_30706_48742321_1
ARTHROPODA_Candidate_43488_81336015_1
ARTHROPODA_Candidate_50092_102245504_2
ARTHROPODA_Candidate_3169_7514611_0
ARTHROPODA_Candidate_36074_60744358_3
ARTHROPODA_Candidate_51762_103788018_3
ARTHROPODA_Candidate_51148_103683090_3
ARTHROPODA_Candidate_51157_103683091_5
ARTHROPODA_Candidate_53574_106240707_3
ARTHROPODA_Candidate_27045_42078277_0
ARTHROPODA_Candidate_8668_21517808_4
ARTHROPODA_Candidate_34357_57572220_0
ARTHROPODA_Candidate_38629_66993798_4
ARTHROPODA_Candidate_36037_60694883_1
ARTHROPODA_Candidate_25754_39899773_0
ARTHROPODA_Candidate_51876_104012669_0
ARTHROPODA_Candidate_34371_57576907_1
ARTHROPODA_Candidate_46608_93514092_2
ARTHROPODA_Candidate_4294_12102640_3
ARTHROPODA_Candidate_45549_90971256_2
ARTHROPODA_Candidate_48331_98408046_4
ARTHROPODA_Candidate_53437_106218097_0
ARTHROPODA_Candidate_46058_91611202_2
ARTHROPODA_Candidate_8901_22121734_3
ARTHROPODA_Candidate_27566_42765978_0
ARTHROPODA_Candidate_26858_41819595_4
ARTHROPODA_Candidate_27434_42755233_1
ARTHROPODA_Candidate_27545_42759395_5
ARTHROPODA_Candidate_35293_59828718_3
ARTHROPODA_Candidate_26655_41806307_3
ARTHROPODA_Candidate_18215_29871627_5
ARTHROPODA_Candidate_18240_29871628_5
ARTHROPODA_Candidate_10553_27637090_4
ARTHROPODA_Candidate_24352_35543400_2
ARTHROPODA_Candidate_8528_20704660_0
ARTHROPODA_Candidate_38770_67017068_4
ARTHROPODA_TARA_PSE_93_MAG_00265_000000004098.27.4
ARTHROPODA_Candidate_56307_117398684_0
ARTHROPODA_Candidate_56312_117398685_0
ARTHROPODA_Candidate_58108_118043143_3
ARTHROPODA_Candidate_69042_152502311_1
ARTHROPODA_Candidate_10456_27051400_2
ARTHROPODA_TARA_MED_95_MAG_00521_000000000200.3.2
ARTHROPODA_Candidate_35242_59795144_3
ARTHROPODA_Candidate_35452_60260891_3
ARTHROPODA_Candidate_5703_13557302_2
ARTHROPODA_Candidate_35607_60321644_2
ARTHROPODA_TARA_MED_95_MAG_00521_000000001653.2.2
ARTHROPODA_Candidate_35503_60277307_0
ARTHROPODA_TARA_MED_95_MAG_00521_000000000200.1.5
ARTHROPODA_Candidate_56967_117485681_5
ARTHROPODA_Candidate_56974_117485682_3
ARTHROPODA_Candidate_2547_5725988_2
ARTHROPODA_Candidate_56980_117494086_4
ARTHROPODA_Candidate_56997_117494087_4
ARTHROPODA_Candidate_2778_5921240_0
ARTHROPODA_Candidate_57007_117494088_3
ARTHROPODA_Candidate_6100_13696153_1
ARTHROPODA_TARA_MED_95_MAG_00521_000000010306.5.1
ARTHROPODA_Candidate_11467_29223971_5
ARTHROPODA_TARA_PSE_93_MAG_00265_000000001425.18.1
ARTHROPODA_Candidate_11441_29108637_2
ARTHROPODA_TARA_PSE_93_MAG_00265_000000001425.18.5
ARTHROPODA_Candidate_2139_5595288_3
ARTHROPODA_Candidate_2072_5581966_3
ARTHROPODA_TARA_AOS_82_MAG_00173_000000001610.1.1
ARTHROPODA_Candidate_18461_29872850_0
ARTHROPODA_Candidate_19011_29872853_0
ARTHROPODA_Candidate_19192_29872854_0
ARTHROPODA_TARA_PSE_93_MAG_00265_000000000524.25.7
ARTHROPODA_Candidate_11032_28127973_1
ARTHROPODA_Candidate_18674_29872851_2
ARTHROPODA_Candidate_18845_29872852_0
ARTHROPODA_Candidate_10535_27057384_5
ARTHROPODA_TARA_MED_95_MAG_00521_000000009111.1.1
ARTHROPODA_Candidate_5464_13497491_2
ARTHROPODA_Candidate_56669_117454721_1
ARTHROPODA_Candidate_56695_117454722_1
ARTHROPODA_Candidate_56643_117454721_0
ARTHROPODA_Candidate_2200_5601751_4
ARTHROPODA_Candidate_2522_5706503_3
ARTHROPODA_TARA_AOS_82_MAG_00173_000000008852.1.2
ARTHROPODA_TARA_MED_95_MAG_00521_000000003820.2.1
ARTHROPODA_TARA_PSE_93_MAG_00265_000000000524.25.1
090DappuM21-239
ARTHROPODA_Candidate_35802_60652915_5
ARTHROPODA_Candidate_38684_67000382_5
ARTHROPODA_Candidate_4489_12257595_0
ARTHROPODA_Candidate_25764_39900494_4
ARTHROPODA_Candidate_8757_22073084_4
ARTHROPODA_Candidate_53346_106203108_3
ARTHROPODA_Candidate_27106_42086375_2
ARTHROPODA_Candidate_27163_42104810_0
ARTHROPODA_Candidate_36066_60723981_1
ARTHROPODA_Candidate_48425_98426309_5
ARTHROPODA_Candidate_35840_60658773_3
ARTHROPODA_Candidate_44901_87676103_5
ARTHROPODA_Candidate_12088_29854905_2
ARTHROPODA_Candidate_12214_29854909_2
ARTHROPODA_Candidate_11072_28271198_5
ARTHROPODA_Candidate_9282_23136503_1
ARTHROPODA_Candidate_9242_23136501_1
ARTHROPODA_Candidate_9264_23136502_0
ARTHROPODA_Candidate_51953_104026001_0
ARTHROPODA_TARA_PSE_93_MAG_00265_000000000092.6.1
ARTHROPODA_Candidate_25163_38656644_4
ARTHROPODA_Candidate_10076_26239389_1
ARTHROPODA_Candidate_12161_29854907_0
ARTHROPODA_Candidate_12188_29854908_2
ARTHROPODA_Candidate_25143_38515648_0
ARTHROPODA_Candidate_25150_38515649_0
ARTHROPODA_Candidate_35109_59732666_0
ARTHROPODA_Candidate_35640_60425076_1
ARTHROPODA_Candidate_25288_38686374_2
ARTHROPODA_TARA_PSE_93_MAG_00265_000000001000.48.2
ARTHROPODA_Candidate_56287_117395100_4
ARTHROPODA_Candidate_58092_118029029_4
ARTHROPODA_Candidate_1997_5574707_3
ARTHROPODA_Candidate_2006_5574708_3
ARTHROPODA_Candidate_1992_5574706_4
ARTHROPODA_Candidate_2520_5699298_5
ARTHROPODA_Candidate_58310_118138200_3
ARTHROPODA_Candidate_2537_5710181_4
ARTHROPODA_TARA_AOS_82_MAG_00173_000000007514.1.1
ARTHROPODA_Candidate_3007_7243588_0
ARTHROPODA_TARA_MED_95_MAG_00521_000000001348.8.2
ARTHROPODA_Candidate_9295_23142123_3
ARTHROPODA_TARA_PSE_93_MAG_00265_000000000603.2.7
ARTHROPODA_Candidate_59678_125652662_3
ARTHROPODA_Candidate_60059_125778784_2
ARTHROPODA_Candidate_2309_5610312_0
ARTHROPODA_Candidate_24605_35774272_5
ARTHROPODA_TARA_PSE_93_MAG_00265_000000001234.68.3
ARTHROPODA_Candidate_2346_5617000_0
ARTHROPODA_Candidate_5933_13602582_0
ARTHROPODA_TARA_MED_95_MAG_00521_000000010380.27.5
ARTHROPODA_Candidate_2337_5615901_1
ARTHROPODA_TARA_MED_95_MAG_00463_000000000189.216.2
ARTHROPODA_Candidate_18063_29868998_3
ARTHROPODA_Candidate_18096_29868999_3
ARTHROPODA_Candidate_18174_29869000_3
ARTHROPODA_Candidate_11068_28137280_0
165BrakuM153-248
ARTHROPODA_Candidate_65595_139842563_0
ARTHROPODA_Candidate_68294_147410584_3
ARTHROPODA_Candidate_2505_5647597_1
ARTHROPODA_Candidate_9564_24086807_4
ARTHROPODA_Candidate_30229_47992401_5
ARTHROPODA_Candidate_36324_62121046_1
ARTHROPODA_Candidate_36487_62968834_5
ARTHROPODA_Candidate_40776_74794593_3
ARTHROPODA_Candidate_5365_13217411_1
ARTHROPODA_Candidate_23521_33734435_0
ARTHROPODA_Candidate_43586_81645141_1
ARTHROPODA_Candidate_47078_94981397_0
ARTHROPODA_Candidate_40455_73192371_2
ARTHROPODA_Candidate_31722_50251267_1
ARTHROPODA_Candidate_32591_51867419_1
ARTHROPODA_TARA_MED_95_MAG_00518_000000007471.3.4
ARTHROPODA_TARA_PSE_93_MAG_00223_000000006675.3.5
ARTHROPODA_TARA_MED_95_MAG_00512_000000009762.1.1
ARTHROPODA_TARA_PSW_86_MAG_00299_000000003194.1.1
ARTHROPODA_Candidate_40549_73223050_5
ARTHROPODA_TARA_AOS_82_MAG_00179_000000013507.1.2
ARTHROPODA_Candidate_52644_105203490_2
ARTHROPODA_TARA_PON_109_MAG_00274_000000011931.1.9
ARTHROPODA_TARA_PSE_93_MAG_00254_000000004096.1.7
ARTHROPODA_Candidate_40895_74809401_1
ARTHROPODA_Candidate_5033_12954869_0
ARTHROPODA_TARA_ION_45_MAG_00178_000000001059.1.2
ARTHROPODA_TARA_RED_19_MAG_00047_000000003177.1.2
ARTHROPODA_Candidate_5216_13093672_3
ARTHROPODA_Candidate_22333_30787407_0
ARTHROPODA_Candidate_25480_39102492_4
ARTHROPODA_Candidate_25492_39102494_4
ARTHROPODA_TARA_MED_95_MAG_00446_000000004635.1.2
ARTHROPODA_Candidate_40164_72518488_5
ARTHROPODA_Candidate_47278_95062343_4
ARTHROPODA_TARA_PSW_86_MAG_00311_000000013676.2.6
ARTHROPODA_Candidate_22656_31367484_5
ARTHROPODA_Candidate_46461_92488806_0
ARTHROPODA_Candidate_34091_57449427_5
ARTHROPODA_TARA_ION_45_MAG_00179_000000005422.2.1
ARTHROPODA_TARA_AON_82_MAG_00343_000000007321.2.1
ARTHROPODA_TARA_ION_45_MAG_00179_000000011296.2.1
ARTHROPODA_Candidate_25073_37843702_0
ARTHROPODA_TARA_MED_95_MAG_00404_000000008343.1.1
ARTHROPODA_Candidate_24710_36200620_2
ARTHROPODA_Candidate_50451_102454486_1
ARTHROPODA_Candidate_22679_31417122_0
ARTHROPODA_Candidate_22684_31417123_0
ARTHROPODA_Candidate_9475_23307030_0
ARTHROPODA_Candidate_34882_59343275_3
ARTHROPODA_Candidate_34972_59391238_1
ARTHROPODA_Candidate_7436_17023762_3
ARTHROPODA_Candidate_8370_20601251_0
ARTHROPODA_Candidate_11930_29677202_3
ARTHROPODA_Candidate_24862_36813699_2
ARTHROPODA_TARA_PSW_86_MAG_00286_000000006731.2.1
ARTHROPODA_Candidate_47037_94950428_0
ARTHROPODA_Candidate_47043_94950430_0
ARTHROPODA_Candidate_47040_94950429_0
ARTHROPODA_TARA_AON_82_MAG_00325_000000002574.17.1
ARTHROPODA_TARA_PSE_93_MAG_00223_000000000999.6.1
ARTHROPODA_TARA_MED_95_MAG_00518_000000012519.5.1
ARTHROPODA_Candidate_40731_74791380_0

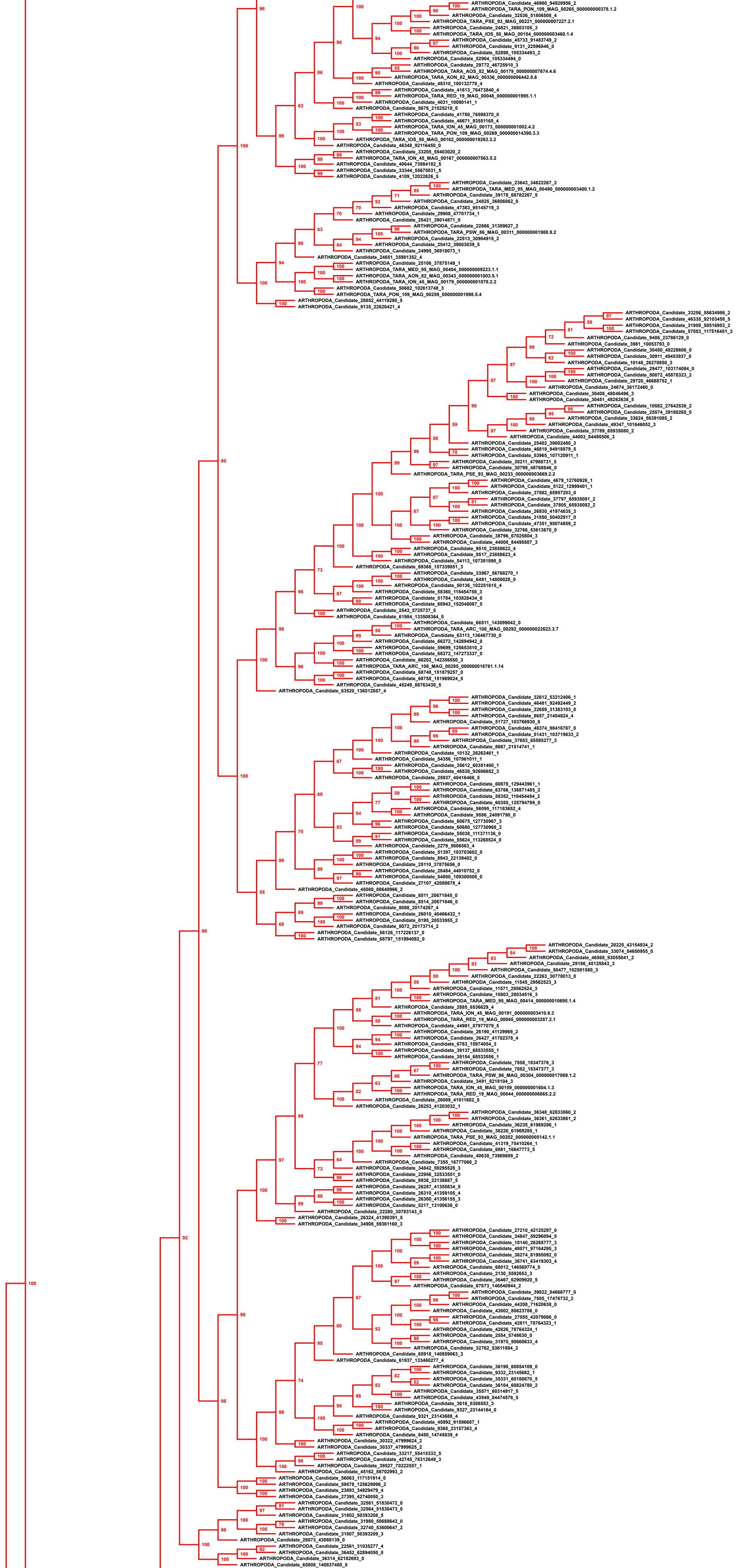

ARTHROPODA_Candidate_46980_94928956_2
ARTHROPODA_TARA_PON_109_MAG_00265_000000000378.1.2
ARTHROPODA_Candidate_32536_51806508_4
ARTHROPODA_TARA_PSE_93_MAG_00221_000000007227.2.1
ARTHROPODA_Candidate_24821_36803105_3
ARTHROPODA_TARA_IOS_50_MAG_00154_000000003460.1.4
ARTHROPODA_Candidate_45733_91483749_2
ARTHROPODA_Candidate_9131_22596846_0
ARTHROPODA_Candidate_52898_105334493_2
ARTHROPODA_Candidate_52904_105334494_0
ARTHROPODA_Candidate_29772_46725910_3
ARTHROPODA_TARA_AOS_82_MAG_00179_000000007674.4.6
ARTHROPODA_TARA_AON_82_MAG_00336_000000006442.5.6
ARTHROPODA_Candidate_49310_100132779_4
ARTHROPODA_Candidate_41613_76473840_4
ARTHROPODA_TARA_RED_19_MAG_00048_000000001995.1.1
ARTHROPODA_Candidate_4031_10090141_1
ARTHROPODA_Candidate_8678_21525218_0
ARTHROPODA_Candidate_41780_76598370_0
ARTHROPODA_Candidate_46671_93551165_4
ARTHROPODA_TARA_ION_45_MAG_00173_000000001002.4.2
ARTHROPODA_TARA_PON_109_MAG_00269_000000014390.3.3
ARTHROPODA_TARA_IOS_50_MAG_00162_000000019263.3.2
ARTHROPODA_Candidate_46348_92116450_0
ARTHROPODA_Candidate_33205_55403020_2
ARTHROPODA_TARA_ION_45_MAG_00167_000000007563.5.2
ARTHROPODA_Candidate_40644_73984182_5
ARTHROPODA_Candidate_33344_55675031_5
ARTHROPODA_Candidate_4109_12022626_5
ARTHROPODA_Candidate_23643_34622267_2
ARTHROPODA_TARA_MED_95_MAG_00490_000000003400.1.2
ARTHROPODA_Candidate_39178_68782267_5
ARTHROPODA_Candidate_24825_36806062_0
ARTHROPODA_Candidate_47383_95145719_3
ARTHROPODA_Candidate_29908_47701734_1
ARTHROPODA_Candidate_25421_39014871_0
ARTHROPODA_Candidate_22666_31389627_2
ARTHROPODA_TARA_PSW_86_MAG_00311_000000001988.9.2
ARTHROPODA_Candidate_22513_30904915_2
ARTHROPODA_Candidate_25412_39003039_5
ARTHROPODA_Candidate_24995_36918073_1
ARTHROPODA_Candidate_24651_35981352_4
ARTHROPODA_Candidate_25106_37875149_1
ARTHROPODA_TARA_MED_95_MAG_00404_000000009223.1.1
ARTHROPODA_TARA_AON_82_MAG_00343_000000001003.5.1
ARTHROPODA_TARA_ION_45_MAG_00179_000000001078.2.2
ARTHROPODA_Candidate_50682_102613746_3
ARTHROPODA_TARA_PON_109_MAG_00259_000000001998.5.4
ARTHROPODA_Candidate_28852_44119290_5
ARTHROPODA_Candidate_9135_22620421_4
ARTHROPODA_Candidate_33256_55634986_2
ARTHROPODA_Candidate_46335_92103458_5
ARTHROPODA_Candidate_31959_50516953_2
ARTHROPODA_Candidate_57053_117516451_3
ARTHROPODA_Candidate_9486_23786129_0
ARTHROPODA_Candidate_3981_10053793_0
ARTHROPODA_Candidate_30450_48228606_0
ARTHROPODA_Candidate_30911_49453937_0
ARTHROPODA_Candidate_10148_26270850_3
ARTHROPODA_Candidate_29477_103174084_0
ARTHROPODA_Candidate_50872_45878323_2
ARTHROPODA_Candidate_29720_46688752_1
ARTHROPODA_Candidate_24674_36172460_0
ARTHROPODA_Candidate_30408_48046496_3
ARTHROPODA_Candidate_30451_48262638_5
ARTHROPODA_Candidate_10582_27642538_2
ARTHROPODA_Candidate_25574_39180265_0
ARTHROPODA_Candidate_33624_56391085_2
ARTHROPODA_Candidate_49347_101646052_3
ARTHROPODA_Candidate_37789_65935080_2
ARTHROPODA_Candidate_44003_84495506_3
ARTHROPODA_Candidate_25402_39002480_3
ARTHROPODA_Candidate_46818_94918879_5
ARTHROPODA_Candidate_53965_107120911_1
ARTHROPODA_Candidate_30211_47988731_5
ARTHROPODA_Candidate_30799_48768846_0
ARTHROPODA_TARA_PSE_93_MAG_00233_000000003669.2.2
ARTHROPODA_Candidate_4679_12760926_1
ARTHROPODA_Candidate_5122_12999401_1
ARTHROPODA_Candidate_37882_65957203_0
ARTHROPODA_Candidate_37797_65935081_2
ARTHROPODA_Candidate_37805_65935082_2
ARTHROPODA_Candidate_26930_41974835_3
ARTHROPODA_Candidate_31850_50402917_0
ARTHROPODA_Candidate_47351_95074859_2
ARTHROPODA_Candidate_32766_53613670_0
ARTHROPODA_Candidate_38796_67025804_3
ARTHROPODA_Candidate_44008_84495507_3
ARTHROPODA_Candidate_9510_23858622_4
ARTHROPODA_Candidate_9517_23858623_4
ARTHROPODA_Candidate_54113_107381898_0
ARTHROPODA_Candidate_69368_157339851_3
ARTHROPODA_Candidate_33967_56768270_1
ARTHROPODA_Candidate_6491_14800028_0
ARTHROPODA_Candidate_50136_102251615_4
ARTHROPODA_Candidate_58360_118454758_3
ARTHROPODA_Candidate_51784_103828434_0
ARTHROPODA_Candidate_68943_152048087_5
ARTHROPODA_Candidate_2543_5725737_5
ARTHROPODA_Candidate_61984_133508364_5
ARTHROPODA_Candidate_66511_143099042_0
ARTHROPODA_TARA_ARC_108_MAG_00292_000000022623.3.7
ARTHROPODA_Candidate_63113_136467730_0
ARTHROPODA_Candidate_66272_142694942_0
ARTHROPODA_Candidate_59699_125653510_2
ARTHROPODA_Candidate_68272_147273337_0
ARTHROPODA_Candidate_66202_142356550_3
ARTHROPODA_TARA_ARC_108_MAG_00295_000000016761.1.14
ARTHROPODA_Candidate_68748_151879257_0
ARTHROPODA_Candidate_68758_151969024_5
ARTHROPODA_Candidate_45249_88763430_5
ARTHROPODA_Candidate_63520_136512857_4
ARTHROPODA_Candidate_32612_53212406_1
ARTHROPODA_Candidate_46481_92492449_2
ARTHROPODA_Candidate_22659_31383103_0
ARTHROPODA_Candidate_8657_21404824_4
ARTHROPODA_Candidate_51727_103766930_0
ARTHROPODA_Candidate_48374_98416767_0
ARTHROPODA_Candidate_51431_103719633_2
ARTHROPODA_Candidate_37653_65595277_3
ARTHROPODA_Candidate_8667_21514741_1
ARTHROPODA_Candidate_10132_26262461_1
ARTHROPODA_Candidate_54356_107961011_1
ARTHROPODA_Candidate_35612_60351400_1
ARTHROPODA_Candidate_46538_92606652_3
ARTHROPODA_Candidate_25937_40416466_5
ARTHROPODA_Candidate_60878_129443961_1
ARTHROPODA_Candidate_63766_136871485_2
ARTHROPODA_Candidate_58352_118454484_2
ARTHROPODA_Candidate_60355_125794799_0
ARTHROPODA_Candidate_56095_117183652_4
ARTHROPODA_Candidate_9586_24091790_0
ARTHROPODA_Candidate_60675_127730967_3
ARTHROPODA_Candidate_60680_127730968_3
ARTHROPODA_Candidate_55038_111371136_0
ARTHROPODA_Candidate_55624_113268524_0
ARTHROPODA_Candidate_2279_5606563_4
ARTHROPODA_Candidate_51397_103703602_0
ARTHROPODA_Candidate_8943_22139402_0
ARTHROPODA_Candidate_25110_37875656_0
ARTHROPODA_Candidate_28484_44010752_0
ARTHROPODA_Candidate_54800_109300508_0
ARTHROPODA_Candidate_27107_42088678_4
ARTHROPODA_Candidate_45088_88640966_2
ARTHROPODA_Candidate_8511_20671845_0
ARTHROPODA_Candidate_8514_20671846_0
ARTHROPODA_Candidate_8088_20174267_4
ARTHROPODA_Candidate_26010_40466432_1
ARTHROPODA_Candidate_8195_20533955_2
ARTHROPODA_Candidate_8072_20173714_2
ARTHROPODA_Candidate_56126_117226137_0
ARTHROPODA_Candidate_68797_151994092_0
ARTHROPODA_Candidate_28225_43154934_2
ARTHROPODA_Candidate_33074_54650955_0
ARTHROPODA_Candidate_46568_93055041_2
ARTHROPODA_Candidate_29186_45125843_3
ARTHROPODA_Candidate_50477_102581580_3
ARTHROPODA_Candidate_22263_30778013_0
ARTHROPODA_Candidate_11545_29562523_3
ARTHROPODA_Candidate_11571_29562524_3
ARTHROPODA_Candidate_10803_28034516_3
ARTHROPODA_TARA_MED_95_MAG_00414_000000010695.1.4
ARTHROPODA_Candidate_2885_6536629_4
ARTHROPODA_TARA_ION_45_MAG_00191_000000003410.8.2
ARTHROPODA_TARA_RED_19_MAG_00045_000000003287.2.1
ARTHROPODA_Candidate_44981_87977079_5
ARTHROPODA_Candidate_26190_41129969_2
ARTHROPODA_Candidate_26427_41702378_4
ARTHROPODA_Candidate_6783_15974054_3
ARTHROPODA_Candidate_39137_68533555_1
ARTHROPODA_Candidate_39154_68533556_1
ARTHROPODA_Candidate_7858_18347376_3
ARTHROPODA_Candidate_7882_18347377_3
ARTHROPODA_TARA_PSW_86_MAG_00304_000000017069.1.2
ARTHROPODA_Candidate_3491_8218104_3
ARTHROPODA_TARA_ION_45_MAG_00159_000000001604.1.3
ARTHROPODA_TARA_RED_19_MAG_00044_000000006865.2.2
ARTHROPODA_Candidate_26089_41011682_5
ARTHROPODA_Candidate_26253_41203032_1
ARTHROPODA_Candidate_36348_62833860_2
ARTHROPODA_Candidate_36361_62833861_2
ARTHROPODA_Candidate_36235_61969286_1
ARTHROPODA_Candidate_36226_61969285_1
ARTHROPODA_TARA_PSE_93_MAG_00202_000000005142.1.1
ARTHROPODA_Candidate_41319_75410264_1
ARTHROPODA_Candidate_6881_16647773_5
ARTHROPODA_Candidate_40638_73969809_2
ARTHROPODA_Candidate_7355_16777068_2
ARTHROPODA_Candidate_34842_59295528_3
ARTHROPODA_Candidate_22956_32533551_0
ARTHROPODA_Candidate_8938_22138687_5
ARTHROPODA_Candidate_26287_41355834_5
ARTHROPODA_Candidate_26310_41359105_4
ARTHROPODA_Candidate_26300_41356155_3
ARTHROPODA_Candidate_5217_13100636_0
ARTHROPODA_Candidate_22280_30783143_0
ARTHROPODA_Candidate_26324_41390391_5
ARTHROPODA_Candidate_34908_59361160_3
ARTHROPODA_Candidate_27210_42125297_0
ARTHROPODA_Candidate_34847_59296094_5
ARTHROPODA_Candidate_10140_26265777_3
ARTHROPODA_Candidate_48071_97164295_3
ARTHROPODA_Candidate_36274_61985092_0
ARTHROPODA_Candidate_36741_63419303_4
ARTHROPODA_Candidate_68012_146569774_0
ARTHROPODA_Candidate_2130_5592653_3
ARTHROPODA_Candidate_36467_62909920_5
ARTHROPODA_Candidate_67873_146540944_2
ARTHROPODA_Candidate_39822_84666777_0
ARTHROPODA_Candidate_7505_17476732_2
ARTHROPODA_Candidate_44208_71620638_0
ARTHROPODA_Candidate_43002_80623786_0
ARTHROPODA_Candidate_27055_42079066_0
ARTHROPODA_Candidate_42811_78764323_1
ARTHROPODA_Candidate_42826_78764324_1
ARTHROPODA_Candidate_2554_5748630_0
ARTHROPODA_Candidate_31975_50660633_4
ARTHROPODA_Candidate_32762_53611884_2
ARTHROPODA_Candidate_65918_140859063_3
ARTHROPODA_Candidate_61937_133460277_4
ARTHROPODA_Candidate_36190_60854109_0
ARTHROPODA_Candidate_9332_23145682_1
ARTHROPODA_Candidate_35331_60188670_5
ARTHROPODA_Candidate_36104_60824780_2
ARTHROPODA_Candidate_35571_60314917_5
ARTHROPODA_Candidate_43949_84474576_5
ARTHROPODA_Candidate_3616_8308553_3
ARTHROPODA_Candidate_9327_23144164_0
ARTHROPODA_Candidate_9321_23143688_4
ARTHROPODA_Candidate_45992_91596687_1
ARTHROPODA_Candidate_9365_23157363_4
ARTHROPODA_Candidate_6450_14745839_4
ARTHROPODA_Candidate_30322_47999624_2
ARTHROPODA_Candidate_30337_47999625_2
ARTHROPODA_Candidate_33217_55415333_5
ARTHROPODA_Candidate_42745_78312649_3
ARTHROPODA_Candidate_39527_70222557_1
ARTHROPODA_Candidate_45162_88702993_2
ARTHROPODA_Candidate_56063_117151914_0
ARTHROPODA_Candidate_59578_125629996_2
ARTHROPODA_Candidate_23893_34929479_4
ARTHROPODA_Candidate_27395_42740350_3
ARTHROPODA_Candidate_32561_51830472_0
ARTHROPODA_Candidate_32564_51830473_0
ARTHROPODA_Candidate_31802_50393208_5
ARTHROPODA_Candidate_31980_50688643_0
ARTHROPODA_Candidate_32740_53600647_2
ARTHROPODA_Candidate_31807_50393209_3
ARTHROPODA_Candidate_28073_43088139_0
ARTHROPODA_Candidate_22561_31035277_4
ARTHROPODA_Candidate_36452_62894050_0
ARTHROPODA_Candidate_36314_62102693_0
ARTHROPODA_Candidate_65808_140837480_5

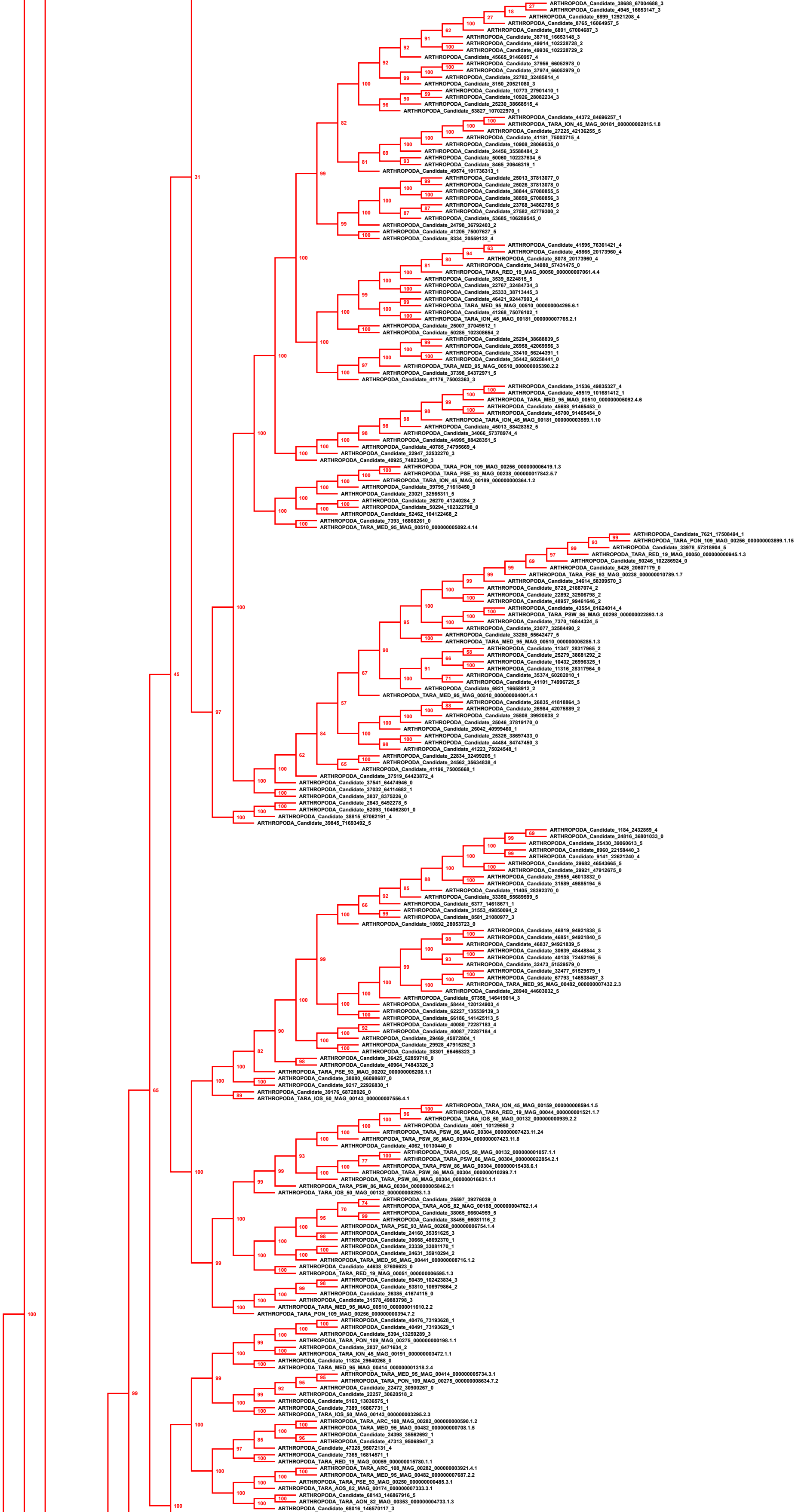

ARTHROPODA_Candidate_38688_67004688_3
ARTHROPODA_Candidate_4945_16653147_3
ARTHROPODA_Candidate_6899_12921208_4
ARTHROPODA_Candidate_8765_16064957_5
ARTHROPODA_Candidate_6891_67004687_3
ARTHROPODA_Candidate_38716_16653148_3
ARTHROPODA_Candidate_49914_102228728_2
ARTHROPODA_Candidate_49936_102228729_2
ARTHROPODA_Candidate_45665_91460957_4
ARTHROPODA_Candidate_37956_66052978_0
ARTHROPODA_Candidate_37974_66052979_0
ARTHROPODA_Candidate_22782_32485814_4
ARTHROPODA_Candidate_8150_20521080_3
ARTHROPODA_Candidate_10773_27901410_1
ARTHROPODA_Candidate_10926_28082234_3
ARTHROPODA_Candidate_25230_38668515_4
ARTHROPODA_Candidate_53827_107022970_1
ARTHROPODA_Candidate_44372_84696257_1
ARTHROPODA_TARA_ION_45_MAG_00181_000000002815.1.8
ARTHROPODA_Candidate_27225_42136255_5
ARTHROPODA_Candidate_41181_75003715_4
ARTHROPODA_Candidate_10908_28069535_0
ARTHROPODA_Candidate_24456_35588484_2
ARTHROPODA_Candidate_50060_102237634_5
ARTHROPODA_Candidate_8465_20646319_1
ARTHROPODA_Candidate_49574_101736313_1
ARTHROPODA_Candidate_25013_37813077_0
ARTHROPODA_Candidate_25026_37813078_0
ARTHROPODA_Candidate_38844_67080855_5
ARTHROPODA_Candidate_38859_67080856_3
ARTHROPODA_Candidate_23768_34862785_5
ARTHROPODA_Candidate_27582_42779300_2
ARTHROPODA_Candidate_53685_106289545_0
ARTHROPODA_Candidate_24798_36792403_2
ARTHROPODA_Candidate_41205_75007627_5
ARTHROPODA_Candidate_8334_20559132_4
ARTHROPODA_Candidate_41595_76361421_4
ARTHROPODA_Candidate_49865_20173960_4
ARTHROPODA_Candidate_8078_20173960_4
ARTHROPODA_Candidate_34080_57431475_0
ARTHROPODA_TARA_RED_19_MAG_00050_000000007061.4.4
ARTHROPODA_Candidate_3539_8224815_5
ARTHROPODA_Candidate_22767_32484734_3
ARTHROPODA_Candidate_25333_38713445_3
ARTHROPODA_Candidate_46421_92447993_4
ARTHROPODA_TARA_MED_95_MAG_00510_000000004295.6.1
ARTHROPODA_Candidate_41268_75076102_1
ARTHROPODA_TARA_ION_45_MAG_00181_000000007765.2.1
ARTHROPODA_Candidate_25007_37049512_1
ARTHROPODA_Candidate_50285_102308654_2
ARTHROPODA_Candidate_25294_38688839_5
ARTHROPODA_Candidate_26958_42069956_3
ARTHROPODA_Candidate_33410_56244391_1
ARTHROPODA_Candidate_35442_60258441_0
ARTHROPODA_TARA_MED_95_MAG_00510_000000005390.2.2
ARTHROPODA_Candidate_37398_64372971_5
ARTHROPODA_Candidate_41176_75003363_3
ARTHROPODA_Candidate_31536_49835327_4
ARTHROPODA_Candidate_49519_101681412_1
ARTHROPODA_TARA_MED_95_MAG_00510_000000005092.4.6
ARTHROPODA_Candidate_45688_91465453_0
ARTHROPODA_Candidate_45700_91465454_0
ARTHROPODA_TARA_ION_45_MAG_00181_000000003559.1.10
ARTHROPODA_Candidate_45013_88428352_5
ARTHROPODA_Candidate_34066_57378974_4
ARTHROPODA_Candidate_44995_88428351_5
ARTHROPODA_Candidate_40785_74795669_4
ARTHROPODA_Candidate_22947_32532270_3
ARTHROPODA_Candidate_40925_74823540_3
ARTHROPODA_TARA_PON_109_MAG_00256_000000006419.1.3
ARTHROPODA_TARA_PSE_93_MAG_00238_000000017842.5.7
ARTHROPODA_TARA_ION_45_MAG_00189_000000000364.1.2
ARTHROPODA_Candidate_39795_71618450_0
ARTHROPODA_Candidate_23021_32565311_5
ARTHROPODA_Candidate_26270_41240284_2
ARTHROPODA_Candidate_50294_102322798_0
ARTHROPODA_Candidate_52462_104122468_2
ARTHROPODA_Candidate_7393_16868261_0
ARTHROPODA_TARA_MED_95_MAG_00510_000000005092.4.14
ARTHROPODA_Candidate_7621_17508494_1
ARTHROPODA_TARA_PON_109_MAG_00256_000000003899.1.15
ARTHROPODA_Candidate_33978_57318904_5
ARTHROPODA_TARA_RED_19_MAG_00050_000000000945.1.3
ARTHROPODA_Candidate_50246_102286924_0
ARTHROPODA_Candidate_8426_20607179_0
ARTHROPODA_TARA_PSE_93_MAG_00238_000000010789.1.7
ARTHROPODA_Candidate_34614_58399570_3
ARTHROPODA_Candidate_8728_21887074_2
ARTHROPODA_Candidate_22892_32506798_2
ARTHROPODA_Candidate_48957_99461646_2
ARTHROPODA_Candidate_43554_81624014_4
ARTHROPODA_TARA_PSW_86_MAG_00298_000000022893.1.8
ARTHROPODA_Candidate_7370_16844324_5
ARTHROPODA_Candidate_23077_32584490_2
ARTHROPODA_Candidate_33280_55642477_5
ARTHROPODA_TARA_MED_95_MAG_00510_000000005285.1.3
ARTHROPODA_Candidate_11347_28317965_2
ARTHROPODA_Candidate_25279_38681292_2
ARTHROPODA_Candidate_10432_26996325_1
ARTHROPODA_Candidate_11316_28317964_0
ARTHROPODA_Candidate_35374_60202010_1
ARTHROPODA_Candidate_41101_74996725_5
ARTHROPODA_Candidate_6921_16658912_2
ARTHROPODA_TARA_MED_95_MAG_00510_000000004001.4.1
ARTHROPODA_Candidate_26835_41818864_3
ARTHROPODA_Candidate_26984_42075889_2
ARTHROPODA_Candidate_25808_39920838_2
ARTHROPODA_Candidate_25046_37819170_0
ARTHROPODA_Candidate_26042_40999460_1
ARTHROPODA_Candidate_25326_38697433_0
ARTHROPODA_Candidate_44484_84747450_3
ARTHROPODA_Candidate_41223_75024548_1
ARTHROPODA_Candidate_22834_32499205_1
ARTHROPODA_Candidate_24562_35634838_4
ARTHROPODA_Candidate_41196_75005668_1
ARTHROPODA_Candidate_37519_64423872_4
ARTHROPODA_Candidate_37541_64474946_0
ARTHROPODA_Candidate_37032_64114682_1
ARTHROPODA_Candidate_3837_8375226_0
ARTHROPODA_Candidate_2843_6492278_5
ARTHROPODA_Candidate_52093_104062801_0
ARTHROPODA_Candidate_38815_67062191_4
ARTHROPODA_Candidate_39845_71693492_5
ARTHROPODA_Candidate_1184_2432859_4
ARTHROPODA_Candidate_24816_36801033_0
ARTHROPODA_Candidate_25430_39060613_5
ARTHROPODA_Candidate_8960_22158440_3
ARTHROPODA_Candidate_9141_22621240_4
ARTHROPODA_Candidate_29682_46543665_5
ARTHROPODA_Candidate_29921_47912675_0
ARTHROPODA_Candidate_29555_46013832_0
ARTHROPODA_Candidate_31589_49885194_5
ARTHROPODA_Candidate_11405_28392370_0
ARTHROPODA_Candidate_33350_55689599_5
ARTHROPODA_Candidate_6377_14618671_1
ARTHROPODA_Candidate_31553_49850094_2
ARTHROPODA_Candidate_8581_21080977_3
ARTHROPODA_Candidate_10892_28053723_0
ARTHROPODA_Candidate_46819_94921838_5
ARTHROPODA_Candidate_46851_94921840_5
ARTHROPODA_Candidate_46837_94921839_5
ARTHROPODA_Candidate_30639_48448844_3
ARTHROPODA_Candidate_40138_72452195_5
ARTHROPODA_Candidate_32473_51529579_0
ARTHROPODA_Candidate_32477_51529579_1
ARTHROPODA_Candidate_67793_146538457_3
ARTHROPODA_TARA_MED_95_MAG_00482_000000007432.2.3
ARTHROPODA_Candidate_28940_44603032_5
ARTHROPODA_Candidate_67358_146419014_3
ARTHROPODA_Candidate_58444_120124903_4
ARTHROPODA_Candidate_62227_135539139_3
ARTHROPODA_Candidate_66186_141425113_5
ARTHROPODA_Candidate_40080_72287183_4
ARTHROPODA_Candidate_40087_72287184_4
ARTHROPODA_Candidate_29469_45872804_1
ARTHROPODA_Candidate_29928_47915252_3
ARTHROPODA_Candidate_38301_66465323_3
ARTHROPODA_Candidate_36425_62859718_0
ARTHROPODA_Candidate_40964_74843326_3
ARTHROPODA_TARA_PSE_93_MAG_00202_000000005208.1.1
ARTHROPODA_Candidate_38080_66098687_0
ARTHROPODA_Candidate_9217_22926830_1
ARTHROPODA_Candidate_39176_68728926_0
ARTHROPODA_TARA_IOS_50_MAG_00143_000000007556.4.1
ARTHROPODA_TARA_ION_45_MAG_00159_000000008594.1.5
ARTHROPODA_TARA_RED_19_MAG_00044_000000001521.1.7
ARTHROPODA_TARA_IOS_50_MAG_00132_000000000939.2.2
ARTHROPODA_Candidate_4061_10129650_2
ARTHROPODA_TARA_PSW_86_MAG_00304_000000007423.11.24
ARTHROPODA_TARA_PSW_86_MAG_00304_000000007423.11.8
ARTHROPODA_Candidate_4062_10130440_0
ARTHROPODA_TARA_IOS_50_MAG_00132_000000001057.1.1
ARTHROPODA_TARA_PSW_86_MAG_00304_000000022854.2.1
ARTHROPODA_TARA_PSW_86_MAG_00304_000000015438.6.1
ARTHROPODA_TARA_PSW_86_MAG_00304_000000010299.7.1
ARTHROPODA_TARA_PSW_86_MAG_00304_000000016631.1.1
ARTHROPODA_TARA_PSW_86_MAG_00304_000000005846.2.1
ARTHROPODA_TARA_IOS_50_MAG_00132_000000008293.1.3
ARTHROPODA_Candidate_25597_39276039_0
ARTHROPODA_TARA_AOS_82_MAG_00188_000000004762.1.4
ARTHROPODA_Candidate_38065_66604959_5
ARTHROPODA_Candidate_38455_66081116_2
ARTHROPODA_TARA_PSE_93_MAG_00268_000000006754.1.4
ARTHROPODA_Candidate_24160_35351625_3
ARTHROPODA_Candidate_30668_48692370_1
ARTHROPODA_Candidate_23339_33081170_1
ARTHROPODA_Candidate_24631_35910294_2
ARTHROPODA_TARA_MED_95_MAG_00441_000000008716.1.2
ARTHROPODA_Candidate_44638_87606623_0
ARTHROPODA_TARA_RED_19_MAG_00051_000000006595.1.3
ARTHROPODA_Candidate_50439_102423834_3
ARTHROPODA_Candidate_53810_106979864_2
ARTHROPODA_Candidate_26385_41674115_0
ARTHROPODA_Candidate_31578_49883798_3
ARTHROPODA_TARA_MED_95_MAG_00510_000000011610.2.2
ARTHROPODA_TARA_PON_109_MAG_00256_000000003394.7.2
ARTHROPODA_Candidate_40476_73193628_1
ARTHROPODA_Candidate_40491_73193629_1
ARTHROPODA_Candidate_5394_13259289_3
ARTHROPODA_TARA_PON_109_MAG_00275_000000000198.1.1
ARTHROPODA_Candidate_2837_6471634_2
ARTHROPODA_TARA_ION_45_MAG_00191_000000003472.1.1
ARTHROPODA_Candidate_11824_29640268_0
ARTHROPODA_TARA_MED_95_MAG_00414_000000001318.2.4
ARTHROPODA_TARA_MED_95_MAG_00414_000000005734.3.1
ARTHROPODA_TARA_PON_109_MAG_00275_000000008634.7.2
ARTHROPODA_Candidate_22472_30900267_0
ARTHROPODA_Candidate_22257_30620518_2
ARTHROPODA_Candidate_5163_13036575_1
ARTHROPODA_Candidate_7389_16867731_1
ARTHROPODA_TARA_IOS_50_MAG_00143_000000003295.2.3
ARTHROPODA_TARA_ARC_108_MAG_00282_000000000590.1.2
ARTHROPODA_TARA_MED_95_MAG_00482_000000000708.1.5
ARTHROPODA_Candidate_24398_35562692_1
ARTHROPODA_Candidate_47313_95068947_3
ARTHROPODA_Candidate_47328_95072131_4
ARTHROPODA_Candidate_7365_16814571_1
ARTHROPODA_TARA_RED_19_MAG_00059_000000015780.1.1
ARTHROPODA_TARA_ARC_108_MAG_00282_000000003921.4.1
ARTHROPODA_TARA_MED_95_MAG_00482_000000007687.2.2
ARTHROPODA_TARA_PSE_93_MAG_00250_000000000485.3.1
ARTHROPODA_TARA_AOS_82_MAG_00174_000000007333.3.1
ARTHROPODA_Candidate_68143_146867916_5
ARTHROPODA_TARA_AON_82_MAG_00353_000000004733.1.3
ARTHROPODA_Candidate_68016_146570117_3

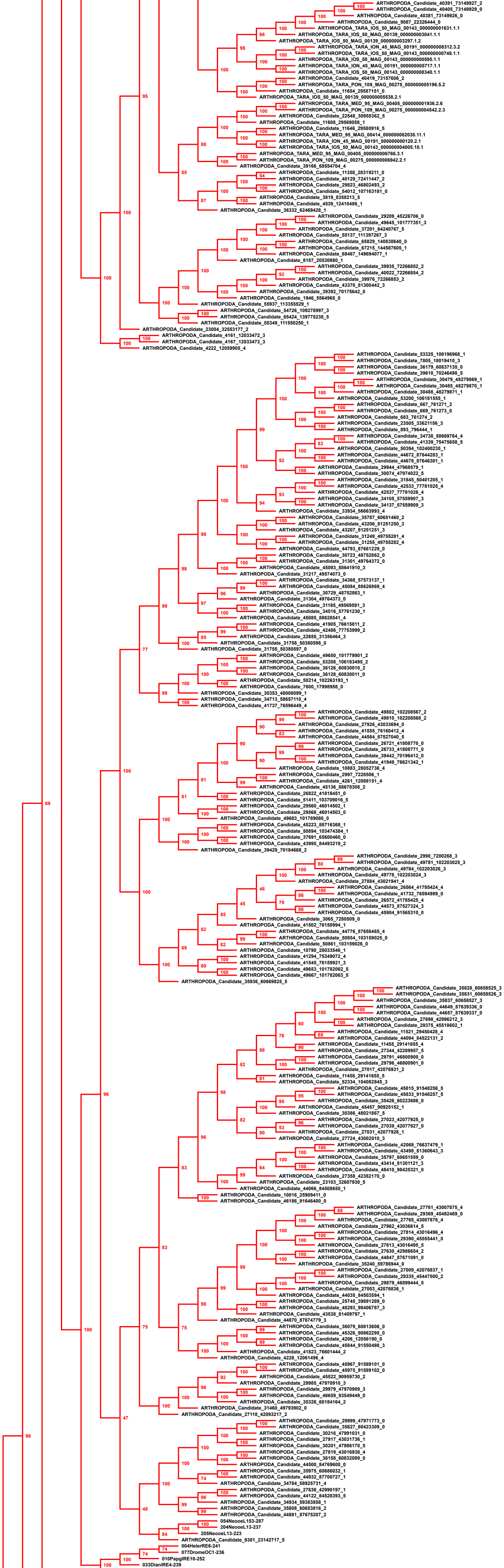

ARTHROPODA_Candidate_40391_73149927_2
ARTHROPODA_Candidate_40405_73149929_0
ARTHROPODA_Candidate_40381_73149926_0
ARTHROPODA_Candidate_9087_22326444_0
ARTHROPODA_TARA_IOS_50_MAG_00143_000000001631.1.1
ARTHROPODA_TARA_IOS_50_MAG_00139_000000003041.1.1
ARTHROPODA_TARA_IOS_50_MAG_00139_000000003297.1.2
ARTHROPODA_TARA_ION_45_MAG_00191_000000008312.3.2
ARTHROPODA_TARA_IOS_50_MAG_00143_000000000748.1.1
ARTHROPODA_TARA_IOS_50_MAG_00143_000000000595.1.1
ARTHROPODA_TARA_ION_45_MAG_00191_000000005717.1.1
ARTHROPODA_TARA_IOS_50_MAG_00143_000000008340.1.1
ARTHROPODA_Candidate_40419_73157606_2
ARTHROPODA_TARA_PON_109_MAG_00275_000000005196.5.2
ARTHROPODA_Candidate_11654_29587151_0
ARTHROPODA_TARA_IOS_50_MAG_00139_000000005538.2.1
ARTHROPODA_TARA_MED_95_MAG_00405_000000001936.2.6
ARTHROPODA_TARA_PON_109_MAG_00275_000000004542.2.3
ARTHROPODA_Candidate_22548_30955362_5
ARTHROPODA_Candidate_11608_29569058_1
ARTHROPODA_Candidate_11646_29580916_5
ARTHROPODA_TARA_MED_95_MAG_00414_000000002038.11.1
ARTHROPODA_TARA_ION_45_MAG_00191_000000000120.2.1
ARTHROPODA_TARA_IOS_50_MAG_00143_000000004005.10.1
ARTHROPODA_TARA_MED_95_MAG_00405_000000006766.3.1
ARTHROPODA_TARA_PON_109_MAG_00275_000000006942.2.1
ARTHROPODA_Candidate_39166_68554704_4
ARTHROPODA_Candidate_11358_28319211_0
ARTHROPODA_Candidate_40129_72411447_2
ARTHROPODA_Candidate_29823_46802493_2
ARTHROPODA_Candidate_54012_107163181_0
ARTHROPODA_Candidate_3819_8358213_5
ARTHROPODA_Candidate_4539_12410486_1
ARTHROPODA_Candidate_36332_62469428_1
ARTHROPODA_Candidate_29209_45226706_0
ARTHROPODA_Candidate_49645_101777351_3
ARTHROPODA_Candidate_37201_64240767_5
ARTHROPODA_Candidate_55137_111397267_3
ARTHROPODA_Candidate_65829_140838640_0
ARTHROPODA_Candidate_67215_144587605_1
ARTHROPODA_Candidate_68467_149694077_1
ARTHROPODA_Candidate_8187_20530880_1
ARTHROPODA_Candidate_39935_72266852_2
ARTHROPODA_Candidate_40022_72266854_2
ARTHROPODA_Candidate_39976_72266853_2
ARTHROPODA_Candidate_43370_81300442_3
ARTHROPODA_Candidate_39392_70175642_0
ARTHROPODA_Candidate_1946_5564965_0
ARTHROPODA_Candidate_55937_113355529_1
ARTHROPODA_Candidate_54726_109278997_3
ARTHROPODA_Candidate_65424_139775238_5
ARTHROPODA_Candidate_55349_111550250_1
ARTHROPODA_Candidate_23004_32553177_2
ARTHROPODA_Candidate_4161_12033472_3
ARTHROPODA_Candidate_4167_12033473_3
ARTHROPODA_Candidate_4222_12059905_4
ARTHROPODA_Candidate_53325_106196968_1
ARTHROPODA_Candidate_7805_18019410_3
ARTHROPODA_Candidate_36179_60837135_0
ARTHROPODA_Candidate_39618_70246498_5
ARTHROPODA_Candidate_30479_48279869_1
ARTHROPODA_Candidate_30485_48279870_1
ARTHROPODA_Candidate_30488_48279871_1
ARTHROPODA_Candidate_53200_106181555_1
ARTHROPODA_Candidate_667_761271_2
ARTHROPODA_Candidate_669_761273_0
ARTHROPODA_Candidate_683_761274_2
ARTHROPODA_Candidate_23505_33621156_3
ARTHROPODA_Candidate_893_796444_1
ARTHROPODA_Candidate_34738_58669764_4
ARTHROPODA_Candidate_41339_75475658_5
ARTHROPODA_Candidate_50394_102400235_1
ARTHROPODA_Candidate_44672_87644283_1
ARTHROPODA_Candidate_44678_87646301_1
ARTHROPODA_Candidate_29944_47968579_1
ARTHROPODA_Candidate_30074_47974022_5
ARTHROPODA_Candidate_31845_50401205_1
ARTHROPODA_Candidate_42533_77781025_4
ARTHROPODA_Candidate_42537_77781026_4
ARTHROPODA_Candidate_34105_57559907_3
ARTHROPODA_Candidate_34137_57559909_3
ARTHROPODA_Candidate_33934_56663993_4
ARTHROPODA_Candidate_35787_60651460_2
ARTHROPODA_Candidate_43206_81251250_3
ARTHROPODA_Candidate_43207_81251251_3
ARTHROPODA_Candidate_31249_49755281_4
ARTHROPODA_Candidate_31255_49755282_4
ARTHROPODA_Candidate_44793_87661229_0
ARTHROPODA_Candidate_30723_48752862_0
ARTHROPODA_Candidate_31301_49764372_0
ARTHROPODA_Candidate_45093_88641910_3
ARTHROPODA_Candidate_31217_49574073_0
ARTHROPODA_Candidate_34368_57573137_1
ARTHROPODA_Candidate_45084_88626869_4
ARTHROPODA_Candidate_30729_48752863_1
ARTHROPODA_Candidate_31304_49764373_0
ARTHROPODA_Candidate_31185_49569591_3
ARTHROPODA_Candidate_34516_57761230_1
ARTHROPODA_Candidate_45085_88628541_4
ARTHROPODA_Candidate_41905_76615811_2
ARTHROPODA_Candidate_42486_77753999_2
ARTHROPODA_Candidate_22655_31356464_3
ARTHROPODA_Candidate_31758_50380598_0
ARTHROPODA_Candidate_31755_50380597_0
ARTHROPODA_Candidate_49650_101779901_2
ARTHROPODA_Candidate_53208_106183495_2
ARTHROPODA_Candidate_36126_60830010_2
ARTHROPODA_Candidate_36128_60830011_0
ARTHROPODA_Candidate_50214_102263193_1
ARTHROPODA_Candidate_7800_17998958_0
ARTHROPODA_Candidate_30353_48008099_1
ARTHROPODA_Candidate_34713_58657110_4
ARTHROPODA_Candidate_41737_76596449_4
ARTHROPODA_Candidate_49802_102208567_2
ARTHROPODA_Candidate_49810_102208568_2
ARTHROPODA_Candidate_27926_43033694_0
ARTHROPODA_Candidate_41555_76160412_4
ARTHROPODA_Candidate_44564_87527040_5
ARTHROPODA_Candidate_26721_41808770_0
ARTHROPODA_Candidate_26733_41808771_0
ARTHROPODA_Candidate_39442_70196412_0
ARTHROPODA_Candidate_41949_76621342_1
ARTHROPODA_Candidate_10883_28052736_4
ARTHROPODA_Candidate_2997_7228506_1
ARTHROPODA_Candidate_4261_12088151_4
ARTHROPODA_Candidate_45136_88678308_2
ARTHROPODA_Candidate_26822_41818451_0
ARTHROPODA_Candidate_51411_103709016_5
ARTHROPODA_Candidate_29560_46014502_1
ARTHROPODA_Candidate_29568_46014503_0
ARTHROPODA_Candidate_49683_101789080_0
ARTHROPODA_Candidate_45223_88716368_1
ARTHROPODA_Candidate_50894_103474384_1
ARTHROPODA_Candidate_37691_65600460_0
ARTHROPODA_Candidate_43995_84493219_2
ARTHROPODA_Candidate_39429_70184668_2
ARTHROPODA_Candidate_2990_7200268_3
ARTHROPODA_Candidate_49781_102203025_3
ARTHROPODA_Candidate_49784_102203026_3
ARTHROPODA_Candidate_49778_102203024_3
ARTHROPODA_Candidate_27884_43021941_4
ARTHROPODA_Candidate_26564_41785424_4
ARTHROPODA_Candidate_41732_76594989_0
ARTHROPODA_Candidate_26572_41785425_4
ARTHROPODA_Candidate_44573_87527324_3
ARTHROPODA_Candidate_45904_91565310_0
ARTHROPODA_Candidate_3065_7280509_0
ARTHROPODA_Candidate_41502_76150994_1
ARTHROPODA_Candidate_44776_87658465_4
ARTHROPODA_Candidate_50854_103159025_0
ARTHROPODA_Candidate_50861_103159026_0
ARTHROPODA_Candidate_10790_28033546_1
ARTHROPODA_Candidate_41294_75349072_4
ARTHROPODA_Candidate_41545_76159921_3
ARTHROPODA_Candidate_49653_101782062_5
ARTHROPODA_Candidate_49667_101782063_5
ARTHROPODA_Candidate_35936_60669825_5
ARTHROPODA_Candidate_35828_60658525_3
ARTHROPODA_Candidate_35831_60658526_3
ARTHROPODA_Candidate_35837_60658527_3
ARTHROPODA_Candidate_44649_87639336_0
ARTHROPODA_Candidate_44657_87639337_0
ARTHROPODA_Candidate_27698_42996212_3
ARTHROPODA_Candidate_29375_45518602_1
ARTHROPODA_Candidate_11521_29450428_4
ARTHROPODA_Candidate_44094_84522131_2
ARTHROPODA_Candidate_11455_29141655_4
ARTHROPODA_Candidate_27344_42289957_5
ARTHROPODA_Candidate_29791_46800900_0
ARTHROPODA_Candidate_29796_46800901_0
ARTHROPODA_Candidate_27017_42076931_2
ARTHROPODA_Candidate_11458_29141655_5
ARTHROPODA_Candidate_52334_104082845_3
ARTHROPODA_Candidate_45815_91548256_5
ARTHROPODA_Candidate_45833_91548257_5
ARTHROPODA_Candidate_35426_60223686_0
ARTHROPODA_Candidate_45457_90925152_1
ARTHROPODA_Candidate_30386_48021867_5
ARTHROPODA_Candidate_27023_42077925_0
ARTHROPODA_Candidate_27039_42077927_0
ARTHROPODA_Candidate_27031_42077926_1
ARTHROPODA_Candidate_27724_43002010_3
ARTHROPODA_Candidate_42068_76637479_1
ARTHROPODA_Candidate_43498_81360643_3
ARTHROPODA_Candidate_35797_60651559_0
ARTHROPODA_Candidate_43414_81301121_3
ARTHROPODA_Candidate_48410_98425321_0
ARTHROPODA_Candidate_27358_42382170_0
ARTHROPODA_Candidate_23103_32607930_5
ARTHROPODA_Candidate_44066_84508650_1
ARTHROPODA_Candidate_10016_25909411_0
ARTHROPODA_Candidate_46186_91646400_0
ARTHROPODA_Candidate_27761_43007875_4
ARTHROPODA_Candidate_29369_45492469_0
ARTHROPODA_Candidate_27765_43007876_4
ARTHROPODA_Candidate_27962_43036614_5
ARTHROPODA_Candidate_27814_43016496_4
ARTHROPODA_Candidate_29390_45555441_0
ARTHROPODA_Candidate_27813_43016495_5
ARTHROPODA_Candidate_27630_42986654_2
ARTHROPODA_Candidate_44847_87671091_0
ARTHROPODA_Candidate_35240_59786944_0
ARTHROPODA_Candidate_27009_42076837_1
ARTHROPODA_Candidate_29335_45447500_2
ARTHROPODA_Candidate_29879_46899444_5
ARTHROPODA_Candidate_27003_42076836_1
ARTHROPODA_Candidate_44035_84503594_1
ARTHROPODA_Candidate_25745_39891289_0
ARTHROPODA_Candidate_48293_98406787_3
ARTHROPODA_Candidate_43538_81409797_1
ARTHROPODA_Candidate_44870_87674779_3
ARTHROPODA_Candidate_36079_60813606_0
ARTHROPODA_Candidate_45326_90862295_0
ARTHROPODA_Candidate_4206_12056190_0
ARTHROPODA_Candidate_45844_91550498_3
ARTHROPODA_Candidate_41823_76601444_2
ARTHROPODA_Candidate_4228_12061496_4
ARTHROPODA_Candidate_45967_91589101_0
ARTHROPODA_Candidate_45975_91589102_0
ARTHROPODA_Candidate_45522_90959730_2
ARTHROPODA_Candidate_29985_47970910_3
ARTHROPODA_Candidate_29979_47970909_3
ARTHROPODA_Candidate_46659_93549449_0
ARTHROPODA_Candidate_35326_60184164_2
ARTHROPODA_Candidate_31460_49793902_0
ARTHROPODA_Candidate_27118_42093217_2
ARTHROPODA_Candidate_29999_47971773_0
ARTHROPODA_Candidate_35627_60423309_0
ARTHROPODA_Candidate_30216_47991031_0
ARTHROPODA_Candidate_27917_43031736_1
ARTHROPODA_Candidate_30201_47986170_5
ARTHROPODA_Candidate_27819_43016938_4
ARTHROPODA_Candidate_36158_60832089_0
ARTHROPODA_Candidate_44500_84769600_0
ARTHROPODA_Candidate_35975_60686032_1
ARTHROPODA_Candidate_44932_87700727_1
ARTHROPODA_Candidate_34784_58925731_4
ARTHROPODA_Candidate_27636_42990197_1
ARTHROPODA_Candidate_44122_84528393_5
ARTHROPODA_Candidate_34934_59383858_1
ARTHROPODA_Candidate_35808_60653816_2
ARTHROPODA_Candidate_44881_87675207_2
054NeooeL153-287
204NeooeL13-237
205NeooeL13-223
ARTHROPODA_Candidate_9301_23142717_5
004HelerRE6-241
077DromeOC1-236
010PapglRE18-252
033DianiRE4-239

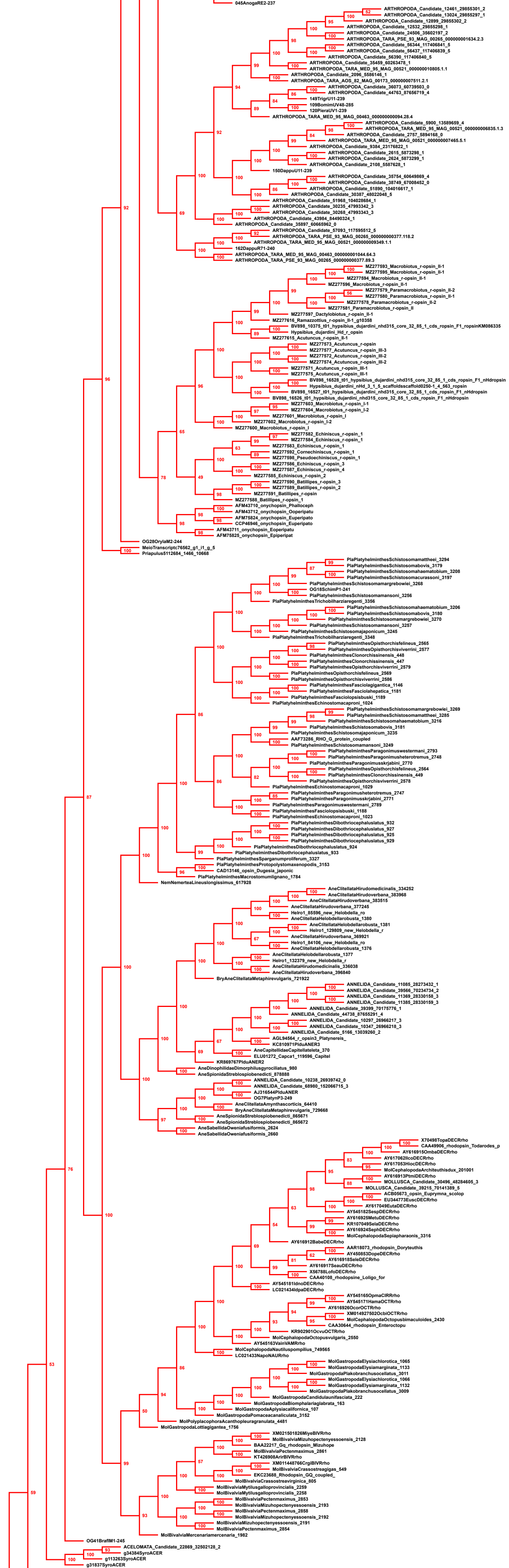

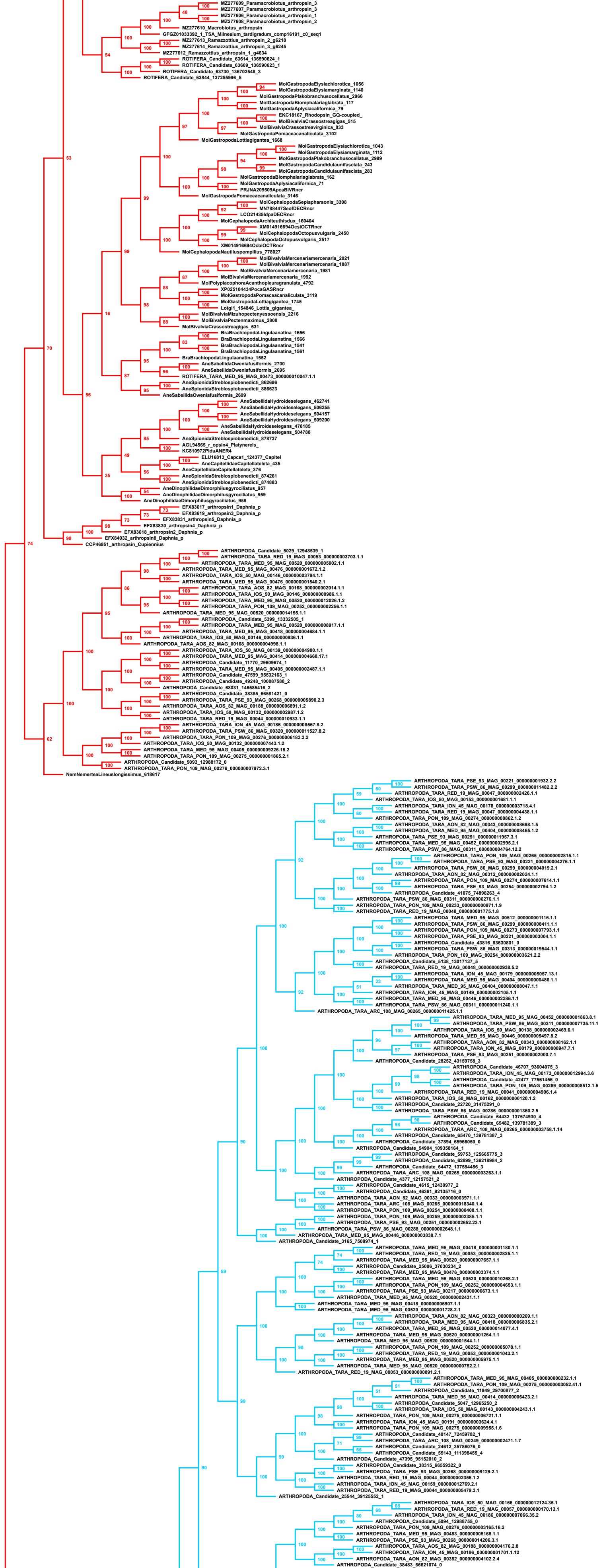
MZ277609_Paramacrobiotus_arthropsin_3
MZ277607_Paramacrobiotus_arthropsin_3
MZ277606_Paramacrobiotus_arthropsin_1
MZ277608_Paramacrobiotus_arthropsin_2
MZ277610_Macrobiotus_arthropsin
GFGZ01033392_1_TSA_Milnesium_tardigradum_comp16191_c0_seq1
MZ277613_Ramazzottius_arthropsin_2_g6218
MZ277614_Ramazzottius_arthropsin_3_g6245
MZ277612_Ramazzottius_arthropsin_1_g4634
ROTIFERA_Candidate_63614_136590624_1
ROTIFERA_Candidate_63609_136590623_1
ROTIFERA_Candidate_63730_136702548_3
ROTIFERA_Candidate_63844_137255996_5
MolGastropodaElysiachlorotica_1056
MolGastropodaElysiamarginata_1140
MolGastropodaPlakobranchusocellatus_2966
MolGastropodaBiomphalariaglabrata_117
MolGastropodaAplysiacalifornica_79
EKC18167_Rhodopsin_GQ-coupled_
MolGastropodaCrassostreagigas_515
MolBivalviaCrassostreavirginica_833
MolGastropodaPomaceacanaliculata_3102
MolGastropodaLottiagigantea_1668
MolGastropodaElysiachlorotica_1043
MolGastropodaElysiamarginata_1112
MolGastropodaPlakobranchusocellatus_2999
MolGastropodaCandidulaunifasciata_243
MolGastropodaCandidulaunifasciata_283
MolGastropodaBiomphalariaglabrata_162
MolGastropodaAplysiacalifornica_71
PRJNA209509ApcaBIVRncr
MolGastropodaPomaceacanaliculata_3146
MolCephalopodaSepiapharaonis_3308
MN788447SeofDECRncr
LCO21435ldpaDECRncr
MolCephalopodaArchiteuthisdux_160404
XM014916694OcsiOCTRncr
MolCephalopodaOctopusvulgaris_2450
MolCephalopodaOctopusvulgaris_2517
XM014916694OcbiOCTRncr
MolCephalopodaNautiluspompilius_778027
MolBivalviaMercenariamercenaria_2021
MolBivalviaMercenariamercenaria_1887
MolBivalviaMercenariamercenaria_1981
MolBivalviaMercenariamercenaria_1992
MolPolyplacophoraAcanthopleuragranulata_4792
XP025104434PocaGASRncr
MolGastropodaPomaceacanaliculata_3119
MolGastropodaLottiagigantea_1745
Lotgi1_154846_Lottia_gigantea_
MolBivalviaMizuhopectenyessoensis_2216
MolBivalviaPectenmaximus_2808
MolBivalviaCrassostreagigas_531
BraBrachiopodaLingulaanatina_1656
BraBrachiopodaLingulaanatina_1566
BraBrachiopodaLingulaanatina_1541
BraBrachiopodaLingulaanatina_1561
BraBrachiopodaLingulaanatina_1552
AneSabellidaOweniafusiformis_2700
AneSabellidaOweniafusiformis_2695
ROTIFERA_TARA_MED_95_MAG_00473_000000010047.1.1
AneSpionidaStreblospiobenedicti_862696
AneSpionidaStreblospiobenedicti_886623
AneSabellidaOweniafusiformis_2699
AneSabellidaHydroideselegans_462741
AneSabellidaHydroideselegans_506255
AneSabellidaHydroideselegans_504157
AneSabellidaHydroideselegans_509200
AneSabellidaHydroideselegans_478185
AneSabellidaHydroideselegans_504788
AneSpionidaStreblospiobenedicti_878737
AGL94565_r_opsin4_Platynereis_
KC810972PlduANER4
ELU16813_Capca1_124377_Capitel
AneCapitellidaeCapitellateleta_435
AneCapitellidaeCapitellateleta_376
AneSpionidaStreblospiobenedicti_874261
AneSpionidaStreblospiobenedicti_874883
AneDinophilidaeDimorphilusgyrociliatus_957
AneDinophilidaeDimorphilusgyrociliatus_959
AneDinophilidaeDimorphilusgyrociliatus_958
EFX83617_arthropsin1_Daphnia_p
EFX83619_arthropsin3_Daphnia_p
EFX83831_arthropsin5_Daphnia_p
EFX83830_arthropsin4_Daphnia_p
EFX83618_arthropsin2_Daphnia_p
EFX84032_arthropsin8_Daphnia_p
CCP46951_arthropsin_Cupiennius
ARTHROPODA_Candidate_5029_12948539_1
ARTHROPODA_TARA_RED_19_MAG_00053_000000003703.1.1
ARTHROPODA_TARA_MED_95_MAG_00520_000000005002.1.1
ARTHROPODA_TARA_MED_95_MAG_00476_000000001672.1.2
ARTHROPODA_TARA_IOS_50_MAG_00146_000000003794.1.1
ARTHROPODA_TARA_MED_95_MAG_00476_000000001540.2.1
ARTHROPODA_TARA_AOS_82_MAG_00168_000000002014.1.1
ARTHROPODA_TARA_IOS_50_MAG_00146_000000000986.1.1
ARTHROPODA_TARA_MED_95_MAG_00520_000000012026.1.2
ARTHROPODA_TARA_PON_109_MAG_00252_000000002256.1.1
ARTHROPODA_TARA_MED_95_MAG_00520_000000014155.1.1
ARTHROPODA_Candidate_5399_13332505_1
ARTHROPODA_TARA_MED_95_MAG_00520_000000008917.1.1
ARTHROPODA_TARA_MED_95_MAG_00418_000000004684.1.1
ARTHROPODA_TARA_IOS_50_MAG_00146_000000000936.1.1
ARTHROPODA_TARA_AOS_82_MAG_00168_000000004998.1.1
ARTHROPODA_TARA_IOS_50_MAG_00139_000000004980.1.1
ARTHROPODA_TARA_MED_95_MAG_00414_000000004668.17.1
ARTHROPODA_Candidate_11770_29609674_1
ARTHROPODA_TARA_MED_95_MAG_00405_000000002487.1.1
ARTHROPODA_Candidate_47599_95532163_1
ARTHROPODA_Candidate_49248_100087588_2
ARTHROPODA_Candidate_68031_146555416_2
ARTHROPODA_Candidate_38385_66581421_0
ARTHROPODA_TARA_PSE_93_MAG_00268_000000005890.2.3
ARTHROPODA_TARA_AOS_82_MAG_00188_000000006891.1.2
ARTHROPODA_TARA_IOS_50_MAG_00132_000000002987.1.2
ARTHROPODA_TARA_RED_19_MAG_00044_000000010933.1.1
ARTHROPODA_TARA_ION_45_MAG_00186_000000008567.8.2
ARTHROPODA_TARA_PSW_86_MAG_00320_000000011527.8.2
ARTHROPODA_TARA_PON_109_MAG_00276_000000006183.3.2
ARTHROPODA_TARA_IOS_50_MAG_00132_000000007443.1.2
ARTHROPODA_TARA_MED_95_MAG_00405_000000009226.15.2
ARTHROPODA_TARA_PON_109_MAG_00275_000000001865.2.1
ARTHROPODA_Candidate_5093_12988172_0
ARTHROPODA_TARA_PON_109_MAG_00276_000000007972.3.1
NemNemerteaLineuslongissimus_618617
ARTHROPODA_TARA_PSE_93_MAG_00221_000000001932.2.2
ARTHROPODA_TARA_PSW_86_MAG_00299_000000011482.2.2
ARTHROPODA_TARA_RED_19_MAG_00047_000000002426.1.1
ARTHROPODA_TARA_IOS_50_MAG_00153_000000001681.1.1
ARTHROPODA_TARA_ION_45_MAG_00178_000000003718.4.1
ARTHROPODA_TARA_RED_19_MAG_00047_000000004438.1.1
ARTHROPODA_TARA_PON_109_MAG_00274_000000008862.1.2
ARTHROPODA_TARA_AON_82_MAG_00343_000000008698.1.5
ARTHROPODA_TARA_MED_95_MAG_00404_000000008465.1.2
ARTHROPODA_TARA_PSE_93_MAG_00251_000000011957.3.1
ARTHROPODA_TARA_MED_95_MAG_00452_000000002995.2.1
ARTHROPODA_TARA_PSW_86_MAG_00311_000000004764.12.2
ARTHROPODA_TARA_PON_109_MAG_00265_000000002815.1.1
ARTHROPODA_TARA_PSE_93_MAG_00221_000000004276.1.1
ARTHROPODA_TARA_PSW_86_MAG_00299_000000004019.2.1
ARTHROPODA_TARA_AON_82_MAG_00312_000000002024.1.1
ARTHROPODA_TARA_PON_109_MAG_00274_000000007614.1.1
ARTHROPODA_TARA_PSE_93_MAG_00254_000000002794.1.2
ARTHROPODA_Candidate_41075_74898263_4
ARTHROPODA_TARA_PSW_86_MAG_00311_000000006276.1.1
ARTHROPODA_TARA_PON_109_MAG_00233_000000000971.1.9
ARTHROPODA_TARA_RED_19_MAG_00048_000000001775.1.8
ARTHROPODA_TARA_MED_95_MAG_00512_000000001116.1.1
ARTHROPODA_TARA_PSW_86_MAG_00299_000000008411.1.1
ARTHROPODA_TARA_PON_109_MAG_00273_000000007793.1.1
ARTHROPODA_TARA_PSE_93_MAG_00221_000000003004.1.1
ARTHROPODA_Candidate_43816_83630801_0
ARTHROPODA_TARA_PSW_86_MAG_00313_000000019544.1.1
ARTHROPODA_TARA_PON_109_MAG_00254_000000003621.2.2
ARTHROPODA_Candidate_5138_13017137_5
ARTHROPODA_TARA_RED_19_MAG_00048_000000002938.5.2
ARTHROPODA_TARA_ION_45_MAG_00179_000000005057.13.1
ARTHROPODA_TARA_MED_95_MAG_00404_000000000486.1.1
ARTHROPODA_TARA_MED_95_MAG_00404_000000008047.1.1
ARTHROPODA_TARA_ION_45_MAG_00149_000000002105.1.1
ARTHROPODA_TARA_MED_95_MAG_00446_000000002286.1.1
ARTHROPODA_TARA_PSW_86_MAG_00311_000000011240.1.1
ARTHROPODA_TARA_ARC_108_MAG_00265_000000011425.1.1
ARTHROPODA_TARA_MED_95_MAG_00452_000000001863.8.1
ARTHROPODA_TARA_PSW_86_MAG_00311_000000007735.11.1
ARTHROPODA_TARA_IOS_50_MAG_00138_000000002469.6.1
ARTHROPODA_TARA_MED_95_MAG_00446_000000005497.8.2
ARTHROPODA_TARA_AON_82_MAG_00343_000000008162.1.1
ARTHROPODA_TARA_ION_45_MAG_00179_000000008947.7.1
ARTHROPODA_TARA_PSE_93_MAG_00251_000000002000.7.1
ARTHROPODA_Candidate_28252_43159758_3
ARTHROPODA_Candidate_46707_93604075_3
ARTHROPODA_TARA_ION_45_MAG_00173_000000012994.3.6
ARTHROPODA_Candidate_42477_77561456_0
ARTHROPODA_TARA_PON_109_MAG_00269_000000008512.1.5
ARTHROPODA_TARA_RED_19_MAG_00041_000000004906.1.4
ARTHROPODA_TARA_IOS_50_MAG_00162_000000000120.1.2
ARTHROPODA_Candidate_22720_31475291_0
ARTHROPODA_TARA_PSW_86_MAG_00286_000000001360.2.5
ARTHROPODA_Candidate_64432_137574930_4
ARTHROPODA_Candidate_65482_139781389_3
ARTHROPODA_TARA_ARC_108_MAG_00265_000000003758.1.14
ARTHROPODA_Candidate_65470_139781387_3
ARTHROPODA_Candidate_37894_65966050_0
ARTHROPODA_Candidate_54904_109358164_1
ARTHROPODA_Candidate_59753_125665775_3
ARTHROPODA_Candidate_62899_136218984_2
ARTHROPODA_Candidate_64472_137584456_3
ARTHROPODA_TARA_ARC_108_MAG_00265_000000003263.1.1
ARTHROPODA_Candidate_4377_12157521_2
ARTHROPODA_Candidate_4615_12430977_2
ARTHROPODA_Candidate_46361_92135716_0
ARTHROPODA_TARA_AON_82_MAG_00333_000000003971.1.1
ARTHROPODA_TARA_ARC_108_MAG_00265_000000018340.1.4
ARTHROPODA_TARA_PON_109_MAG_00254_000000000408.1.1
ARTHROPODA_TARA_PON_109_MAG_00259_000000002385.1.1
ARTHROPODA_TARA_PSE_93_MAG_00251_000000002652.23.1
ARTHROPODA_TARA_PSW_86_MAG_00288_000000002648.1.1
ARTHROPODA_TARA_MED_95_MAG_00446_000000003838.7.1
ARTHROPODA_Candidate_3165_7508974_1
ARTHROPODA_TARA_MED_95_MAG_00418_000000001180.1.1
ARTHROPODA_TARA_RED_19_MAG_00053_000000002825.1.1
ARTHROPODA_TARA_MED_95_MAG_00520_000000007657.1.1
ARTHROPODA_Candidate_25006_37030234_2
ARTHROPODA_TARA_MED_95_MAG_00476_000000003374.1.1
ARTHROPODA_TARA_MED_95_MAG_00520_000000010268.2.1
ARTHROPODA_TARA_PON_109_MAG_00252_000000004653.1.1
ARTHROPODA_TARA_PSE_93_MAG_00217_000000006673.1.1
ARTHROPODA_TARA_MED_95_MAG_00520_000000002431.1.1
ARTHROPODA_TARA_MED_95_MAG_00418_000000006907.1.1
ARTHROPODA_TARA_MED_95_MAG_00520_000000001728.2.1
ARTHROPODA_TARA_AON_82_MAG_00323_000000000269.1.1
ARTHROPODA_TARA_MED_95_MAG_00418_000000006835.2.1
ARTHROPODA_TARA_MED_95_MAG_00520_000000014077.4.1
ARTHROPODA_TARA_MED_95_MAG_00520_000000001264.1.1
ARTHROPODA_TARA_MED_95_MAG_00520_000000001544.1.1
ARTHROPODA_TARA_PON_109_MAG_00252_000000005078.1.1
ARTHROPODA_TARA_RED_19_MAG_00053_000000001043.2.1
ARTHROPODA_TARA_MED_95_MAG_00520_000000005975.1.1
ARTHROPODA_TARA_MED_95_MAG_00520_000000000752.2.1
ARTHROPODA_TARA_RED_19_MAG_00053_000000000891.2.1
ARTHROPODA_TARA_MED_95_MAG_00405_000000000232.1.1
ARTHROPODA_TARA_PON_109_MAG_00275_000000003052.41.1
ARTHROPODA_Candidate_11949_29700877_2
ARTHROPODA_TARA_MED_95_MAG_00414_000000006423.2.1
ARTHROPODA_Candidate_5047_12965250_2
ARTHROPODA_TARA_IOS_50_MAG_00143_000000004243.1.1
ARTHROPODA_TARA_PON_109_MAG_00275_000000006721.1.1
ARTHROPODA_TARA_ION_45_MAG_00191_000000003624.4.1
ARTHROPODA_TARA_PON_109_MAG_00275_000000009955.1.6
ARTHROPODA_Candidate_40147_72459782_1
ARTHROPODA_TARA_ARC_108_MAG_00249_000000002471.1.7
ARTHROPODA_Candidate_24612_35786076_0
ARTHROPODA_Candidate_55143_111398455_4
ARTHROPODA_Candidate_47395_95152010_2
ARTHROPODA_Candidate_38315_66559322_0
ARTHROPODA_TARA_PSE_93_MAG_00268_000000009129.2.1
ARTHROPODA_TARA_RED_19_MAG_00044_000000002356.1.2
ARTHROPODA_TARA_ION_45_MAG_00159_000000012769.2.1
ARTHROPODA_TARA_RED_19_MAG_00044_000000005479.3.1
ARTHROPODA_Candidate_25544_39125552_1
ARTHROPODA_TARA_IOS_50_MAG_00166_000000012124.35.1
ARTHROPODA_TARA_RED_19_MAG_00057_000000000170.13.1
ARTHROPODA_TARA_ION_45_MAG_00186_000000007066.35.2
ARTHROPODA_Candidate_5094_12988755_0
ARTHROPODA_TARA_PON_109_MAG_00276_000000003165.16.2
ARTHROPODA_TARA_MED_95_MAG_00483_000000005168.1.1
ARTHROPODA_TARA_PSE_93_MAG_00268_000000014206.3.1
ARTHROPODA_TARA_AOS_82_MAG_00188_000000004176.2.8
ARTHROPODA_TARA_ION_45_MAG_00186_000000001701.1.12
ARTHROPODA_TARA_AON_82_MAG_00352_000000004102.2.4
ARTHROPODA_Candidate_38483_66621074_0

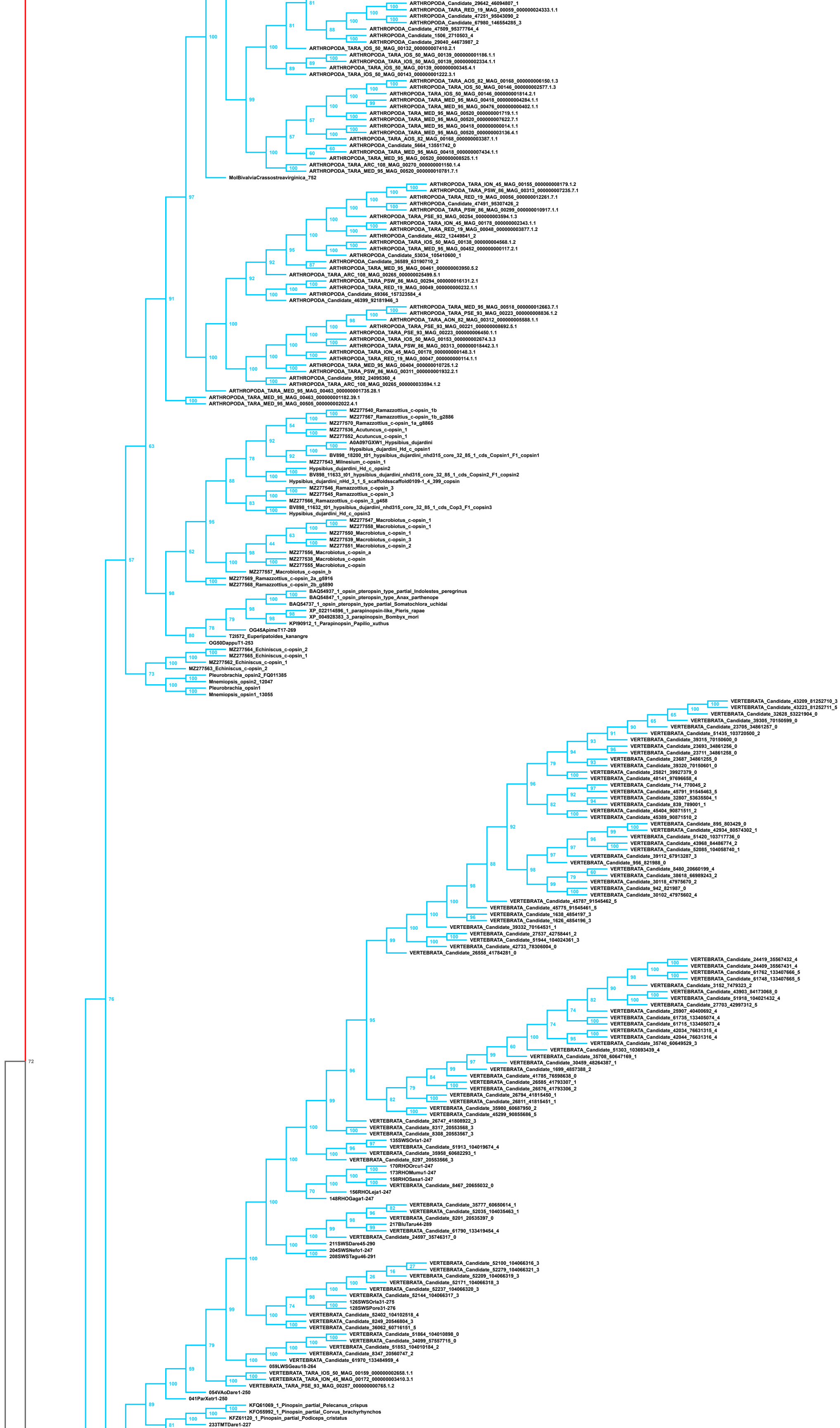

ARTHROPODA_Candidate_29642_46094807_1
ARTHROPODA_TARA_RED_19_MAG_00059_000000024333.1.1
ARTHROPODA_Candidate_47251_95043090_2
ARTHROPODA_Candidate_67980_146554285_3
ARTHROPODA_Candidate_47509_95377764_4
ARTHROPODA_Candidate_1506_2710503_4
ARTHROPODA_Candidate_29040_44673987_2
ARTHROPODA_TARA_IOS_50_MAG_00132_000000007410.2.1
ARTHROPODA_TARA_IOS_50_MAG_00139_000000001186.1.1
ARTHROPODA_TARA_IOS_50_MAG_00139_000000002334.1.1
ARTHROPODA_TARA_IOS_50_MAG_00139_000000000345.4.1
ARTHROPODA_TARA_IOS_50_MAG_00143_000000001222.3.1
ARTHROPODA_TARA_AOS_82_MAG_00168_000000006150.1.3
ARTHROPODA_TARA_IOS_50_MAG_00146_000000002577.1.3
ARTHROPODA_TARA_IOS_50_MAG_00146_000000001814.2.1
ARTHROPODA_TARA_MED_95_MAG_00418_000000004284.1.1
ARTHROPODA_TARA_MED_95_MAG_00476_000000000402.1.1
ARTHROPODA_TARA_MED_95_MAG_00520_000000001719.1.1
ARTHROPODA_TARA_MED_95_MAG_00520_000000007622.7.1
ARTHROPODA_TARA_MED_95_MAG_00418_000000000014.1.1
ARTHROPODA_TARA_MED_95_MAG_00520_000000003136.4.1
ARTHROPODA_TARA_AOS_82_MAG_00168_000000003387.1.1
ARTHROPODA_Candidate_5664_13551742_0
ARTHROPODA_TARA_MED_95_MAG_00418_000000007434.1.1
ARTHROPODA_TARA_MED_95_MAG_00520_000000008525.1.1
ARTHROPODA_TARA_ARC_108_MAG_00270_000000001150.1.4
ARTHROPODA_TARA_MED_95_MAG_00520_000000010781.7.1
MolBivalviaCrassostreavirginica_752
ARTHROPODA_TARA_ION_45_MAG_00155_000000008179.1.2
ARTHROPODA_TARA_PSW_86_MAG_00313_000000007235.7.1
ARTHROPODA_TARA_RED_19_MAG_00056_000000012261.7.1
ARTHROPODA_Candidate_47491_95307426_2
ARTHROPODA_TARA_PSW_86_MAG_00299_000000010917.1.1
ARTHROPODA_TARA_PSE_93_MAG_00254_000000003594.1.3
ARTHROPODA_TARA_ION_45_MAG_00178_000000002343.1.1
ARTHROPODA_TARA_RED_19_MAG_00048_000000003877.1.2
ARTHROPODA_Candidate_4622_12449841_2
ARTHROPODA_TARA_IOS_50_MAG_00138_000000004568.1.2
ARTHROPODA_TARA_MED_95_MAG_00452_000000000117.2.1
ARTHROPODA_Candidate_53034_105410600_1
ARTHROPODA_Candidate_36589_63190710_2
ARTHROPODA_TARA_MED_95_MAG_00461_000000003950.5.2
ARTHROPODA_TARA_ARC_108_MAG_00265_000000025499.5.1
ARTHROPODA_TARA_PSW_86_MAG_00294_000000016131.2.1
ARTHROPODA_TARA_RED_19_MAG_00049_000000000232.1.1
ARTHROPODA_Candidate_69366_157323584_4
ARTHROPODA_Candidate_46399_92181946_3
ARTHROPODA_TARA_MED_95_MAG_00518_000000012663.7.1
ARTHROPODA_TARA_PSE_93_MAG_00223_000000008836.1.2
ARTHROPODA_TARA_AON_82_MAG_00312_000000005588.1.1
ARTHROPODA_TARA_PSE_93_MAG_00221_000000008692.5.1
ARTHROPODA_TARA_PSE_93_MAG_00223_000000006450.1.1
ARTHROPODA_TARA_IOS_50_MAG_00153_000000002674.3.3
ARTHROPODA_TARA_PSW_86_MAG_00313_000000018442.3.1
ARTHROPODA_TARA_ION_45_MAG_00178_000000000148.3.1
ARTHROPODA_TARA_RED_19_MAG_00047_000000000114.1.1
ARTHROPODA_TARA_MED_95_MAG_00404_000000010725.1.2
ARTHROPODA_TARA_PSW_86_MAG_00311_000000001932.2.1
ARTHROPODA_Candidate_9592_24095360_4
ARTHROPODA_TARA_ARC_108_MAG_00265_000000033594.1.2
ARTHROPODA_TARA_MED_95_MAG_00463_000000001735.28.1
ARTHROPODA_TARA_MED_95_MAG_00463_000000001182.39.1
ARTHROPODA_TARA_MED_95_MAG_00505_000000002022.4.1
MZ277540_Ramazzottius_c-opsin_1b
MZ277567_Ramazzottius_c-opsin_1b_g2886
MZ277570_Ramazzottius_c-opsin_1a_g8865
MZ277536_Acutuncus_c-opsin_1
MZ277552_Acutuncus_c-opsin_1
A0A097GXW1_Hypsibius_dujardini
Hypsibius_dujardini_Hd_c_opsin1
BV898_18200_t01_hypsibius_dujardini_nhd315_core_32_85_1_cds_Copsin1_F1_copsin1
MZ277543_Milnesium_c-opsin_1
Hypsibius_dujardini_Hd_c_opsin2
BV898_11633_t01_hypsibius_dujardini_nhd315_core_32_85_1_cds_Copsin2_F1_copsin2
Hypsibius_dujardini_nHd_3_1_5_scaffoldsscaffold0109-1_4_399_copsin
MZ277546_Ramazzottius_c-opsin_3
MZ277545_Ramazzottius_c-opsin_3
MZ277566_Ramazzottius_c-opsin_3_g458
BV898_11632_t01_hypsibius_dujardini_nhd315_core_32_85_1_cds_Cop3_F1_copsin3
Hypsibius_dujardini_Hd_c_opsin3
MZ277547_Macrobiotus_c-opsin_1
MZ277558_Macrobiotus_c-opsin_1
MZ277550_Macrobiotus_c-opsin_1
MZ277539_Macrobiotus_c-opsin_3
MZ277551_Macrobiotus_c-opsin_2
MZ277556_Macrobiotus_c-opsin_a
MZ277538_Macrobiotus_c-opsin
MZ277555_Macrobiotus_c-opsin
MZ277557_Macrobiotus_c-opsin_b
MZ277569_Ramazzottius_c-opsin_2a_g5916
MZ277568_Ramazzottius_c-opsin_2b_g5890
BAQ54937_1_opsin_pteropsin_type_partial_Indolestes_peregrinus
BAQ54847_1_opsin_pteropsin_type_Anax_parthenope
BAQ54737_1_opsin_pteropsin_type_partial_Somatochlora_uchidai
XP_022114596_1_parapinopsin-like_Pieris_rapae
XP_004928383_3_parapinopsin_Bombyx_mori
KPI90912_1_Parapinopsin_Papilio_xuthus
OG45ApimeT17-269
T2I572_Euperipatoides_kanangre
OG50DappuT1-253
MZ277564_Echiniscus_c-opsin_2
MZ277565_Echiniscus_c-opsin_1
MZ277562_Echiniscus_c-opsin_1
MZ277563_Echiniscus_c-opsin_2
Pleurobrachia_opsin2_FQ011385
Mnemiopsis_opsin2_12047
Pleurobrachia_opsin1
Mnemiopsis_opsin1_13055
VERTEBRATA_Candidate_43209_81252710_3
VERTEBRATA_Candidate_43223_81252711_5
VERTEBRATA_Candidate_32628_53221904_0
VERTEBRATA_Candidate_39305_70150599_0
VERTEBRATA_Candidate_23705_34861257_0
VERTEBRATA_Candidate_51435_103720500_2
VERTEBRATA_Candidate_39315_70150600_0
VERTEBRATA_Candidate_23693_34861256_0
VERTEBRATA_Candidate_23711_34861258_0
VERTEBRATA_Candidate_23687_34861255_0
VERTEBRATA_Candidate_39320_70150601_0
VERTEBRATA_Candidate_25821_39927378_0
VERTEBRATA_Candidate_48141_97696658_4
VERTEBRATA_Candidate_714_770045_2
VERTEBRATA_Candidate_45791_91545463_5
VERTEBRATA_Candidate_32807_53635504_1
VERTEBRATA_Candidate_839_789001_1
VERTEBRATA_Candidate_45404_90871511_2
VERTEBRATA_Candidate_45389_90871510_2
VERTEBRATA_Candidate_895_803429_0
VERTEBRATA_Candidate_42934_80574302_1
VERTEBRATA_Candidate_51420_103717736_0
VERTEBRATA_Candidate_43968_84486774_2
VERTEBRATA_Candidate_52085_104058740_1
VERTEBRATA_Candidate_39112_67913287_3
VERTEBRATA_Candidate_956_821988_0
VERTEBRATA_Candidate_8480_20660199_4
VERTEBRATA_Candidate_38618_66989243_2
VERTEBRATA_Candidate_30118_47975670_2
VERTEBRATA_Candidate_942_821987_0
VERTEBRATA_Candidate_30102_47975602_4
VERTEBRATA_Candidate_45787_91545462_5
VERTEBRATA_Candidate_45775_91545461_5
VERTEBRATA_Candidate_1638_4854197_3
VERTEBRATA_Candidate_1626_4854196_3
VERTEBRATA_Candidate_39332_70164531_1
VERTEBRATA_Candidate_27537_42758441_2
VERTEBRATA_Candidate_51944_104024361_3
VERTEBRATA_Candidate_42733_78306004_0
VERTEBRATA_Candidate_26558_41784281_0
VERTEBRATA_Candidate_24419_35567432_4
VERTEBRATA_Candidate_24409_35567431_4
VERTEBRATA_Candidate_61762_133407666_5
VERTEBRATA_Candidate_61748_133407665_5
VERTEBRATA_Candidate_3152_7479323_2
VERTEBRATA_Candidate_43903_84173068_0
VERTEBRATA_Candidate_51918_104021432_4
VERTEBRATA_Candidate_27703_42997312_5
VERTEBRATA_Candidate_25907_40400692_4
VERTEBRATA_Candidate_61735_133405074_4
VERTEBRATA_Candidate_61715_133405073_4
VERTEBRATA_Candidate_42034_76631315_4
VERTEBRATA_Candidate_42044_76631316_4
VERTEBRATA_Candidate_35740_60649529_3
VERTEBRATA_Candidate_51303_103693439_4
VERTEBRATA_Candidate_35708_60647169_1
VERTEBRATA_Candidate_30459_48264387_1
VERTEBRATA_Candidate_1699_4857388_2
VERTEBRATA_Candidate_41785_75598638_0
VERTEBRATA_Candidate_26585_41793307_1
VERTEBRATA_Candidate_26576_41793306_2
VERTEBRATA_Candidate_26794_41815450_1
VERTEBRATA_Candidate_26811_41815451_1
VERTEBRATA_Candidate_35980_60687950_2
VERTEBRATA_Candidate_45299_90855686_6
VERTEBRATA_Candidate_26747_41808922_3
VERTEBRATA_Candidate_8317_20553568_3
VERTEBRATA_Candidate_8308_20553567_3
135SWSOrla1-247
VERTEBRATA_Candidate_51913_104019674_4
VERTEBRATA_Candidate_35958_60682293_1
VERTEBRATA_Candidate_8297_20553566_3
170RHOOrcu1-247
173RHOMumu1-247
158RHOSasa1-247
VERTEBRATA_Candidate_8467_20655032_0
156RHOLeja1-247
148RHOGaga1-247
VERTEBRATA_Candidate_35777_60650614_1
VERTEBRATA_Candidate_52035_104035463_1
VERTEBRATA_Candidate_8201_20535397_0
217BluTaru44-289
VERTEBRATA_Candidate_61790_133419454_4
VERTEBRATA_Candidate_24597_35746317_0
211SWSDare45-290
204SWSNefo1-247
208SWSTagu46-291
VERTEBRATA_Candidate_52100_104066316_3
VERTEBRATA_Candidate_52279_104066321_3
VERTEBRATA_Candidate_52209_104066319_3
VERTEBRATA_Candidate_52171_104066318_3
VERTEBRATA_Candidate_52237_104066320_3
VERTEBRATA_Candidate_52144_104066317_3
126SWSOrla31-275
128SWSPore31-276
VERTEBRATA_Candidate_52402_104102518_4
VERTEBRATA_Candidate_8249_20546804_3
VERTEBRATA_Candidate_36062_60716151_5
VERTEBRATA_Candidate_51864_104010898_0
VERTEBRATA_Candidate_34099_57557715_0
VERTEBRATA_Candidate_51853_104010184_2
VERTEBRATA_Candidate_8347_20560747_2
VERTEBRATA_Candidate_61970_133484959_4
059LWSGeau18-264
VERTEBRATA_TARA_IOS_50_MAG_00159_000000002658.1.1
VERTEBRATA_TARA_ION_45_MAG_00172_000000003410.3.1
VERTEBRATA_TARA_PSE_93_MAG_00257_000000000765.1.2
054VAoDare1-250
041ParXetr1-250
KFQ61069_1_Pinopsin_partial_Pelecanus_crispus
KFO55992_1_Pinopsin_partial_Corvus_brachyrhynchos
KFZ61120_1_Pinopsin_partial_Podiceps_cristatus
233TMTDare1-227

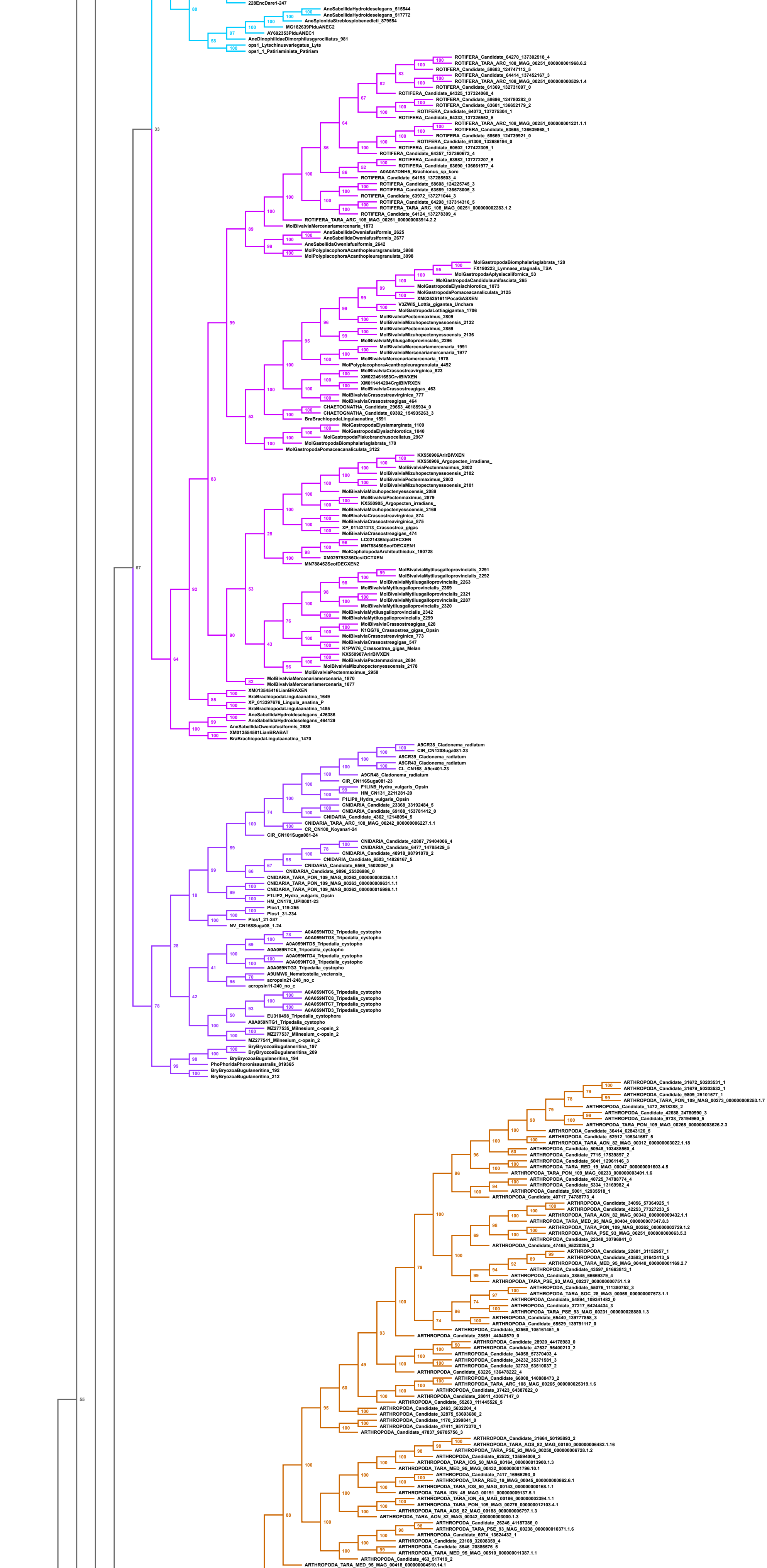

228EncDare1-247
AneSabellidaHydroideselegans_515544
AneSabellidaHydroideselegans_517772
AneSpionidaStreblospiobenedicti_879554
MG182639PlduANEC2
AY692353PlduANEC1
AneDinophilidaeDimorphilusgyrociliatus_981
ops1_Lytechinusvariegatus_Lyte
ops1_1_Patiriaminiata_Patiriam
ROTIFERA_Candidate_64270_137302518_4
ROTIFERA_TARA_ARC_108_MAG_00251_000000001968.6.2
ROTIFERA_Candidate_58683_124747112_5
ROTIFERA_Candidate_64414_137452167_3
ROTIFERA_TARA_ARC_108_MAG_00251_000000000529.1.4
ROTIFERA_Candidate_61369_132731097_0
ROTIFERA_Candidate_64325_137324060_4
ROTIFERA_Candidate_58696_124780282_0
ROTIFERA_Candidate_63681_136652179_2
ROTIFERA_Candidate_64073_137275304_1
ROTIFERA_Candidate_64333_137325552_5
ROTIFERA_TARA_ARC_108_MAG_00251_000000001221.1.1
ROTIFERA_Candidate_63665_136639868_1
ROTIFERA_Candidate_58669_124739921_0
ROTIFERA_Candidate_61308_132686194_0
ROTIFERA_Candidate_60502_127422309_1
ROTIFERA_Candidate_64357_137360673_4
ROTIFERA_Candidate_63982_137272207_5
ROTIFERA_Candidate_63690_136661977_4
A0A0A7DNH5_Brachionus_sp_kore
ROTIFERA_Candidate_64198_137285503_4
ROTIFERA_Candidate_58608_124225745_3
ROTIFERA_Candidate_63589_136578005_3
ROTIFERA_Candidate_63972_137271044_3
ROTIFERA_Candidate_64298_137314316_5
ROTIFERA_TARA_ARC_108_MAG_00251_000000002283.1.2
ROTIFERA_Candidate_64124_137278309_4
ROTIFERA_TARA_ARC_108_MAG_00251_000000003914.2.2
MolBivalviaMercenariamercenaria_1873
AneSabellidaOweniafusiformis_2625
AneSabellidaOweniafusiformis_2677
AneSabellidaOweniafusiformis_2642
MolPolyplacophoraAcanthopleuragranulata_3988
MolPolyplacophoraAcanthopleuragranulata_3998
MolGastropodaBiomphalariaglabrata_128
FX190223_Lymnaea_stagnalis_TSA
MolGastropodaAplysiacalifornica_53
MolGastropodaCandidulaunifasciata_265
MolGastropodaElysiachlorotica_1073
MolGastropodaPomaceacanaliculata_3125
XM025251611PocaGASXEN
V3ZWI5_Lottia_gigantea_Unchara
MolGastropodaLottiagigantea_1706
MolBivalviaPectenmaximus_2809
MolBivalviaMizuhopectenyessoensis_2132
MolBivalviaPectenmaximus_2859
MolBivalviaMizuhopectenyessoensis_2136
MolBivalviaMytilusgalloprovincialis_2296
MolBivalviaMercenariamercenaria_1991
MolBivalviaMercenariamercenaria_1977
MolBivalviaMercenariamercenaria_1978
MolPolyplacophoraAcanthopleuragranulata_4492
MolBivalviaCrassostreavirginica_823
XM022461653CrviBIVXEN
XM011414204CrgiBIVXEN
MolBivalviaCrassostreagigas_463
MolBivalviaCrassostreavirginica_777
MolBivalviaCrassostreagigas_464
CHAETOGNATHA_Candidate_29653_46185934_0
CHAETOGNATHA_Candidate_69302_154935263_3
BraBrachiopodaLingulaanatina_1591
MolGastropodaElysiamarginata_1109
MolGastropodaElysiachlorotica_1040
MolGastropodaPlakobranchusocellatus_2967
MolGastropodaBiomphalariaglabrata_170
MolGastropodaPomaceacanaliculata_3122
KX550906ArirBIVXEN
KX550906_Argopecten_irradians_
MolBivalviaPectenmaximus_2802
MolBivalviaMizuhopectenyessoensis_2102
MolBivalviaPectenmaximus_2803
MolBivalviaMizuhopectenyessoensis_2101
MolBivalviaMizuhopectenyessoensis_2089
MolBivalviaPectenmaximus_2879
KX550905_Argopecten_irradians_
MolBivalviaMizuhopectenyessoensis_2169
MolBivalviaCrassostreavirginica_874
MolBivalviaCrassostreavirginica_875
XP_011421213_Crassostrea_gigas
MolBivalviaCrassostreagigas_474
LC021436IdpaDECXEN
MN788450SeofDECXEN1
MolCephalopodaArchiteuthisdux_190728
XM029798286OcsiOCTXEN
MN788452SeofDECXEN2
MolBivalviaMytilusgalloprovincialis_2291
MolBivalviaMytilusgalloprovincialis_2292
MolBivalviaMytilusgalloprovincialis_2263
MolBivalviaMytilusgalloprovincialis_2369
MolBivalviaMytilusgalloprovincialis_2321
MolBivalviaMytilusgalloprovincialis_2287
MolBivalviaMytilusgalloprovincialis_2320
MolBivalviaMytilusgalloprovincialis_2342
MolBivalviaMytilusgalloprovincialis_2299
MolBivalviaCrassostreagigas_628
K1QG76_Crassostrea_gigas_Opsin
MolBivalviaCrassostreavirginica_773
MolBivalviaCrassostreagigas_547
K1PW76_Crassostrea_gigas_Melan
KX550907ArirBIVXEN
MolBivalviaPectenmaximus_2804
MolBivalviaMizuhopectenyessoensis_2178
MolBivalviaPectenmaximus_2958
MolBivalviaMercenariamercenaria_1870
MolBivalviaMercenariamercenaria_1877
XM013545416LianBRAXEN
BraBrachiopodaLingulaanatina_1649
XP_013397676_Lingula_anatina_P
BraBrachiopodaLingulaanatina_1485
AneSabellidaHydroideselegans_426386
AneSabellidaHydroideselegans_464129
AneSabellidaOweniafusiformis_2688
XM013554581LianBRABAT
BraBrachiopodaLingulaanatina_1470
A9CR38_Cladonema_radiatum
CIR_CN120Suga081-23
A9CR39_Cladonema_radiatum
A9CR43_Cladonema_radiatum
CL_CN168_A9cr401-23
A9CR48_Cladonema_radiatum
CIR_CN116Suga081-23
F1LIN9_Hydra_vulgaris_Opsin
HM_CN131_2211281-20
F1LIP0_Hydra_vulgaris_Opsin
CNIDARIA_Candidate_23368_33192484_5
CNIDARIA_Candidate_69188_153781412_0
CNIDARIA_Candidate_4362_12148094_5
CNIDARIA_TARA_ARC_108_MAG_00242_000000006227.1.1
CR_CN100_Koyana1-24
CIR_CN101Suga081-24
CNIDARIA_Candidate_42887_79404006_4
CNIDARIA_Candidate_6477_14785429_5
CNIDARIA_Candidate_48918_98791079_2
CNIDARIA_Candidate_6503_14826167_5
CNIDARIA_Candidate_6569_15020367_5
CNIDARIA_Candidate_9896_25326986_0
CNIDARIA_TARA_PON_109_MAG_00263_000000008236.1.1
CNIDARIA_TARA_PON_109_MAG_00263_000000009631.1.1
CNIDARIA_TARA_PON_109_MAG_00263_000000015986.1.1
F1LIP2_Hydra_vulgaris_Opsin
HM_CN170_UPI0001-23
Plos1_119-255
Plos1_31-234
Plos1_21-247
NV_CN158Suga08_1-24
A0A059NTD2_Tripedalia_cystopho
A0A059NTG8_Tripedalia_cystopho
A0A059NTD5_Tripedalia_cystopho
A0A059NTC5_Tripedalia_cystopho
A0A059NTD4_Tripedalia_cystopho
A0A059NTG9_Tripedalia_cystopho
A0A059NTG3_Tripedalia_cystopho
A9UMW6_Nematostella_vectensis_
acropsin21-248_no_c
acropsin11-240_no_c
A0A059NTC6_Tripedalia_cystopho
A0A059NTC8_Tripedalia_cystopho
A0A059NTC7_Tripedalia_cystopho
A0A059NTD3_Tripedalia_cystopho
EU310498_Tripedalia_cystophora
A0A059NTG1_Tripedalia_cystopho
MZ277535_Milnesium_c-opsin_2
MZ277537_Milnesium_c-opsin_2
MZ277541_Milnesium_c-opsin_2
BryBryozoaBugulaneritina_197
BryBryozoaBugulaneritina_209
BryBryozoaBugulaneritina_194
PhoPhoridaPhoronisaustralis_819365
BryBryozoaBugulaneritina_192
BryBryozoaBugulaneritina_212
ARTHROPODA_Candidate_31672_50203531_1
ARTHROPODA_Candidate_31679_50203532_1
ARTHROPODA_Candidate_9809_25101577_1
ARTHROPODA_TARA_PON_109_MAG_00273_000000008253.1.7
ARTHROPODA_Candidate_1472_2618288_2
ARTHROPODA_Candidate_42688_24780990_3
ARTHROPODA_Candidate_9738_78194960_5
ARTHROPODA_TARA_PON_109_MAG_00265_000000003626.2.3
ARTHROPODA_Candidate_36414_62843126_5
ARTHROPODA_Candidate_52912_105341657_5
ARTHROPODA_TARA_AON_82_MAG_00312_000000003022.1.18
ARTHROPODA_Candidate_50948_103488560_4
ARTHROPODA_Candidate_7715_17539897_2
ARTHROPODA_Candidate_5041_12961146_3
ARTHROPODA_TARA_RED_19_MAG_00047_000000001603.4.5
ARTHROPODA_TARA_PON_109_MAG_00233_000000003401.1.6
ARTHROPODA_Candidate_40725_74788774_4
ARTHROPODA_Candidate_5334_13169982_4
ARTHROPODA_Candidate_5001_12935518_1
ARTHROPODA_Candidate_40717_74788773_4
ARTHROPODA_Candidate_34056_57364925_1
ARTHROPODA_Candidate_42253_77327233_5
ARTHROPODA_TARA_AON_82_MAG_00343_000000009432.1.1
ARTHROPODA_TARA_MED_95_MAG_00404_000000007347.8.3
ARTHROPODA_TARA_PON_109_MAG_00262_000000002729.1.2
ARTHROPODA_TARA_PSE_93_MAG_00251_000000000063.5.3
ARTHROPODA_Candidate_22348_30796941_0
ARTHROPODA_Candidate_47465_95220255_2
ARTHROPODA_Candidate_22601_31152957_1
ARTHROPODA_Candidate_43583_81642413_5
ARTHROPODA_TARA_MED_95_MAG_00440_000000001169.2.7
ARTHROPODA_Candidate_43597_81663813_1
ARTHROPODA_Candidate_38545_66669379_4
ARTHROPODA_TARA_PSE_93_MAG_00237_000000000751.1.9
ARTHROPODA_Candidate_55076_111380752_3
ARTHROPODA_TARA_SOC_28_MAG_00058_000000007573.1.1
ARTHROPODA_Candidate_54894_109341482_0
ARTHROPODA_Candidate_37217_64244434_3
ARTHROPODA_TARA_PSE_93_MAG_00231_000000028880.1.3
ARTHROPODA_Candidate_65440_139777858_3
ARTHROPODA_Candidate_65529_139791117_0
ARTHROPODA_Candidate_52568_105161451_5
ARTHROPODA_Candidate_28591_44040570_0
ARTHROPODA_Candidate_28920_44178983_0
ARTHROPODA_Candidate_47537_95400213_2
ARTHROPODA_Candidate_34058_57370403_4
ARTHROPODA_Candidate_24232_35371581_3
ARTHROPODA_Candidate_32733_53510037_2
ARTHROPODA_Candidate_63226_136478222_4
ARTHROPODA_Candidate_66008_140888473_2
ARTHROPODA_TARA_ARC_108_MAG_00265_000000025319.1.6
ARTHROPODA_Candidate_37423_64387822_0
ARTHROPODA_Candidate_28011_43057147_0
ARTHROPODA_Candidate_55263_111445526_5
ARTHROPODA_Candidate_2463_5632204_4
ARTHROPODA_Candidate_32875_53693680_2
ARTHROPODA_Candidate_1170_2399841_0
ARTHROPODA_Candidate_47411_95172370_1
ARTHROPODA_Candidate_47837_96705756_3
ARTHROPODA_Candidate_31664_50195893_2
ARTHROPODA_TARA_AOS_82_MAG_00180_000000006482.1.16
ARTHROPODA_TARA_PSE_93_MAG_00250_000000006728.1.2
ARTHROPODA_Candidate_62522_135594009_3
ARTHROPODA_TARA_IOS_50_MAG_00164_000000013900.1.3
ARTHROPODA_TARA_MED_95_MAG_00432_000000001796.10.1
ARTHROPODA_Candidate_7417_16965293_0
ARTHROPODA_TARA_RED_19_MAG_00045_000000000862.6.1
ARTHROPODA_TARA_IOS_50_MAG_00143_000000000168.1.1
ARTHROPODA_TARA_ION_45_MAG_00191_000000009137.5.1
ARTHROPODA_TARA_ION_45_MAG_00186_000000002394.1.1
ARTHROPODA_TARA_PON_109_MAG_00276_000000012103.4.1
ARTHROPODA_TARA_AOS_82_MAG_00188_000000006797.1.3
ARTHROPODA_TARA_AON_82_MAG_00342_000000003000.1.3
ARTHROPODA_Candidate_26246_41187386_0
ARTHROPODA_TARA_PSE_93_MAG_00238_000000010371.1.6
ARTHROPODA_Candidate_6074_13624432_1
ARTHROPODA_Candidate_23108_32608359_4
ARTHROPODA_Candidate_8546_20886576_5
ARTHROPODA_TARA_MED_95_MAG_00510_000000011387.1.1
ARTHROPODA_Candidate_463_517419_2
ARTHROPODA_TARA_MED_95_MAG_00418_000000004510.14.1

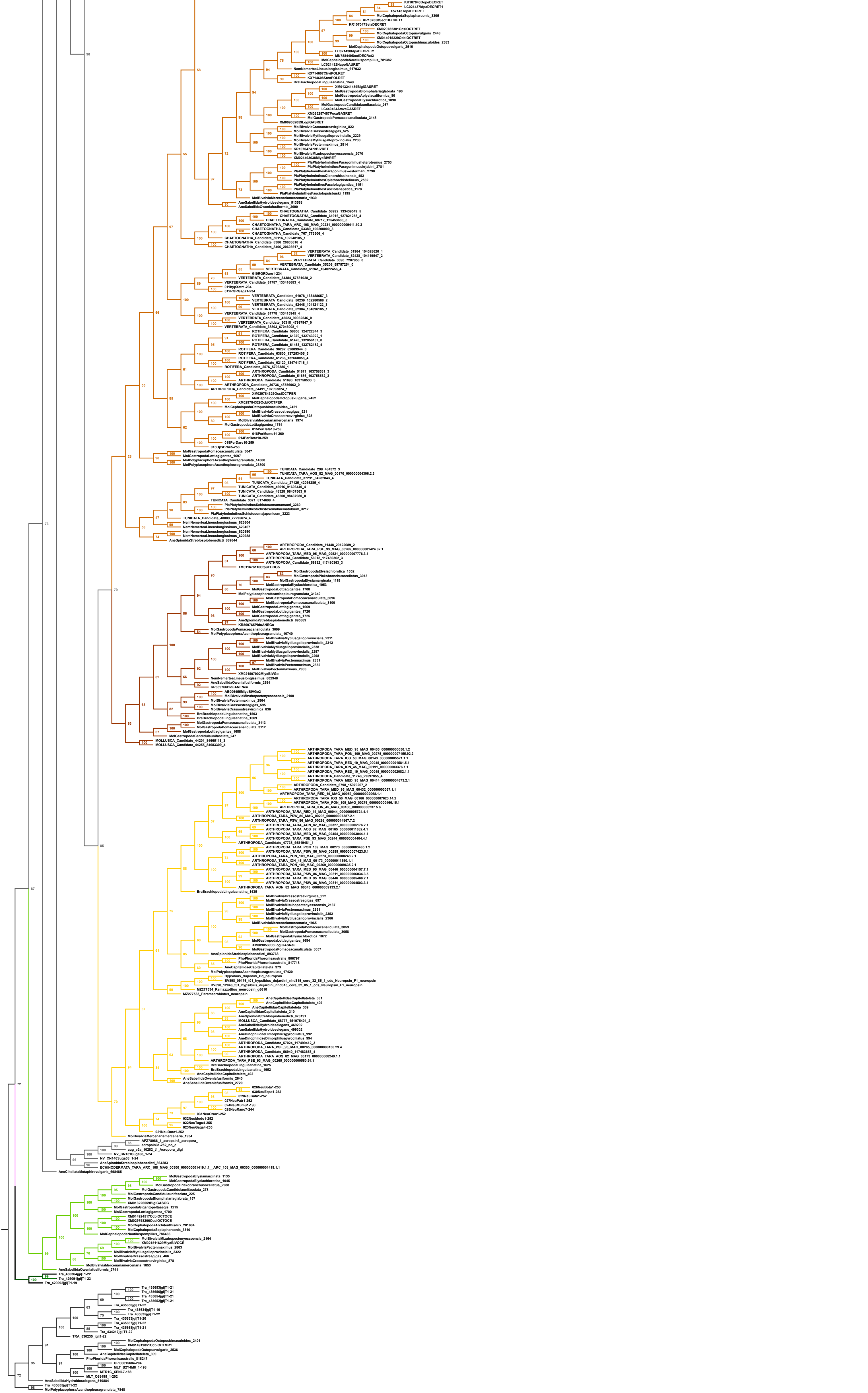

Supplement: evaf189_Supplementary_Data [file Figure_S2.pdf › Figure_S2.pdf]
