# Supplementary material for: An Ocean of Opsins
Source: Genome Biol Evol. 2025 Nov 4;17(11):evaf189. doi: 10.1093/gbe/evaf189 (PMC12584886; doi:10.1093/gbe/evaf189)
Supplement: evaf189_Supplementary_Data [file Figure_S3.pdf › Figure_S3.pdf]

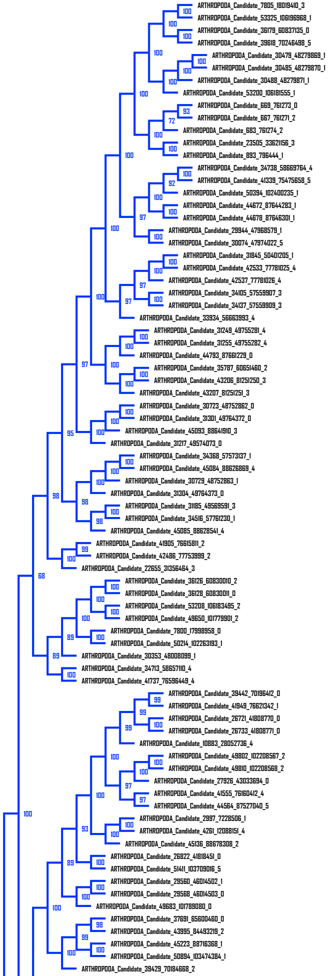
ARTHROPODA_Candidate_7805_18019410_3
ARTHROPODA_Candidate_53325_106196968_1
ARTHROPODA_Candidate_36179_60837135_0
ARTHROPODA_Candidate_39618_70246498_5
ARTHROPODA_Candidate_30479_48279869_1
ARTHROPODA_Candidate_30485_48279870_1
ARTHROPODA_Candidate_30488_48279871_1
ARTHROPODA_Candidate_53200_106181555_1
ARTHROPODA_Candidate_669_761273_0
ARTHROPODA_Candidate_667_761271_2
ARTHROPODA_Candidate_683_761274_2
ARTHROPODA_Candidate_23505_33621156_3
ARTHROPODA_Candidate_893_796444_1
ARTHROPODA_Candidate_34738_58669764_4
ARTHROPODA_Candidate_41339_75475658_5
ARTHROPODA_Candidate_50394_102400235_1
ARTHROPODA_Candidate_44672_87644283_1
ARTHROPODA_Candidate_44678_87646301_1
ARTHROPODA_Candidate_29944_47968579_1
ARTHROPODA_Candidate_30074_47974022_5
ARTHROPODA_Candidate_31845_50401205_1
ARTHROPODA_Candidate_42533_77781025_4
ARTHROPODA_Candidate_42537_77781026_4
ARTHROPODA_Candidate_34105_57559907_3
ARTHROPODA_Candidate_34137_57559909_3
ARTHROPODA_Candidate_33934_56663993_4
ARTHROPODA_Candidate_31249_49755281_4
ARTHROPODA_Candidate_31255_49755282_4
ARTHROPODA_Candidate_44793_87661229_0
ARTHROPODA_Candidate_35787_60651460_2
ARTHROPODA_Candidate_43206_81251250_3
ARTHROPODA_Candidate_43207_81251251_3
ARTHROPODA_Candidate_30723_48752862_0
ARTHROPODA_Candidate_31301_49764372_0
ARTHROPODA_Candidate_45093_88641910_3
ARTHROPODA_Candidate_31217_49574073_0
ARTHROPODA_Candidate_34368_57573137_1
ARTHROPODA_Candidate_45084_88626869_4
ARTHROPODA_Candidate_30729_48752863_1
ARTHROPODA_Candidate_31304_49764373_0
ARTHROPODA_Candidate_31185_49569591_3
ARTHROPODA_Candidate_34516_57761230_1
ARTHROPODA_Candidate_45085_88628541_4
ARTHROPODA_Candidate_41905_76615811_2
ARTHROPODA_Candidate_42486_77753999_2
ARTHROPODA_Candidate_22655_31356464_3
ARTHROPODA_Candidate_36126_60830010_2
ARTHROPODA_Candidate_36128_60830011_0
ARTHROPODA_Candidate_53208_106183495_2
ARTHROPODA_Candidate_49650_101779901_2
ARTHROPODA_Candidate_7800_17998958_0
ARTHROPODA_Candidate_50214_102263193_1
ARTHROPODA_Candidate_30353_48008099_1
ARTHROPODA_Candidate_34713_58657110_4
ARTHROPODA_Candidate_41737_76596449_4
ARTHROPODA_Candidate_39442_70196412_0
ARTHROPODA_Candidate_41949_76621342_1
ARTHROPODA_Candidate_26721_41808770_0
ARTHROPODA_Candidate_26733_41808771_0
ARTHROPODA_Candidate_10883_28052736_4
ARTHROPODA_Candidate_49802_102208567_2
ARTHROPODA_Candidate_49810_102208568_2
ARTHROPODA_Candidate_27926_43033694_0
ARTHROPODA_Candidate_41555_76160412_4
ARTHROPODA_Candidate_44564_87527040_5
ARTHROPODA_Candidate_2997_7228506_1
ARTHROPODA_Candidate_4261_12088151_4
ARTHROPODA_Candidate_45136_88678308_2
ARTHROPODA_Candidate_26822_41818451_0
ARTHROPODA_Candidate_51411_103709016_5
ARTHROPODA_Candidate_29560_46014502_1
ARTHROPODA_Candidate_29568_46014503_0
ARTHROPODA_Candidate_49683_101789080_0
ARTHROPODA_Candidate_37691_65600460_0
ARTHROPODA_Candidate_43995_84493219_2
ARTHROPODA_Candidate_45223_88716368_1
ARTHROPODA_Candidate_50894_103474384_1
ARTHROPODA_Candidate_39429_70184668_2

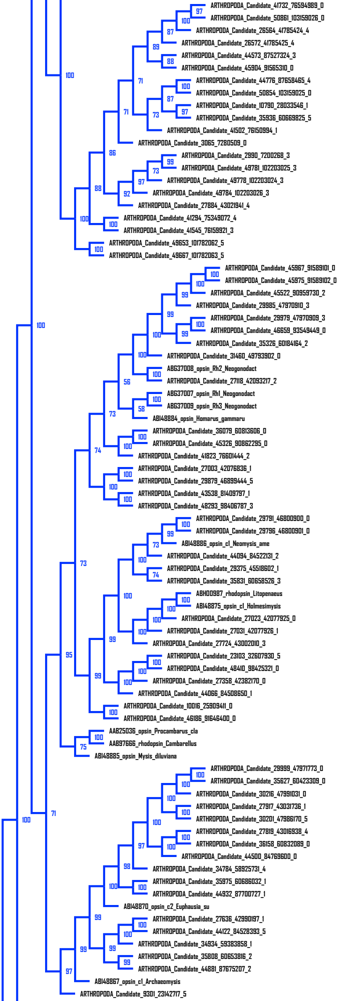

ARTHROPODA_Candidate_41732_76594989_0
ARTHROPODA_Candidate_50861_103159026_0
ARTHROPODA_Candidate_26564_41785424_4
ARTHROPODA_Candidate_26572_41785425_4
ARTHROPODA_Candidate_44573_87527324_3
ARTHROPODA_Candidate_45904_91565310_0
ARTHROPODA_Candidate_44776_87658465_4
ARTHROPODA_Candidate_50854_103159025_0
ARTHROPODA_Candidate_10790_28033546_1
ARTHROPODA_Candidate_35936_60669825_5
ARTHROPODA_Candidate_41502_76150994_1
ARTHROPODA_Candidate_3065_7280509_0
ARTHROPODA_Candidate_2990_7200268_3
ARTHROPODA_Candidate_49781_102203025_3
ARTHROPODA_Candidate_49778_102203024_3
ARTHROPODA_Candidate_49784_102203026_3
ARTHROPODA_Candidate_27884_43021941_4
ARTHROPODA_Candidate_41294_75349072_4
ARTHROPODA_Candidate_41545_76159921_3
ARTHROPODA_Candidate_49653_101782062_5
ARTHROPODA_Candidate_49667_101782063_5
ARTHROPODA_Candidate_45967_91589101_0
ARTHROPODA_Candidate_45975_91589102_0
ARTHROPODA_Candidate_45522_90959730_2
ARTHROPODA_Candidate_29985_47970910_3
ARTHROPODA_Candidate_29979_47970909_3
ARTHROPODA_Candidate_46659_93549449_0
ARTHROPODA_Candidate_35326_60184164_2
ARTHROPODA_Candidate_31460_49793902_0
ABG37008_opsin_Rh2_Neogonodact
ARTHROPODA_Candidate_27118_42093217_2
ABG37007_opsin_Rh1_Neogonodact
ABG37009_opsin_Rh3_Neogonodact
AB148884_opsin_Homarus_gammaru
ARTHROPODA_Candidate_36079_60813606_0
ARTHROPODA_Candidate_45326_90862295_0
ARTHROPODA_Candidate_41823_76601444_2
ARTHROPODA_Candidate_27003_42076836_1
ARTHROPODA_Candidate_29879_46899444_5
ARTHROPODA_Candidate_43538_81409797_1
ARTHROPODA_Candidate_48293_98406787_3
ARTHROPODA_Candidate_29791_46800900_0
ARTHROPODA_Candidate_29796_46800901_0
AB148886_opsin_c1_Neomysis_ame
ARTHROPODA_Candidate_44094_84522131_2
ARTHROPODA_Candidate_29375_45518602_1
ARTHROPODA_Candidate_35831_60658526_3
ABH00987_rhodopsin_Litopenaeus
AB148875_opsin_c1_Holmesimysis
ARTHROPODA_Candidate_27023_42077925_0
ARTHROPODA_Candidate_27031_42077926_1
ARTHROPODA_Candidate_27724_43002010_3
ARTHROPODA_Candidate_23103_32607930_5
ARTHROPODA_Candidate_48410_98425321_0
ARTHROPODA_Candidate_27358_42382170_0
ARTHROPODA_Candidate_44066_84508650_1
ARTHROPODA_Candidate_10016_25909411_0
ARTHROPODA_Candidate_46186_91646400_0
AAB25036_opsin_Procambarus_cla
AAB97666_rhodopsin_Cambarellus
AB148885_opsin_Mysis_diluviana
ARTHROPODA_Candidate_29999_47971773_0
ARTHROPODA_Candidate_35627_60423309_0
ARTHROPODA_Candidate_30216_47991031_0
ARTHROPODA_Candidate_27917_43031736_1
ARTHROPODA_Candidate_30201_47986170_5
ARTHROPODA_Candidate_27819_43016938_4
ARTHROPODA_Candidate_36158_60832089_0
ARTHROPODA_Candidate_44500_84769600_0
ARTHROPODA_Candidate_34784_58925731_4
ARTHROPODA_Candidate_35975_60686032_1
ARTHROPODA_Candidate_44932_87700727_1
AB148870_opsin_c2_Euphausia_su
ARTHROPODA_Candidate_27636_42990197_1
ARTHROPODA_Candidate_44122_84528393_5
ARTHROPODA_Candidate_34934_59383858_1
ARTHROPODA_Candidate_35808_60653816_2
ARTHROPODA_Candidate_44881_87675207_2
AB148867_opsin_c1_Archaeomysis
ARTHROPODA_Candidate_9301_23142717_5

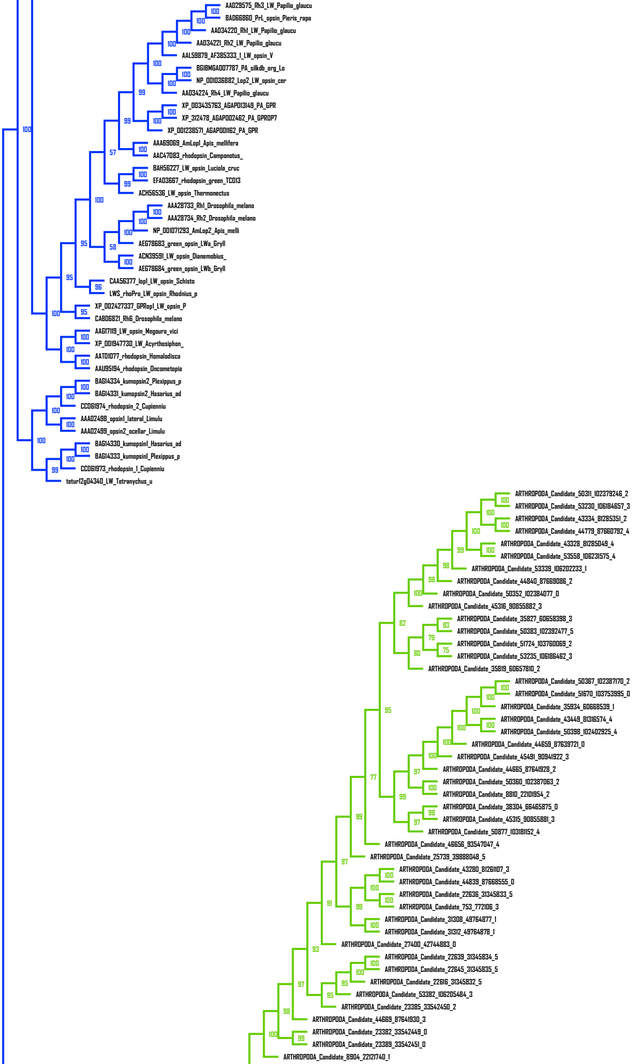
AAD29575_Rh3_LW_Papilio_glaucu
BAD66860_PrL_opsin_Pieris_rapa
AAD34220_Rh1_LW_Papilio_glaucu
AAD34221_Rh2_LW_Papilio_glaucu
AAL59879_AF385333_1_LW_opsin_V
BGIBMGA007787_PA_silkdb_org_Lo
NP_001036882_Lop2_LW_opsin_cer
AAD34224_Rh4_LW_Papilio_glaucu
XP_003435763_AGAP013149_PA_GPR
XP_312478_AGAP002462_PA_GPROP7
XP_001238571_AGAP001162_PA_GPR
AAA69069_AmLop1_Apis_mellifera
AAC47083_rhodopsin_Camponotus_
BAH56227_LW_opsin_Luciola_cruc
EFA03667_rhodopsin_green_TC013
ACH56536_LW_opsin_Thermonectus
AAA28733_Rh1_Drosophila_melano
AAA28734_Rh2_Drosophila_melano
NP_001071293_AmLop2_Apis_melli
AEG78683_green_opsin_LWa_Gryll
ACN39591_LW_opsin_Dianemobius_
AEG78684_green_opsin_LWb_Gryll
CAA56377_lop1_LW_opsin_Schisto
LWS_rhoPro_LW_opsin_Rhodnius_p
XP_002427337_GPRop1_LW_opsin_P
CAB06821_Rh6_Drosophila_melano
AAG17119_LW_opsin_Megoura_vici
XP_001947730_LW_Acyrthosiphon_
AAT01077_rhodopsin_Homalodisca
AAU95194_rhodopsin_Oncometopia
BAG14334_kumopsin2_Plexippus_p
BAG14331_kumopsin2_Hasarius_ad
CCD61974_rhodopsin_2_Cupienniu
AAA02498_opsin1_lateral_Limulu
AAA02499_opsin2_ocellar_Limulu
BAG14330_kumopsin1_Hasarius_ad
BAG14333_kumopsin1_Plexippus_p
CCD61973_rhodopsin_1_Cupienniu
tetur12g04340_LW_Tetranychus_u
ARTHROPODA_Candidate_50311_102379246_2
ARTHROPODA_Candidate_53230_106184657_3
ARTHROPODA_Candidate_43334_81285351_2
ARTHROPODA_Candidate_44779_87660792_4
ARTHROPODA_Candidate_43328_81285049_4
ARTHROPODA_Candidate_53558_106231575_4
ARTHROPODA_Candidate_53339_106202233_1
ARTHROPODA_Candidate_44840_87669086_2
ARTHROPODA_Candidate_50352_102384077_0
ARTHROPODA_Candidate_45316_90855882_3
ARTHROPODA_Candidate_35827_60658398_3
ARTHROPODA_Candidate_50383_102392477_5
ARTHROPODA_Candidate_51724_103760069_2
ARTHROPODA_Candidate_53235_106186462_3
ARTHROPODA_Candidate_35819_60657810_2
ARTHROPODA_Candidate_50367_102387170_2
ARTHROPODA_Candidate_51670_103753995_0
ARTHROPODA_Candidate_35934_60668539_1
ARTHROPODA_Candidate_43449_81316574_4
ARTHROPODA_Candidate_50398_102402925_4
ARTHROPODA_Candidate_44659_87639721_0
ARTHROPODA_Candidate_45491_90941922_3
ARTHROPODA_Candidate_44665_87641928_2
ARTHROPODA_Candidate_50360_102387063_2
ARTHROPODA_Candidate_8810_22101954_2
ARTHROPODA_Candidate_38304_66465875_0
ARTHROPODA_Candidate_45315_90855881_3
ARTHROPODA_Candidate_50877_103181152_4
ARTHROPODA_Candidate_46656_93547047_4
ARTHROPODA_Candidate_25739_39888048_5
ARTHROPODA_Candidate_43280_81261107_3
ARTHROPODA_Candidate_44839_87668555_0
ARTHROPODA_Candidate_22636_31345833_5
ARTHROPODA_Candidate_753_772106_3
ARTHROPODA_Candidate_31308_49764877_1
ARTHROPODA_Candidate_31312_49764878_1
ARTHROPODA_Candidate_27400_42744883_0
ARTHROPODA_Candidate_22639_31345834_5
ARTHROPODA_Candidate_22645_31345835_5
ARTHROPODA_Candidate_22616_31345832_5
ARTHROPODA_Candidate_53382_106205484_3
ARTHROPODA_Candidate_23385_33542450_2
ARTHROPODA_Candidate_44669_87641930_3
ARTHROPODA_Candidate_23382_33542449_0
ARTHROPODA_Candidate_23389_33542451_0
ARTHROPODA_Candidate_8904_22121740_1

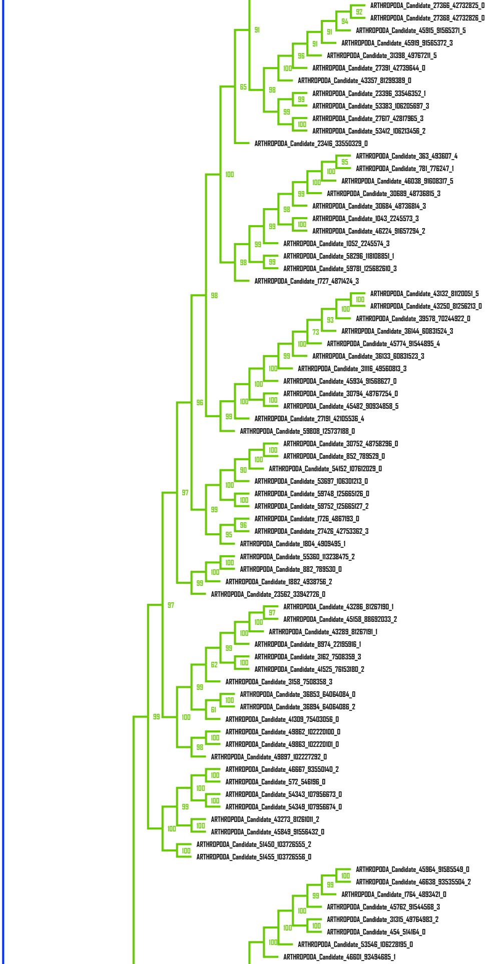
ARTHROPODA_Candidate_27366_42732825_0
ARTHROPODA_Candidate_27368_42732826_0
ARTHROPODA_Candidate_45915_91565371_5
ARTHROPODA_Candidate_45919_91565372_3
ARTHROPODA_Candidate_31398_49767211_5
ARTHROPODA_Candidate_27391_42739644_0
ARTHROPODA_Candidate_43357_81299389_0
ARTHROPODA_Candidate_23396_33546352_1
ARTHROPODA_Candidate_53383_106205697_3
ARTHROPODA_Candidate_27617_42817965_3
ARTHROPODA_Candidate_53412_106213456_2
ARTHROPODA_Candidate_23416_33550329_0
ARTHROPODA_Candidate_363_493607_4
ARTHROPODA_Candidate_781_776247_1
ARTHROPODA_Candidate_46038_91608317_5
ARTHROPODA_Candidate_30689_48736815_3
ARTHROPODA_Candidate_30684_48736814_3
ARTHROPODA_Candidate_1043_2245573_3
ARTHROPODA_Candidate_46224_91657294_2
ARTHROPODA_Candidate_1052_2245574_3
ARTHROPODA_Candidate_58296_118108851_1
ARTHROPODA_Candidate_59781_125682610_3
ARTHROPODA_Candidate_1727_4871424_3
ARTHROPODA_Candidate_43132_81120051_5
ARTHROPODA_Candidate_43250_81256213_0
ARTHROPODA_Candidate_39578_70244922_0
ARTHROPODA_Candidate_36144_60831524_3
ARTHROPODA_Candidate_45774_91544895_4
ARTHROPODA_Candidate_36133_60831523_3
ARTHROPODA_Candidate_31116_49560813_3
ARTHROPODA_Candidate_45934_91568627_0
ARTHROPODA_Candidate_30794_48767254_0
ARTHROPODA_Candidate_45482_90934858_5
ARTHROPODA_Candidate_27191_42105536_4
ARTHROPODA_Candidate_59808_125737188_0
ARTHROPODA_Candidate_30752_48758296_0
ARTHROPODA_Candidate_852_789529_0
ARTHROPODA_Candidate_54152_107612029_0
ARTHROPODA_Candidate_53697_106301213_0
ARTHROPODA_Candidate_59748_125665126_0
ARTHROPODA_Candidate_59752_125665127_2
ARTHROPODA_Candidate_1726_4867193_0
ARTHROPODA_Candidate_27426_42753362_3
ARTHROPODA_Candidate_1804_4909495_1
ARTHROPODA_Candidate_55360_113238475_2
ARTHROPODA_Candidate_882_789530_0
ARTHROPODA_Candidate_1882_4938756_2
ARTHROPODA_Candidate_23562_33942726_0
ARTHROPODA_Candidate_43286_81267190_1
ARTHROPODA_Candidate_45158_88692033_2
ARTHROPODA_Candidate_43289_81267191_1
ARTHROPODA_Candidate_8974_22195916_1
ARTHROPODA_Candidate_3162_7508359_3
ARTHROPODA_Candidate_41525_76153180_2
ARTHROPODA_Candidate_3158_7508358_3
ARTHROPODA_Candidate_36853_64064084_0
ARTHROPODA_Candidate_36894_64064086_2
ARTHROPODA_Candidate_41309_75403056_0
ARTHROPODA_Candidate_49862_102220100_0
ARTHROPODA_Candidate_49863_102220101_0
ARTHROPODA_Candidate_49897_102227292_0
ARTHROPODA_Candidate_46667_93550140_2
ARTHROPODA_Candidate_572_546196_0
ARTHROPODA_Candidate_54343_107956673_0
ARTHROPODA_Candidate_54349_107956674_0
ARTHROPODA_Candidate_43273_81261011_2
ARTHROPODA_Candidate_45849_91556432_0
ARTHROPODA_Candidate_51450_103726555_2
ARTHROPODA_Candidate_51455_103726556_0
ARTHROPODA_Candidate_45964_91585549_0
ARTHROPODA_Candidate_46638_93535504_2
ARTHROPODA_Candidate_1764_4893421_0
ARTHROPODA_Candidate_45762_91544568_3
ARTHROPODA_Candidate_31315_49764983_2
ARTHROPODA_Candidate_454_514164_0
ARTHROPODA_Candidate_53546_106228195_0
ARTHROPODA_Candidate_46601_93494685_1

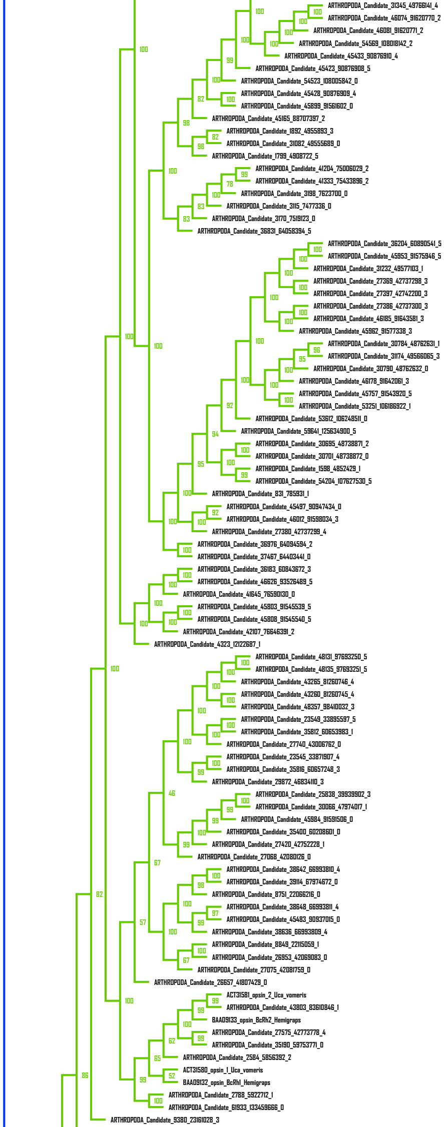
ARTHROPODA_Candidate_31345_49766141_4
ARTHROPODA_Candidate_46074_91620770_2
ARTHROPODA_Candidate_46081_91620771_2
ARTHROPODA_Candidate_54569_108018142_2
ARTHROPODA_Candidate_45433_90876910_4
ARTHROPODA_Candidate_45423_90876908_5
ARTHROPODA_Candidate_54523_108005842_0
ARTHROPODA_Candidate_45428_90876909_4
ARTHROPODA_Candidate_45899_91561602_0
ARTHROPODA_Candidate_45165_88707397_2
ARTHROPODA_Candidate_1892_4955893_3
ARTHROPODA_Candidate_31082_49555689_0
ARTHROPODA_Candidate_1799_4908722_5
ARTHROPODA_Candidate_41204_75006029_2
ARTHROPODA_Candidate_41333_75433896_2
ARTHROPODA_Candidate_3198_7623700_0
ARTHROPODA_Candidate_3115_7477336_0
ARTHROPODA_Candidate_3170_7519123_0
ARTHROPODA_Candidate_36831_64058394_5
ARTHROPODA_Candidate_36204_60890541_5
ARTHROPODA_Candidate_45953_91575946_5
ARTHROPODA_Candidate_31232_49577103_1
ARTHROPODA_Candidate_27369_42737298_3
ARTHROPODA_Candidate_27397_42742200_3
ARTHROPODA_Candidate_27386_42737300_3
ARTHROPODA_Candidate_46185_91643581_3
ARTHROPODA_Candidate_45962_91577338_3
ARTHROPODA_Candidate_30784_48762631_1
ARTHROPODA_Candidate_31174_49566065_3
ARTHROPODA_Candidate_30790_48762632_0
ARTHROPODA_Candidate_46178_91642061_3
ARTHROPODA_Candidate_45757_91543920_5
ARTHROPODA_Candidate_53251_106186922_1
ARTHROPODA_Candidate_53612_106248511_0
ARTHROPODA_Candidate_59641_125634900_5
ARTHROPODA_Candidate_30695_48738871_2
ARTHROPODA_Candidate_30701_48738872_0
ARTHROPODA_Candidate_1598_4852429_1
ARTHROPODA_Candidate_54204_107627530_5
ARTHROPODA_Candidate_831_785931_1
ARTHROPODA_Candidate_45497_90947434_0
ARTHROPODA_Candidate_46012_91598034_3
ARTHROPODA_Candidate_27380_42737299_4
ARTHROPODA_Candidate_36976_64094594_2
ARTHROPODA_Candidate_37467_64403441_0
ARTHROPODA_Candidate_36183_60843672_3
ARTHROPODA_Candidate_46626_93526489_5
ARTHROPODA_Candidate_41645_76590130_0
ARTHROPODA_Candidate_45803_91545539_5
ARTHROPODA_Candidate_45808_91545540_5
ARTHROPODA_Candidate_42107_76646391_2
ARTHROPODA_Candidate_4323_12122687_1
ARTHROPODA_Candidate_48131_97693250_5
ARTHROPODA_Candidate_48135_97693251_5
ARTHROPODA_Candidate_43265_81260746_4
ARTHROPODA_Candidate_43260_81260745_4
ARTHROPODA_Candidate_48357_98410032_3
ARTHROPODA_Candidate_23549_33895597_5
ARTHROPODA_Candidate_35812_60653983_1
ARTHROPODA_Candidate_27740_43006762_0
ARTHROPODA_Candidate_23545_33871907_4
ARTHROPODA_Candidate_35816_60657248_3
ARTHROPODA_Candidate_29872_46834110_3
ARTHROPODA_Candidate_25838_39939902_3
ARTHROPODA_Candidate_30066_47974017_1
ARTHROPODA_Candidate_45984_91591506_0
ARTHROPODA_Candidate_35400_60208601_0
ARTHROPODA_Candidate_27420_42752228_1
ARTHROPODA_Candidate_27068_42080126_0
ARTHROPODA_Candidate_38642_66993810_4
ARTHROPODA_Candidate_39114_67974672_0
ARTHROPODA_Candidate_8751_22066216_0
ARTHROPODA_Candidate_38648_66993811_4
ARTHROPODA_Candidate_45483_90937015_0
ARTHROPODA_Candidate_38636_66993809_4
ARTHROPODA_Candidate_8849_22115059_1
ARTHROPODA_Candidate_26953_42069083_0
ARTHROPODA_Candidate_27075_42081759_0
ARTHROPODA_Candidate_26657_41807429_0
ACT31581_opsin_2_Uca_vomeris
ARTHROPODA_Candidate_43803_83610846_1
BAA09133_opsin_BcRh2_Hemigraps
ARTHROPODA_Candidate_27575_42773778_4
ARTHROPODA_Candidate_35190_59753771_0
ARTHROPODA_Candidate_2584_5856392_2
ACT31580_opsin_1_Uca_vomeris
BAA09132_opsin_BcRh1_Hemigraps
ARTHROPODA_Candidate_2788_5922712_1
ARTHROPODA_Candidate_61933_133459666_0
ARTHROPODA_Candidate_9380_23161028_3

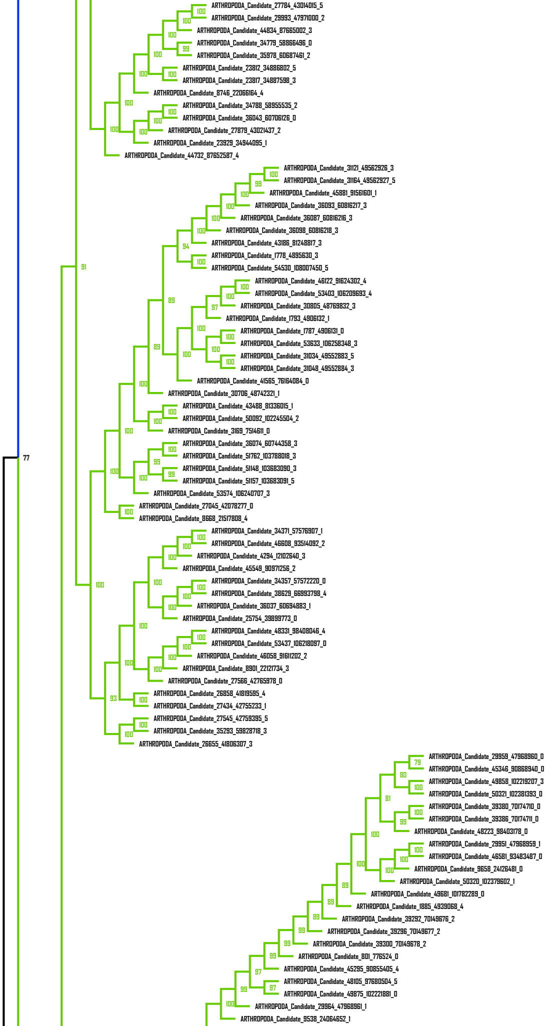
ARTHROPODA_Candidate_27784_43014015_5
ARTHROPODA_Candidate_29993_47971000_2
ARTHROPODA_Candidate_44834_87665002_3
ARTHROPODA_Candidate_34779_58866496_0
ARTHROPODA_Candidate_35978_60687461_2
ARTHROPODA_Candidate_23812_34886802_5
ARTHROPODA_Candidate_23817_34887598_3
ARTHROPODA_Candidate_8746_22066164_4
ARTHROPODA_Candidate_34788_58955535_2
ARTHROPODA_Candidate_36043_60706126_0
ARTHROPODA_Candidate_27879_43021437_2
ARTHROPODA_Candidate_23929_34944095_1
ARTHROPODA_Candidate_44732_87652587_4
ARTHROPODA_Candidate_31121_49562926_3
ARTHROPODA_Candidate_31164_49562927_5
ARTHROPODA_Candidate_45881_91561601_1
ARTHROPODA_Candidate_36093_60816217_3
ARTHROPODA_Candidate_36087_60816216_3
ARTHROPODA_Candidate_36098_60816218_3
ARTHROPODA_Candidate_43186_81248817_3
ARTHROPODA_Candidate_1778_4895630_3
ARTHROPODA_Candidate_54530_108007450_5
ARTHROPODA_Candidate_46122_91624302_4
ARTHROPODA_Candidate_53403_106209693_4
ARTHROPODA_Candidate_30805_48769832_3
ARTHROPODA_Candidate_1793_4906132_1
ARTHROPODA_Candidate_1787_4906131_0
ARTHROPODA_Candidate_53633_106258348_3
ARTHROPODA_Candidate_31034_49552883_5
ARTHROPODA_Candidate_31048_49552884_3
ARTHROPODA_Candidate_41565_76164084_0
ARTHROPODA_Candidate_30706_48742321_1
ARTHROPODA_Candidate_43488_81336015_1
ARTHROPODA_Candidate_50092_102245504_2
ARTHROPODA_Candidate_3169_7514611_0
ARTHROPODA_Candidate_36074_60744358_3
ARTHROPODA_Candidate_51762_103788018_3
ARTHROPODA_Candidate_51148_103683090_3
ARTHROPODA_Candidate_51157_103683091_5
ARTHROPODA_Candidate_53574_106240707_3
ARTHROPODA_Candidate_27045_42078277_0
ARTHROPODA_Candidate_8668_21517808_4
ARTHROPODA_Candidate_34371_57576907_1
ARTHROPODA_Candidate_46608_93514092_2
ARTHROPODA_Candidate_4294_12102640_3
ARTHROPODA_Candidate_45549_90971256_2
ARTHROPODA_Candidate_34357_57572220_0
ARTHROPODA_Candidate_38629_66993798_4
ARTHROPODA_Candidate_36037_60694883_1
ARTHROPODA_Candidate_25754_39899773_0
ARTHROPODA_Candidate_48331_98408046_4
ARTHROPODA_Candidate_53437_106218097_0
ARTHROPODA_Candidate_46058_91611202_2
ARTHROPODA_Candidate_8901_22121734_3
ARTHROPODA_Candidate_27566_42765978_0
ARTHROPODA_Candidate_26858_41819595_4
ARTHROPODA_Candidate_27434_42755233_1
ARTHROPODA_Candidate_27545_42759395_5
ARTHROPODA_Candidate_35293_59828718_3
ARTHROPODA_Candidate_26655_41806307_3
ARTHROPODA_Candidate_29959_47968960_0
ARTHROPODA_Candidate_45346_90868940_0
ARTHROPODA_Candidate_49858_102219207_3
ARTHROPODA_Candidate_50321_102381393_0
ARTHROPODA_Candidate_39380_70174710_0
ARTHROPODA_Candidate_39386_70174711_0
ARTHROPODA_Candidate_48223_98403178_0
ARTHROPODA_Candidate_29951_47968959_1
ARTHROPODA_Candidate_46581_93483487_0
ARTHROPODA_Candidate_9658_24126481_0
ARTHROPODA_Candidate_50320_102379602_1
ARTHROPODA_Candidate_49681_101782289_0
ARTHROPODA_Candidate_1885_4939068_4
ARTHROPODA_Candidate_39292_70149676_2
ARTHROPODA_Candidate_39296_70149677_2
ARTHROPODA_Candidate_39300_70149678_2
ARTHROPODA_Candidate_801_776524_0
ARTHROPODA_Candidate_45295_90855405_4
ARTHROPODA_Candidate_48105_97680504_5
ARTHROPODA_Candidate_49875_102221881_0
ARTHROPODA_Candidate_29964_47968961_1
ARTHROPODA_Candidate_9538_24064652_1

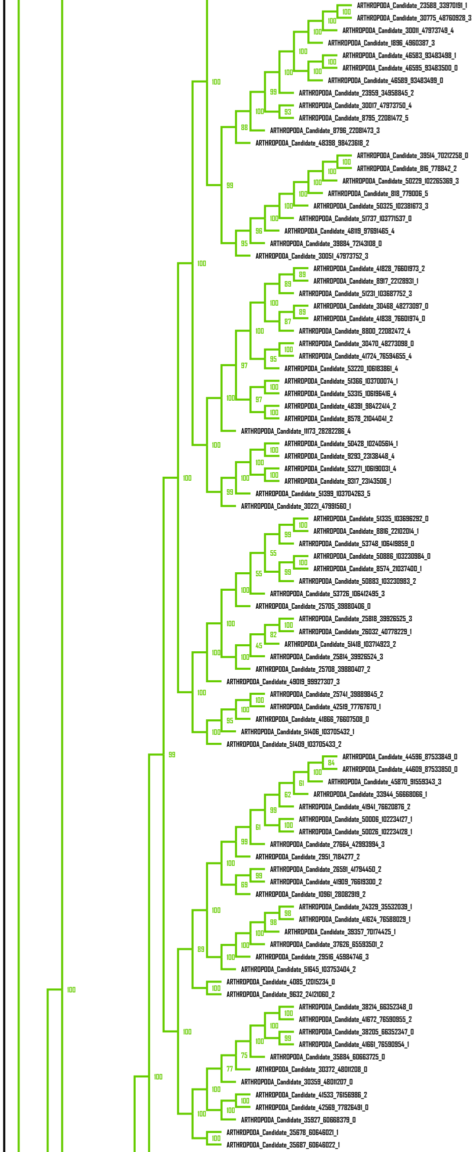
ARTHROPODA_Candidate_23588_33970191_1
ARTHROPODA_Candidate_30775_48760928_3
ARTHROPODA_Candidate_30011_47973749_4
ARTHROPODA_Candidate_1896_4960387_3
ARTHROPODA_Candidate_46583_93483498_1
ARTHROPODA_Candidate_46595_93483500_0
ARTHROPODA_Candidate_46589_93483499_0
ARTHROPODA_Candidate_23959_34958845_2
ARTHROPODA_Candidate_30017_47973750_4
ARTHROPODA_Candidate_8795_22081472_5
ARTHROPODA_Candidate_8796_22081473_3
ARTHROPODA_Candidate_48398_98423618_2
ARTHROPODA_Candidate_39514_70212258_0
ARTHROPODA_Candidate_816_778842_2
ARTHROPODA_Candidate_50229_102265369_3
ARTHROPODA_Candidate_818_779006_5
ARTHROPODA_Candidate_50325_102381673_3
ARTHROPODA_Candidate_51737_103771537_0
ARTHROPODA_Candidate_48119_97691465_4
ARTHROPODA_Candidate_39884_72143108_0
ARTHROPODA_Candidate_30051_47973752_3
ARTHROPODA_Candidate_41828_76601973_2
ARTHROPODA_Candidate_8917_22128931_1
ARTHROPODA_Candidate_51231_103687752_3
ARTHROPODA_Candidate_30468_48273097_0
ARTHROPODA_Candidate_41838_76601974_0
ARTHROPODA_Candidate_8800_22082472_4
ARTHROPODA_Candidate_30470_48273098_0
ARTHROPODA_Candidate_41724_76594655_4
ARTHROPODA_Candidate_53220_106183861_4
ARTHROPODA_Candidate_51366_103700074_1
ARTHROPODA_Candidate_53315_106196416_4
ARTHROPODA_Candidate_48391_98422414_2
ARTHROPODA_Candidate_8578_21044041_2
ARTHROPODA_Candidate_11173_28282286_4
ARTHROPODA_Candidate_50428_102405614_1
ARTHROPODA_Candidate_9293_23138448_4
ARTHROPODA_Candidate_53271_106190031_4
ARTHROPODA_Candidate_9317_23143506_1
ARTHROPODA_Candidate_51399_103704263_5
ARTHROPODA_Candidate_30221_47991560_1
ARTHROPODA_Candidate_51335_103696292_0
ARTHROPODA_Candidate_8816_22102014_1
ARTHROPODA_Candidate_53748_106419859_0
ARTHROPODA_Candidate_50886_103230984_0
ARTHROPODA_Candidate_8574_21037400_1
ARTHROPODA_Candidate_50883_103230983_2
ARTHROPODA_Candidate_53726_106412495_3
ARTHROPODA_Candidate_25705_39880406_0
ARTHROPODA_Candidate_25818_39926525_3
ARTHROPODA_Candidate_26032_40778229_1
ARTHROPODA_Candidate_51418_103714923_2
ARTHROPODA_Candidate_25814_39926524_3
ARTHROPODA_Candidate_25708_39880407_2
ARTHROPODA_Candidate_49019_99927307_3
ARTHROPODA_Candidate_25741_39889845_2
ARTHROPODA_Candidate_42519_77767670_1
ARTHROPODA_Candidate_41866_76607508_0
ARTHROPODA_Candidate_51406_103705432_1
ARTHROPODA_Candidate_51409_103705433_2
ARTHROPODA_Candidate_44596_87533849_0
ARTHROPODA_Candidate_44609_87533850_0
ARTHROPODA_Candidate_45870_91559343_3
ARTHROPODA_Candidate_33944_56668066_1
ARTHROPODA_Candidate_41941_76620876_2
ARTHROPODA_Candidate_50006_102234127_1
ARTHROPODA_Candidate_50026_102234128_1
ARTHROPODA_Candidate_27664_42993994_3
ARTHROPODA_Candidate_2951_7184277_2
ARTHROPODA_Candidate_26591_41794450_2
ARTHROPODA_Candidate_41909_76619300_2
ARTHROPODA_Candidate_10961_28082919_2
ARTHROPODA_Candidate_24329_35532039_1
ARTHROPODA_Candidate_41624_76588029_1
ARTHROPODA_Candidate_39357_70174425_1
ARTHROPODA_Candidate_37626_65593501_2
ARTHROPODA_Candidate_29516_45984746_3
ARTHROPODA_Candidate_51645_103753404_2
ARTHROPODA_Candidate_4085_12015234_0
ARTHROPODA_Candidate_9632_24121060_2
ARTHROPODA_Candidate_38214_66352348_0
ARTHROPODA_Candidate_41672_76590955_2
ARTHROPODA_Candidate_38205_66352347_0
ARTHROPODA_Candidate_41661_76590954_1
ARTHROPODA_Candidate_35884_60663725_0
ARTHROPODA_Candidate_30372_48011208_0
ARTHROPODA_Candidate_30359_48011207_0
ARTHROPODA_Candidate_41533_76156986_2
ARTHROPODA_Candidate_42569_77826491_0
ARTHROPODA_Candidate_35927_60668379_0
ARTHROPODA_Candidate_35678_60646021_1
ARTHROPODA_Candidate_35687_60646022_1

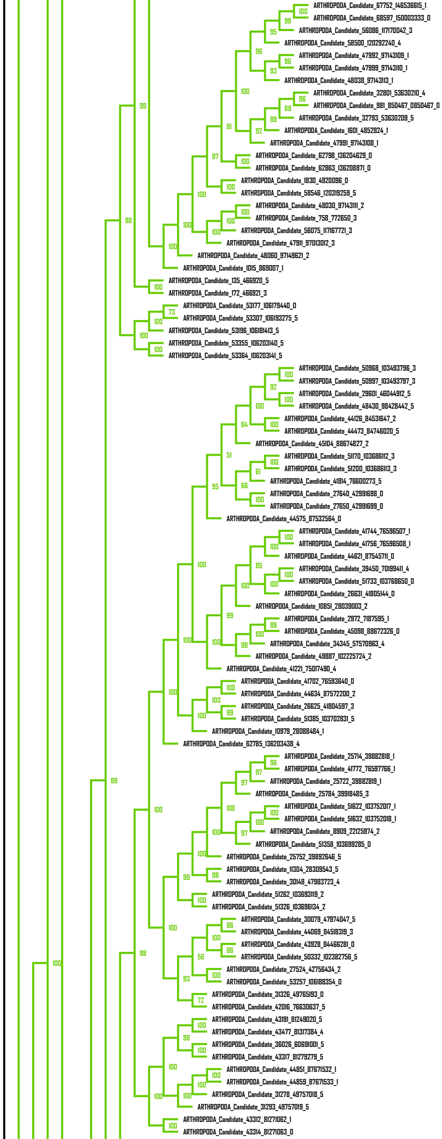
ARTHROPODA_Candidate_67752_146536615_1
ARTHROPODA_Candidate_68597_150003333_0
ARTHROPODA_Candidate_56086_117170042_3
ARTHROPODA_Candidate_58500_120292240_4
ARTHROPODA_Candidate_47992_97143109_1
ARTHROPODA_Candidate_47999_97143110_1
ARTHROPODA_Candidate_48038_97143113_1
ARTHROPODA_Candidate_32801_53630210_4
ARTHROPODA_Candidate_981_850467_0850467_0
ARTHROPODA_Candidate_32793_53630209_5
ARTHROPODA_Candidate_1601_4852824_1
ARTHROPODA_Candidate_47991_97143108_1
ARTHROPODA_Candidate_62798_136204629_0
ARTHROPODA_Candidate_62863_136208871_0
ARTHROPODA_Candidate_1830_4920096_0
ARTHROPODA_Candidate_58546_120319259_5
ARTHROPODA_Candidate_48030_97143111_2
ARTHROPODA_Candidate_758_772650_3
ARTHROPODA_Candidate_56075_117167721_3
ARTHROPODA_Candidate_47911_97013012_3
ARTHROPODA_Candidate_48060_97149621_2
ARTHROPODA_Candidate_1015_869007_1
ARTHROPODA_Candidate_135_466920_5
ARTHROPODA_Candidate_172_466921_3
ARTHROPODA_Candidate_53177_106179440_0
ARTHROPODA_Candidate_53307_106193275_5
ARTHROPODA_Candidate_53196_106181413_5
ARTHROPODA_Candidate_53355_106203140_5
ARTHROPODA_Candidate_53364_106203141_5
ARTHROPODA_Candidate_50968_103493796_3
ARTHROPODA_Candidate_50997_103493797_3
ARTHROPODA_Candidate_29601_46044912_5
ARTHROPODA_Candidate_48430_98428442_5
ARTHROPODA_Candidate_44126_84531647_2
ARTHROPODA_Candidate_44473_84746020_5
ARTHROPODA_Candidate_45104_88674827_2
ARTHROPODA_Candidate_51170_103686112_3
ARTHROPODA_Candidate_51200_103686113_3
ARTHROPODA_Candidate_41814_76600273_5
ARTHROPODA_Candidate_27640_42991698_0
ARTHROPODA_Candidate_27650_42991699_0
ARTHROPODA_Candidate_44575_87532564_0
ARTHROPODA_Candidate_41744_76596507_1
ARTHROPODA_Candidate_41756_76596508_1
ARTHROPODA_Candidate_44621_87545711_0
ARTHROPODA_Candidate_39450_70199411_4
ARTHROPODA_Candidate_51733_103768650_0
ARTHROPODA_Candidate_26631_41805144_0
ARTHROPODA_Candidate_10851_28039003_2
ARTHROPODA_Candidate_2972_7187595_1
ARTHROPODA_Candidate_45098_88672326_0
ARTHROPODA_Candidate_34345_57570963_4
ARTHROPODA_Candidate_49887_102225724_2
ARTHROPODA_Candidate_41221_75017490_4
ARTHROPODA_Candidate_41702_76593640_0
ARTHROPODA_Candidate_44634_87572200_2
ARTHROPODA_Candidate_26625_41804597_3
ARTHROPODA_Candidate_51385_103702831_5
ARTHROPODA_Candidate_10979_28088484_1
ARTHROPODA_Candidate_62785_136203438_4
ARTHROPODA_Candidate_25714_39882818_1
ARTHROPODA_Candidate_41772_76597766_1
ARTHROPODA_Candidate_25722_39882819_1
ARTHROPODA_Candidate_25784_39918485_3
ARTHROPODA_Candidate_51622_103752017_1
ARTHROPODA_Candidate_51632_103752018_1
ARTHROPODA_Candidate_8909_22125974_2
ARTHROPODA_Candidate_51358_103699285_0
ARTHROPODA_Candidate_25752_39892646_5
ARTHROPODA_Candidate_11304_28309543_5
ARTHROPODA_Candidate_30148_47983723_4
ARTHROPODA_Candidate_51262_103693119_2
ARTHROPODA_Candidate_51326_103696134_2
ARTHROPODA_Candidate_30079_47974047_5
ARTHROPODA_Candidate_44069_84518319_3
ARTHROPODA_Candidate_43928_84466281_0
ARTHROPODA_Candidate_50332_102382756_5
ARTHROPODA_Candidate_27524_42756434_2
ARTHROPODA_Candidate_53257_106188354_0
ARTHROPODA_Candidate_31326_49765193_0
ARTHROPODA_Candidate_42016_76630637_5
ARTHROPODA_Candidate_43191_81249020_5
ARTHROPODA_Candidate_43477_81317384_4
ARTHROPODA_Candidate_36026_60691001_5
ARTHROPODA_Candidate_43317_81279279_5
ARTHROPODA_Candidate_44851_87671532_1
ARTHROPODA_Candidate_44859_87671533_1
ARTHROPODA_Candidate_31278_49757018_5
ARTHROPODA_Candidate_31293_49757019_5
ARTHROPODA_Candidate_43312_81271062_1
ARTHROPODA_Candidate_43314_81271063_0

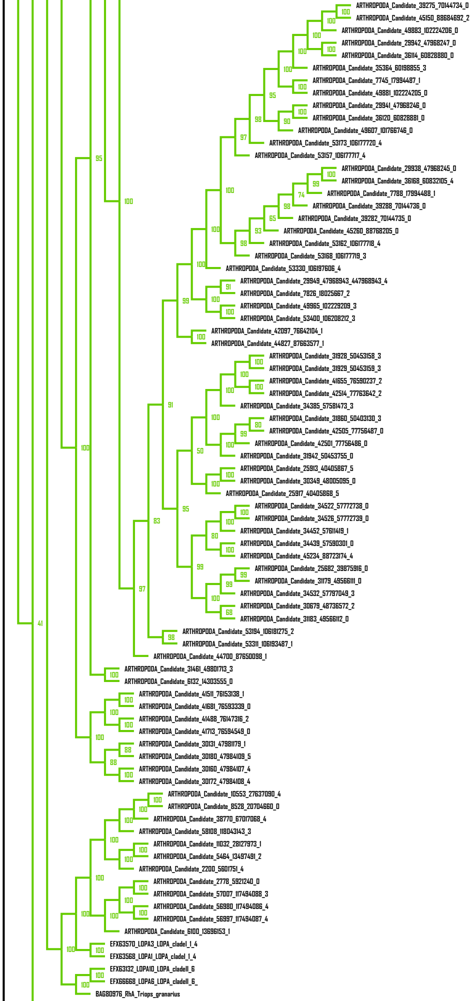
ARTHROPODA_Candidate_39275_70144734_0
ARTHROPODA_Candidate_45150_88684692_2
ARTHROPODA_Candidate_49883_102224206_0
ARTHROPODA_Candidate_29942_47968247_0
ARTHROPODA_Candidate_36114_60828880_0
ARTHROPODA_Candidate_35364_60198855_3
ARTHROPODA_Candidate_7745_17994487_1
ARTHROPODA_Candidate_49881_102224205_0
ARTHROPODA_Candidate_29941_47968246_0
ARTHROPODA_Candidate_36120_60828881_0
ARTHROPODA_Candidate_49607_101766746_0
ARTHROPODA_Candidate_53173_106177720_4
ARTHROPODA_Candidate_53157_106177717_4
ARTHROPODA_Candidate_29938_47968245_0
ARTHROPODA_Candidate_36168_60832105_4
ARTHROPODA_Candidate_7788_17994488_1
ARTHROPODA_Candidate_39288_70144736_0
ARTHROPODA_Candidate_39282_70144735_0
ARTHROPODA_Candidate_45260_88768205_0
ARTHROPODA_Candidate_53162_106177718_4
ARTHROPODA_Candidate_53168_106177719_3
ARTHROPODA_Candidate_53330_106197606_4
ARTHROPODA_Candidate_29949_47968943_447968943_4
ARTHROPODA_Candidate_7826_18025667_2
ARTHROPODA_Candidate_49965_102229209_3
ARTHROPODA_Candidate_53400_106208212_3
ARTHROPODA_Candidate_42097_76642104_1
ARTHROPODA_Candidate_44827_87663577_1
ARTHROPODA_Candidate_31928_50453158_3
ARTHROPODA_Candidate_31929_50453159_3
ARTHROPODA_Candidate_41655_76590237_2
ARTHROPODA_Candidate_42514_77763642_2
ARTHROPODA_Candidate_34385_57581473_3
ARTHROPODA_Candidate_31860_50403130_3
ARTHROPODA_Candidate_42505_77756487_0
ARTHROPODA_Candidate_42501_77756486_0
ARTHROPODA_Candidate_31942_50453755_0
ARTHROPODA_Candidate_25913_40405867_5
ARTHROPODA_Candidate_30349_48005095_0
ARTHROPODA_Candidate_25917_40405868_5
ARTHROPODA_Candidate_34522_57772738_0
ARTHROPODA_Candidate_34526_57772739_0
ARTHROPODA_Candidate_34452_57611419_1
ARTHROPODA_Candidate_34439_57590301_0
ARTHROPODA_Candidate_45234_88723174_4
ARTHROPODA_Candidate_25682_39875916_0
ARTHROPODA_Candidate_31179_49566111_0
ARTHROPODA_Candidate_34532_57797049_3
ARTHROPODA_Candidate_30679_48736572_2
ARTHROPODA_Candidate_31183_49566112_0
ARTHROPODA_Candidate_53194_106181275_2
ARTHROPODA_Candidate_53311_106193487_1
ARTHROPODA_Candidate_44700_87650098_1
ARTHROPODA_Candidate_31461_49801713_3
ARTHROPODA_Candidate_6132_14303555_0
ARTHROPODA_Candidate_41511_76153138_1
ARTHROPODA_Candidate_41681_76593339_0
ARTHROPODA_Candidate_41488_76147316_2
ARTHROPODA_Candidate_41713_76594549_0
ARTHROPODA_Candidate_30131_47981179_1
ARTHROPODA_Candidate_30180_47984109_5
ARTHROPODA_Candidate_30160_47984107_4
ARTHROPODA_Candidate_30172_47984108_4
ARTHROPODA_Candidate_10553_27637090_4
ARTHROPODA_Candidate_8528_20704660_0
ARTHROPODA_Candidate_38770_67017068_4
ARTHROPODA_Candidate_58108_118043143_3
ARTHROPODA_Candidate_11032_28127973_1
ARTHROPODA_Candidate_5464_13497491_2
ARTHROPODA_Candidate_2200_5601751_4
ARTHROPODA_Candidate_2778_5921240_0
ARTHROPODA_Candidate_57007_117494088_3
ARTHROPODA_Candidate_56980_117494086_4
ARTHROPODA_Candidate_56997_117494087_4
ARTHROPODA_Candidate_6100_13696153_1
EFX63570_LOPA3_LOPA_cladeI_1_4
EFX63568_LOPA1_LOPA_cladeI_1_4
EFX63132_LOPA10_LOPA_cladeII_6
EFX66668_LOPA6_LOPA_cladeII_6_
BAG80976_RhA_Triops_granarius

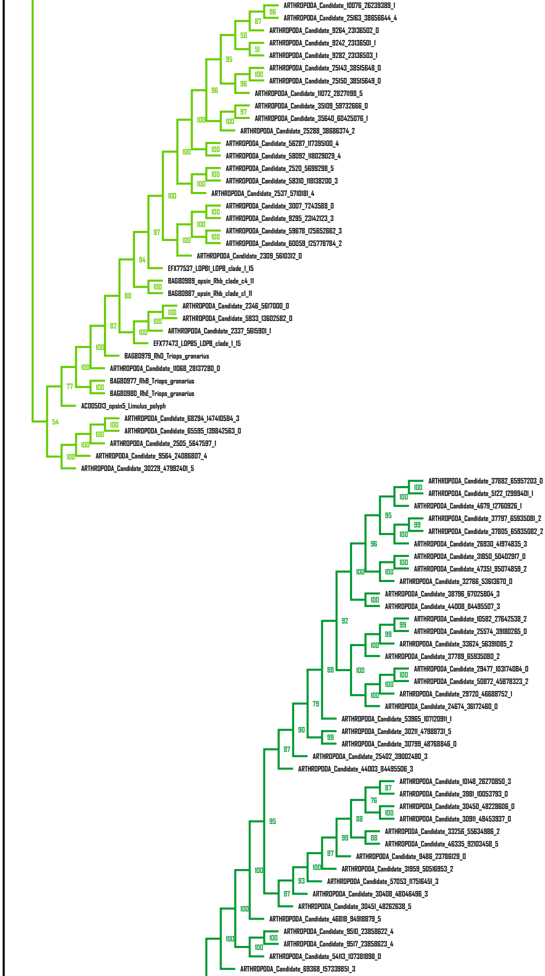
ARTHROPODA_Candidate_10076_26239389_1
ARTHROPODA_Candidate_25163_38656644_4
ARTHROPODA_Candidate_9264_23136502_0
ARTHROPODA_Candidate_9242_23136501_1
ARTHROPODA_Candidate_9282_23136503_1
ARTHROPODA_Candidate_25143_38515648_0
ARTHROPODA_Candidate_25150_38515649_0
ARTHROPODA_Candidate_11072_28271198_5
ARTHROPODA_Candidate_35109_59732666_0
ARTHROPODA_Candidate_35640_60425076_1
ARTHROPODA_Candidate_25288_38686374_2
ARTHROPODA_Candidate_56287_117395100_4
ARTHROPODA_Candidate_58092_118029029_4
ARTHROPODA_Candidate_2520_5699298_5
ARTHROPODA_Candidate_58310_118138200_3
ARTHROPODA_Candidate_2537_5710181_4
ARTHROPODA_Candidate_3007_7243588_0
ARTHROPODA_Candidate_9295_23142123_3
ARTHROPODA_Candidate_59678_125652662_3
ARTHROPODA_Candidate_60059_125778784_2
ARTHROPODA_Candidate_2309_5610312_0
EFX77537_LOPB1_LOPB_clade_1_15
BAG80989_opsin_Rhb_clade_c4_11
BAG80987_opsin_Rhb_clade_c1_11
ARTHROPODA_Candidate_2346_5617000_0
ARTHROPODA_Candidate_5933_13602582_0
ARTHROPODA_Candidate_2337_5615901_1
EFX77473_LOPB5_LOPB_clade_1_15
BAG80979_RhD_Triops_granarius
ARTHROPODA_Candidate_11068_28137280_0
BAG80977_RhB_Triops_granarius
BAG80980_RhE_Triops_granarius
AC005013_opsin5_Limulus_polyph
ARTHROPODA_Candidate_68294_147410584_3
ARTHROPODA_Candidate_65595_139842563_0
ARTHROPODA_Candidate_2505_5647597_1
ARTHROPODA_Candidate_9564_24086807_4
ARTHROPODA_Candidate_30229_47992401_5
ARTHROPODA_Candidate_37882_65957203_0
ARTHROPODA_Candidate_5122_12999401_1
ARTHROPODA_Candidate_4679_12760926_1
ARTHROPODA_Candidate_37797_65935081_2
ARTHROPODA_Candidate_37805_65935082_2
ARTHROPODA_Candidate_26930_41974835_3
ARTHROPODA_Candidate_31850_50402917_0
ARTHROPODA_Candidate_47351_95074859_2
ARTHROPODA_Candidate_32766_53613670_0
ARTHROPODA_Candidate_38796_67025804_3
ARTHROPODA_Candidate_44008_84495507_3
ARTHROPODA_Candidate_10582_27642538_2
ARTHROPODA_Candidate_25574_39180265_0
ARTHROPODA_Candidate_33624_56391085_2
ARTHROPODA_Candidate_37789_65935080_2
ARTHROPODA_Candidate_29477_103174084_0
ARTHROPODA_Candidate_50872_45878323_2
ARTHROPODA_Candidate_29720_46688752_1
ARTHROPODA_Candidate_24674_36172460_0
ARTHROPODA_Candidate_53965_107120911_1
ARTHROPODA_Candidate_30211_47988731_5
ARTHROPODA_Candidate_30799_48768846_0
ARTHROPODA_Candidate_25402_39002480_3
ARTHROPODA_Candidate_44003_84495506_3
ARTHROPODA_Candidate_10148_26270850_3
ARTHROPODA_Candidate_3981_10053793_0
ARTHROPODA_Candidate_30450_48228606_0
ARTHROPODA_Candidate_30911_49453937_0
ARTHROPODA_Candidate_33256_55634986_2
ARTHROPODA_Candidate_46335_92103458_5
ARTHROPODA_Candidate_9486_23786129_0
ARTHROPODA_Candidate_31959_50516953_2
ARTHROPODA_Candidate_57053_117516451_3
ARTHROPODA_Candidate_30408_48046496_3
ARTHROPODA_Candidate_30451_48262638_5
ARTHROPODA_Candidate_46818_94918879_5
ARTHROPODA_Candidate_9510_23858622_4
ARTHROPODA_Candidate_9517_23858623_4
ARTHROPODA_Candidate_54113_107381898_0
ARTHROPODA_Candidate_69368_157339851_3

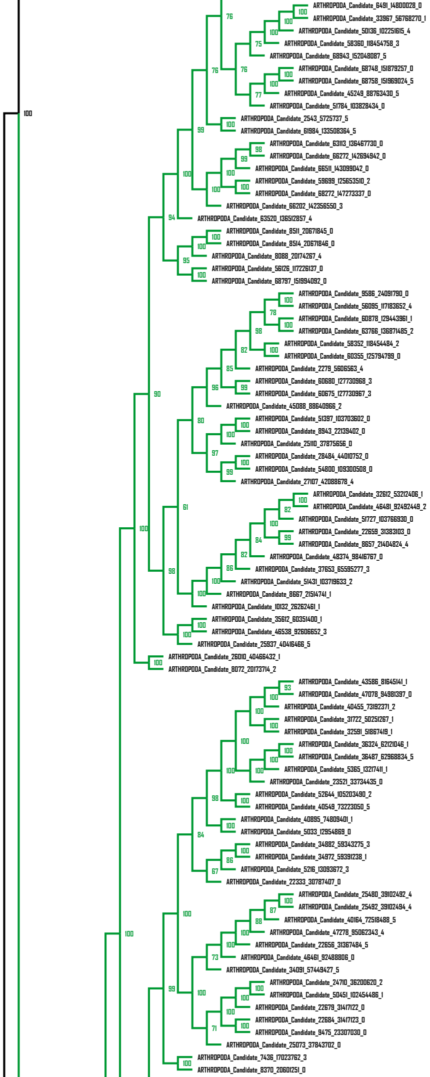
ARTHROPODA_Candidate_6491_14800028_0
ARTHROPODA_Candidate_33967_56768270_1
ARTHROPODA_Candidate_50136_102251615_4
ARTHROPODA_Candidate_58360_118454758_3
ARTHROPODA_Candidate_68943_152048087_5
ARTHROPODA_Candidate_68748_151879257_0
ARTHROPODA_Candidate_68758_151969024_5
ARTHROPODA_Candidate_45249_88763430_5
ARTHROPODA_Candidate_51784_103828434_0
ARTHROPODA_Candidate_2543_5725737_5
ARTHROPODA_Candidate_61984_133508364_5
ARTHROPODA_Candidate_63113_136467730_0
ARTHROPODA_Candidate_66272_142694942_0
ARTHROPODA_Candidate_66511_143099042_0
ARTHROPODA_Candidate_59699_125653510_2
ARTHROPODA_Candidate_68272_147273337_0
ARTHROPODA_Candidate_66202_142356550_3
ARTHROPODA_Candidate_63520_136512857_4
ARTHROPODA_Candidate_8511_20671845_0
ARTHROPODA_Candidate_8514_20671846_0
ARTHROPODA_Candidate_8088_20174267_4
ARTHROPODA_Candidate_56126_117226137_0
ARTHROPODA_Candidate_68797_151994092_0
ARTHROPODA_Candidate_9586_24091790_0
ARTHROPODA_Candidate_56095_117183652_4
ARTHROPODA_Candidate_60878_129443961_1
ARTHROPODA_Candidate_63766_136871485_2
ARTHROPODA_Candidate_58352_118454484_2
ARTHROPODA_Candidate_60355_125794799_0
ARTHROPODA_Candidate_2279_5606563_4
ARTHROPODA_Candidate_60680_127730968_3
ARTHROPODA_Candidate_60675_127730967_3
ARTHROPODA_Candidate_45088_88640966_2
ARTHROPODA_Candidate_51397_103703602_0
ARTHROPODA_Candidate_8943_22139402_0
ARTHROPODA_Candidate_25110_37875656_0
ARTHROPODA_Candidate_28484_44010752_0
ARTHROPODA_Candidate_54800_109300508_0
ARTHROPODA_Candidate_27107_42088678_4
ARTHROPODA_Candidate_32612_53212406_1
ARTHROPODA_Candidate_46481_92492449_2
ARTHROPODA_Candidate_51727_103766930_0
ARTHROPODA_Candidate_22659_31383103_0
ARTHROPODA_Candidate_8657_21404824_4
ARTHROPODA_Candidate_48374_98416767_0
ARTHROPODA_Candidate_37653_65595277_3
ARTHROPODA_Candidate_51431_103719633_2
ARTHROPODA_Candidate_8667_21514741_1
ARTHROPODA_Candidate_10132_26262461_1
ARTHROPODA_Candidate_35612_60351400_1
ARTHROPODA_Candidate_46538_92606652_3
ARTHROPODA_Candidate_25937_40416466_5
ARTHROPODA_Candidate_26010_40466432_1
ARTHROPODA_Candidate_8072_20173714_2
ARTHROPODA_Candidate_43586_81645141_1
ARTHROPODA_Candidate_47078_94981397_0
ARTHROPODA_Candidate_40455_73192371_2
ARTHROPODA_Candidate_31722_50251267_1
ARTHROPODA_Candidate_32591_51867419_1
ARTHROPODA_Candidate_36324_62121046_1
ARTHROPODA_Candidate_36487_62968834_5
ARTHROPODA_Candidate_5365_13217411_1
ARTHROPODA_Candidate_23521_33734435_0
ARTHROPODA_Candidate_52644_105203490_2
ARTHROPODA_Candidate_40549_73223050_5
ARTHROPODA_Candidate_40895_74809401_1
ARTHROPODA_Candidate_5033_12954869_0
ARTHROPODA_Candidate_34882_59343275_3
ARTHROPODA_Candidate_34972_59391238_1
ARTHROPODA_Candidate_5216_13093672_3
ARTHROPODA_Candidate_22333_30787407_0
ARTHROPODA_Candidate_25480_39102492_4
ARTHROPODA_Candidate_25492_39102494_4
ARTHROPODA_Candidate_40164_72518488_5
ARTHROPODA_Candidate_47278_95062343_4
ARTHROPODA_Candidate_22656_31367484_5
ARTHROPODA_Candidate_46461_92488806_0
ARTHROPODA_Candidate_34091_57449427_5
ARTHROPODA_Candidate_24710_36200620_2
ARTHROPODA_Candidate_50451_102454486_1
ARTHROPODA_Candidate_22679_31417122_0
ARTHROPODA_Candidate_22684_31417123_0
ARTHROPODA_Candidate_9475_23307030_0
ARTHROPODA_Candidate_25073_37843702_0
ARTHROPODA_Candidate_7436_17023762_3
ARTHROPODA_Candidate_8370_20601251_0

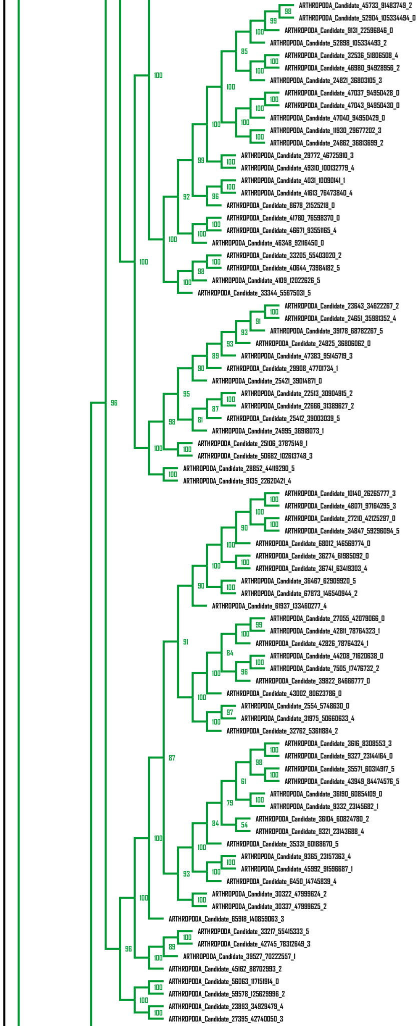
ARTHROPODA_Candidate_45733_91483749_2
ARTHROPODA_Candidate_52904_105334494_0
ARTHROPODA_Candidate_9131_22596846_0
ARTHROPODA_Candidate_52898_105334493_2
ARTHROPODA_Candidate_32536_51806508_4
ARTHROPODA_Candidate_46980_94928956_2
ARTHROPODA_Candidate_24821_36803105_3
ARTHROPODA_Candidate_47037_94950428_0
ARTHROPODA_Candidate_47043_94950430_0
ARTHROPODA_Candidate_47040_94950429_0
ARTHROPODA_Candidate_11930_29677202_3
ARTHROPODA_Candidate_24862_36813699_2
ARTHROPODA_Candidate_29772_46725910_3
ARTHROPODA_Candidate_49310_100132779_4
ARTHROPODA_Candidate_4031_10090141_1
ARTHROPODA_Candidate_41613_76473840_4
ARTHROPODA_Candidate_8678_21525218_0
ARTHROPODA_Candidate_41780_76598370_0
ARTHROPODA_Candidate_46671_93551165_4
ARTHROPODA_Candidate_46348_92116450_0
ARTHROPODA_Candidate_33205_55403020_2
ARTHROPODA_Candidate_40644_73984182_5
ARTHROPODA_Candidate_4109_12022626_5
ARTHROPODA_Candidate_33344_55675031_5
ARTHROPODA_Candidate_23643_34622267_2
ARTHROPODA_Candidate_24651_35981352_4
ARTHROPODA_Candidate_39178_68782267_5
ARTHROPODA_Candidate_24825_36806062_0
ARTHROPODA_Candidate_47383_95145719_3
ARTHROPODA_Candidate_29908_47701734_1
ARTHROPODA_Candidate_25421_39014871_0
ARTHROPODA_Candidate_22513_30904915_2
ARTHROPODA_Candidate_22666_31389627_2
ARTHROPODA_Candidate_25412_39003039_5
ARTHROPODA_Candidate_24995_36918073_1
ARTHROPODA_Candidate_25106_37875149_1
ARTHROPODA_Candidate_50682_102613748_3
ARTHROPODA_Candidate_28852_44119290_5
ARTHROPODA_Candidate_9135_22620421_4
ARTHROPODA_Candidate_10140_26265777_3
ARTHROPODA_Candidate_48071_97164295_3
ARTHROPODA_Candidate_27210_42125297_0
ARTHROPODA_Candidate_34847_59296094_5
ARTHROPODA_Candidate_68012_146569774_0
ARTHROPODA_Candidate_36274_61985092_0
ARTHROPODA_Candidate_36741_63419303_4
ARTHROPODA_Candidate_36467_62909920_5
ARTHROPODA_Candidate_67873_146540944_2
ARTHROPODA_Candidate_61937_133460277_4
ARTHROPODA_Candidate_27055_42079066_0
ARTHROPODA_Candidate_42811_78764323_1
ARTHROPODA_Candidate_42826_78764324_1
ARTHROPODA_Candidate_44208_71620638_0
ARTHROPODA_Candidate_7505_17476732_2
ARTHROPODA_Candidate_39822_84666777_0
ARTHROPODA_Candidate_43002_80623786_0
ARTHROPODA_Candidate_2554_5748630_0
ARTHROPODA_Candidate_31975_50660633_4
ARTHROPODA_Candidate_32762_53611884_2
ARTHROPODA_Candidate_3616_8308553_3
ARTHROPODA_Candidate_9327_23144164_0
ARTHROPODA_Candidate_35571_60314917_5
ARTHROPODA_Candidate_43949_84474576_5
ARTHROPODA_Candidate_36190_60854109_0
ARTHROPODA_Candidate_9332_23145682_1
ARTHROPODA_Candidate_36104_60824780_2
ARTHROPODA_Candidate_9321_23143688_4
ARTHROPODA_Candidate_35331_60188670_5
ARTHROPODA_Candidate_9365_23157363_4
ARTHROPODA_Candidate_45992_91596687_1
ARTHROPODA_Candidate_6450_14745839_4
ARTHROPODA_Candidate_30322_47999624_2
ARTHROPODA_Candidate_30337_47999625_2
ARTHROPODA_Candidate_65918_140859063_3
ARTHROPODA_Candidate_33217_55415333_5
ARTHROPODA_Candidate_42745_78312649_3
ARTHROPODA_Candidate_39527_70222557_1
ARTHROPODA_Candidate_45162_88702993_2
ARTHROPODA_Candidate_56063_117151914_0
ARTHROPODA_Candidate_59578_125629996_2
ARTHROPODA_Candidate_23893_34929479_4
ARTHROPODA_Candidate_27395_42740050_3

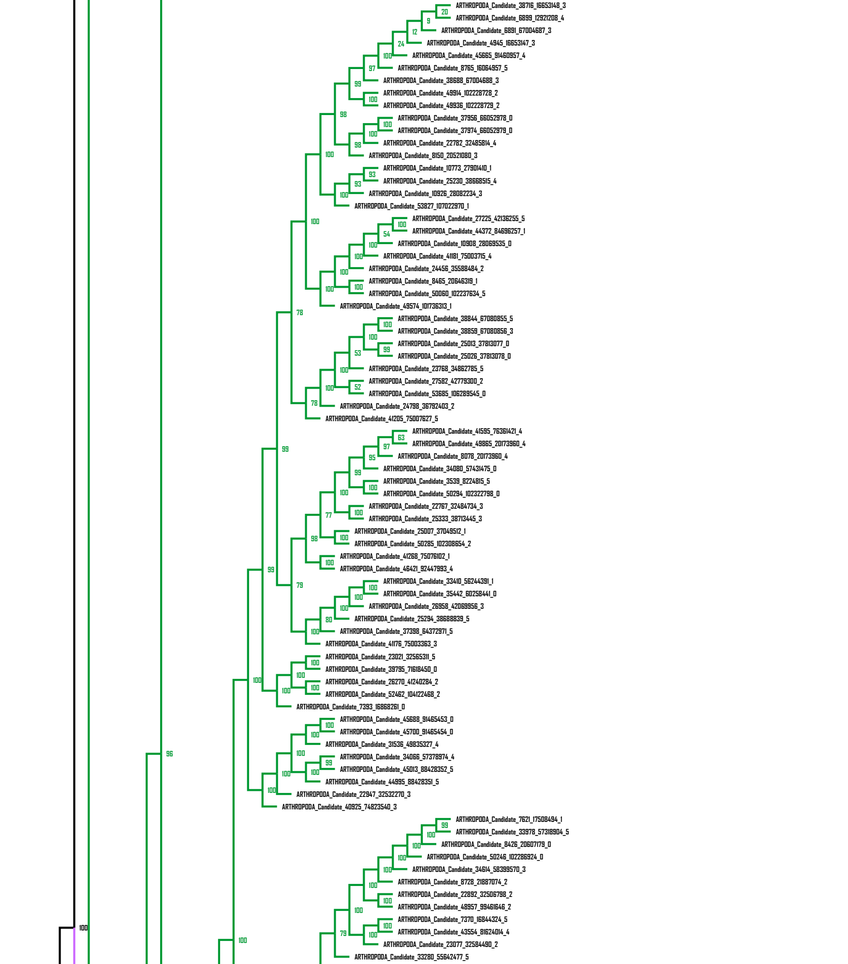

ARTHROPODA_Candidate_38716_16653148_3
ARTHROPODA_Candidate_6899_12921208_4
ARTHROPODA_Candidate_6891_67004687_3
ARTHROPODA_Candidate_4945_16653147_3
ARTHROPODA_Candidate_45665_91460957_4
ARTHROPODA_Candidate_8765_16064957_5
ARTHROPODA_Candidate_38688_67004688_3
ARTHROPODA_Candidate_49914_102228728_2
ARTHROPODA_Candidate_49936_102228729_2
ARTHROPODA_Candidate_37956_66052978_0
ARTHROPODA_Candidate_37974_66052979_0
ARTHROPODA_Candidate_22782_32485814_4
ARTHROPODA_Candidate_8150_20521080_3
ARTHROPODA_Candidate_10773_27901410_1
ARTHROPODA_Candidate_25230_38668515_4
ARTHROPODA_Candidate_10926_28082234_3
ARTHROPODA_Candidate_53827_107022970_1
ARTHROPODA_Candidate_27225_42136255_5
ARTHROPODA_Candidate_44372_84696257_1
ARTHROPODA_Candidate_10908_28069535_0
ARTHROPODA_Candidate_41181_75003715_4
ARTHROPODA_Candidate_24456_35588484_2
ARTHROPODA_Candidate_8465_20646319_1
ARTHROPODA_Candidate_50060_102237634_5
ARTHROPODA_Candidate_49574_101736313_1
ARTHROPODA_Candidate_38844_67080855_5
ARTHROPODA_Candidate_38859_67080856_3
ARTHROPODA_Candidate_25013_37813077_0
ARTHROPODA_Candidate_25026_37813078_0
ARTHROPODA_Candidate_23768_34862785_5
ARTHROPODA_Candidate_27582_42779300_2
ARTHROPODA_Candidate_53685_106289545_0
ARTHROPODA_Candidate_24798_36792403_2
ARTHROPODA_Candidate_41205_75007627_5
ARTHROPODA_Candidate_41595_76361421_4
ARTHROPODA_Candidate_49865_20173960_4
ARTHROPODA_Candidate_8078_20173960_4
ARTHROPODA_Candidate_34080_57431475_0
ARTHROPODA_Candidate_3539_8224815_5
ARTHROPODA_Candidate_50294_102322798_0
ARTHROPODA_Candidate_22767_32484734_3
ARTHROPODA_Candidate_25333_38713445_3
ARTHROPODA_Candidate_25007_37049512_1
ARTHROPODA_Candidate_50285_102308654_2
ARTHROPODA_Candidate_41268_75076102_1
ARTHROPODA_Candidate_46421_92447993_4
ARTHROPODA_Candidate_33410_56244391_1
ARTHROPODA_Candidate_35442_60258441_0
ARTHROPODA_Candidate_26958_42069956_3
ARTHROPODA_Candidate_25294_38688839_5
ARTHROPODA_Candidate_37398_64372971_5
ARTHROPODA_Candidate_41176_75003363_3
ARTHROPODA_Candidate_23021_32565311_5
ARTHROPODA_Candidate_39795_71618450_0
ARTHROPODA_Candidate_26270_41240284_2
ARTHROPODA_Candidate_52462_104122468_2
ARTHROPODA_Candidate_7393_16868261_0
ARTHROPODA_Candidate_45688_91465453_0
ARTHROPODA_Candidate_45700_91465454_0
ARTHROPODA_Candidate_31536_49835327_4
ARTHROPODA_Candidate_34066_57378974_4
ARTHROPODA_Candidate_45013_88428352_5
ARTHROPODA_Candidate_44995_88428351_5
ARTHROPODA_Candidate_22947_32532270_3
ARTHROPODA_Candidate_40925_74823540_3
ARTHROPODA_Candidate_7621_17508494_1
ARTHROPODA_Candidate_33978_57318904_5
ARTHROPODA_Candidate_8426_20607179_0
ARTHROPODA_Candidate_50246_102286924_0
ARTHROPODA_Candidate_34614_58399570_3
ARTHROPODA_Candidate_8728_21887074_2
ARTHROPODA_Candidate_22892_32506798_2
ARTHROPODA_Candidate_48957_99461646_2
ARTHROPODA_Candidate_7370_16844324_5
ARTHROPODA_Candidate_43554_81624014_4
ARTHROPODA_Candidate_23077_32584490_2
ARTHROPODA_Candidate_33280_55642477_5

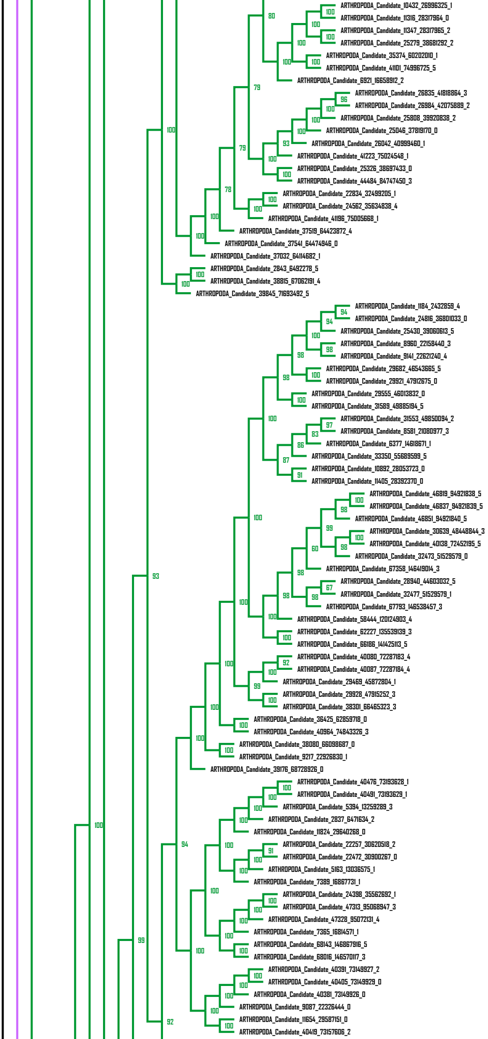

ARTHROPODA_Candidate_10432_26996325_1
ARTHROPODA_Candidate_11316_28317964_0
ARTHROPODA_Candidate_11347_28317965_2
ARTHROPODA_Candidate_25279_38681292_2
ARTHROPODA_Candidate_35374_60202010_1
ARTHROPODA_Candidate_41101_74996725_5
ARTHROPODA_Candidate_6921_16658912_2
ARTHROPODA_Candidate_26835_41818864_3
ARTHROPODA_Candidate_26984_42075889_2
ARTHROPODA_Candidate_25808_39920838_2
ARTHROPODA_Candidate_25046_37819170_0
ARTHROPODA_Candidate_26042_40999460_1
ARTHROPODA_Candidate_41223_75024548_1
ARTHROPODA_Candidate_25326_38697433_0
ARTHROPODA_Candidate_44484_84747450_3
ARTHROPODA_Candidate_22834_32499205_1
ARTHROPODA_Candidate_24562_35634838_4
ARTHROPODA_Candidate_41196_75005668_1
ARTHROPODA_Candidate_37519_64423872_4
ARTHROPODA_Candidate_37541_64474946_0
ARTHROPODA_Candidate_37032_64114682_1
ARTHROPODA_Candidate_2843_6492278_5
ARTHROPODA_Candidate_38815_67062191_4
ARTHROPODA_Candidate_39845_71693492_5
ARTHROPODA_Candidate_1184_2432859_4
ARTHROPODA_Candidate_24816_36801033_0
ARTHROPODA_Candidate_25430_39060613_5
ARTHROPODA_Candidate_8960_22158440_3
ARTHROPODA_Candidate_9141_22621240_4
ARTHROPODA_Candidate_29682_46543665_5
ARTHROPODA_Candidate_29921_47912675_0
ARTHROPODA_Candidate_29555_46013832_0
ARTHROPODA_Candidate_31589_49885194_5
ARTHROPODA_Candidate_31553_49850094_2
ARTHROPODA_Candidate_8581_21080977_3
ARTHROPODA_Candidate_6377_14618671_1
ARTHROPODA_Candidate_33350_55689599_5
ARTHROPODA_Candidate_10892_28053723_0
ARTHROPODA_Candidate_11405_28392370_0
ARTHROPODA_Candidate_46819_94921838_5
ARTHROPODA_Candidate_46837_94921839_5
ARTHROPODA_Candidate_46851_94921840_5
ARTHROPODA_Candidate_30639_48448844_3
ARTHROPODA_Candidate_40138_72452195_5
ARTHROPODA_Candidate_32473_51529579_0
ARTHROPODA_Candidate_67358_146419014_3
ARTHROPODA_Candidate_28940_44603032_5
ARTHROPODA_Candidate_32477_51529579_1
ARTHROPODA_Candidate_67793_146538457_3
ARTHROPODA_Candidate_58444_120124903_4
ARTHROPODA_Candidate_62227_135539139_3
ARTHROPODA_Candidate_66186_141425113_5
ARTHROPODA_Candidate_40080_72287183_4
ARTHROPODA_Candidate_40087_72287184_4
ARTHROPODA_Candidate_29469_45872804_1
ARTHROPODA_Candidate_29928_47915252_3
ARTHROPODA_Candidate_38301_66465323_3
ARTHROPODA_Candidate_36425_62859718_0
ARTHROPODA_Candidate_40964_74843326_3
ARTHROPODA_Candidate_38080_66098687_0
ARTHROPODA_Candidate_9217_22926830_1
ARTHROPODA_Candidate_39176_68728926_0
ARTHROPODA_Candidate_40476_73193628_1
ARTHROPODA_Candidate_40491_73193629_1
ARTHROPODA_Candidate_5394_13259289_3
ARTHROPODA_Candidate_2837_6471634_2
ARTHROPODA_Candidate_11824_29640268_0
ARTHROPODA_Candidate_22257_30620518_2
ARTHROPODA_Candidate_22472_30900267_0
ARTHROPODA_Candidate_5163_13036575_1
ARTHROPODA_Candidate_7389_16867731_1
ARTHROPODA_Candidate_24398_35562692_1
ARTHROPODA_Candidate_47313_95068947_3
ARTHROPODA_Candidate_47328_95072131_4
ARTHROPODA_Candidate_7365_16814571_1
ARTHROPODA_Candidate_68143_146867916_5
ARTHROPODA_Candidate_68016_146570117_3
ARTHROPODA_Candidate_40391_73149927_2
ARTHROPODA_Candidate_40405_73149929_0
ARTHROPODA_Candidate_40381_73149926_0
ARTHROPODA_Candidate_9087_22326444_0
ARTHROPODA_Candidate_11654_29587151_0
ARTHROPODA_Candidate_40419_73157606_2

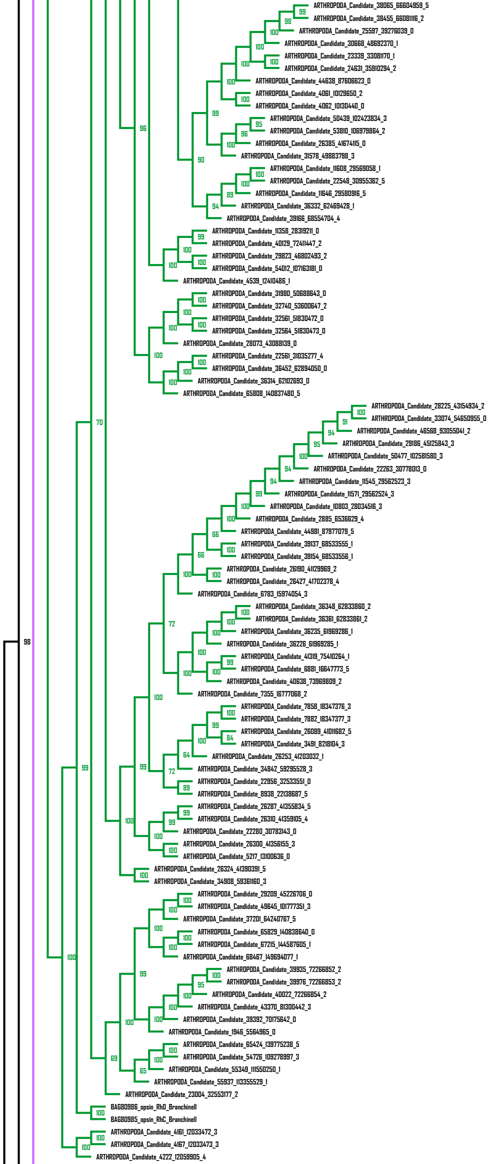
ARTHROPODA_Candidate_38065_66604959_5
ARTHROPODA_Candidate_38455_66081116_2
ARTHROPODA_Candidate_25597_39276039_0
ARTHROPODA_Candidate_30668_48692370_1
ARTHROPODA_Candidate_23339_33081170_1
ARTHROPODA_Candidate_24631_35910294_2
ARTHROPODA_Candidate_44638_87606623_0
ARTHROPODA_Candidate_4061_10129650_2
ARTHROPODA_Candidate_4062_10130440_0
ARTHROPODA_Candidate_50439_102423834_3
ARTHROPODA_Candidate_53810_106979864_2
ARTHROPODA_Candidate_26385_41674115_0
ARTHROPODA_Candidate_31578_49883798_3
ARTHROPODA_Candidate_11608_29569058_1
ARTHROPODA_Candidate_22548_30955362_5
ARTHROPODA_Candidate_11646_29580916_5
ARTHROPODA_Candidate_36332_62469428_1
ARTHROPODA_Candidate_39166_68554704_4
ARTHROPODA_Candidate_11358_28319211_0
ARTHROPODA_Candidate_40129_72411447_2
ARTHROPODA_Candidate_29823_46802493_2
ARTHROPODA_Candidate_54012_107163181_0
ARTHROPODA_Candidate_4539_12410486_1
ARTHROPODA_Candidate_31980_50688643_0
ARTHROPODA_Candidate_32740_53600647_2
ARTHROPODA_Candidate_32561_51830472_0
ARTHROPODA_Candidate_32564_51830473_0
ARTHROPODA_Candidate_28073_43088139_0
ARTHROPODA_Candidate_22561_31035277_4
ARTHROPODA_Candidate_36452_62894050_0
ARTHROPODA_Candidate_36314_62102693_0
ARTHROPODA_Candidate_65808_140837480_5
ARTHROPODA_Candidate_28225_43154934_2
ARTHROPODA_Candidate_33074_54650955_0
ARTHROPODA_Candidate_46568_93055041_2
ARTHROPODA_Candidate_29186_45125843_3
ARTHROPODA_Candidate_50477_102581580_3
ARTHROPODA_Candidate_22263_30778013_0
ARTHROPODA_Candidate_11545_29562523_3
ARTHROPODA_Candidate_11571_29562524_3
ARTHROPODA_Candidate_10803_28034516_3
ARTHROPODA_Candidate_2885_6536629_4
ARTHROPODA_Candidate_44981_87977079_5
ARTHROPODA_Candidate_39137_68533555_1
ARTHROPODA_Candidate_39154_68533556_1
ARTHROPODA_Candidate_26190_41129969_2
ARTHROPODA_Candidate_26427_41702378_4
ARTHROPODA_Candidate_6783_15974054_3
ARTHROPODA_Candidate_36348_62833860_2
ARTHROPODA_Candidate_36361_62833861_2
ARTHROPODA_Candidate_36235_61969286_1
ARTHROPODA_Candidate_36226_61969285_1
ARTHROPODA_Candidate_41319_75410264_1
ARTHROPODA_Candidate_6881_16647773_5
ARTHROPODA_Candidate_40638_73969809_2
ARTHROPODA_Candidate_7355_16777068_2
ARTHROPODA_Candidate_7858_18347376_3
ARTHROPODA_Candidate_7882_18347377_3
ARTHROPODA_Candidate_26089_41011682_5
ARTHROPODA_Candidate_3491_8218104_3
ARTHROPODA_Candidate_26253_41203032_1
ARTHROPODA_Candidate_34842_59295528_3
ARTHROPODA_Candidate_22956_32533551_0
ARTHROPODA_Candidate_8938_22138687_5
ARTHROPODA_Candidate_26287_41355834_5
ARTHROPODA_Candidate_26310_41359105_4
ARTHROPODA_Candidate_22280_30783143_0
ARTHROPODA_Candidate_26300_41356155_3
ARTHROPODA_Candidate_5217_13100636_0
ARTHROPODA_Candidate_26324_41390391_5
ARTHROPODA_Candidate_34908_59361160_3
ARTHROPODA_Candidate_29209_45226706_0
ARTHROPODA_Candidate_49645_101777351_3
ARTHROPODA_Candidate_37201_64240767_5
ARTHROPODA_Candidate_65829_140838640_0
ARTHROPODA_Candidate_67215_144587605_1
ARTHROPODA_Candidate_68467_149694077_1
ARTHROPODA_Candidate_39935_72266852_2
ARTHROPODA_Candidate_39976_72266853_2
ARTHROPODA_Candidate_40022_72266854_2
ARTHROPODA_Candidate_43370_81300442_3
ARTHROPODA_Candidate_39392_70175642_0
ARTHROPODA_Candidate_1946_5564965_0
ARTHROPODA_Candidate_65424_139775238_5
ARTHROPODA_Candidate_54726_109278997_3
ARTHROPODA_Candidate_55349_111550250_1
ARTHROPODA_Candidate_55937_113355529_1
ARTHROPODA_Candidate_23004_32553177_2
BAG80986_opsin_RhD_Branchinell
BAG80985_opsin_RhC_Branchinell
ARTHROPODA_Candidate_4161_12033472_3
ARTHROPODA_Candidate_4167_12033473_3
ARTHROPODA_Candidate_4222_12059905_4

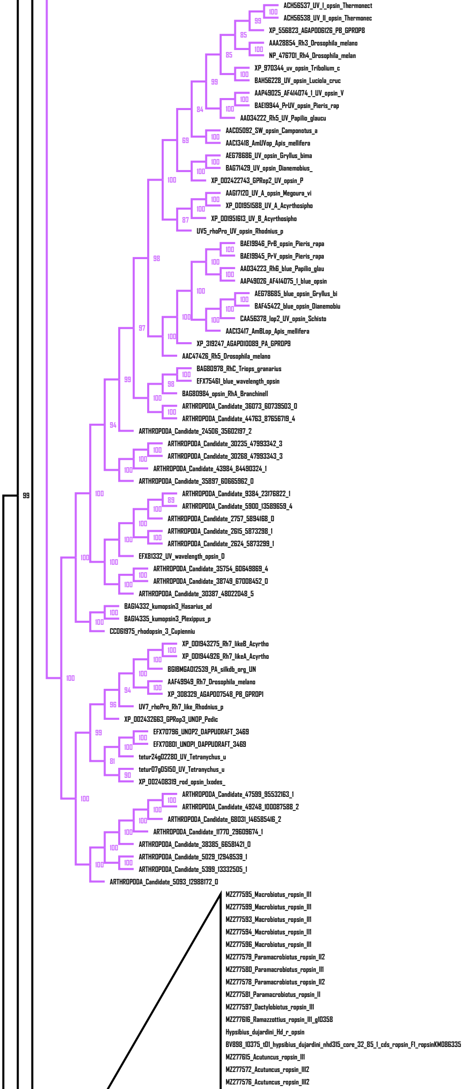
ACH56537_UV_I_opsin_Thermonect
ACH56538_UV_II_opsin_Thermonec
XP_556823_AGAP006126_PB_GPROP8
AAA28854_Rh3_Drosophila_melano
NP_476701_Rh4_Drosophila_melan
XP_970344_uv_opsin_Tribolium_c
BAH56228_UV_opsin_Luciola_cruc
AAP49025_AF414074_1_UV_opsin_V
BAE19944_PrUV_opsin_Pieris_rap
AAD34222_Rh5_UV_Papilio_glaucu
AAC05092_SW_opsin_Camponotus_a
AAC13418_AmUVop_Apis_mellifera
AEG78686_UV_opsin_Gryllus_bima
BAG71429_UV_opsin_Dianemobius_
XP_002422743_GPRop2_UV_opsin_P
AAG17120_UV_A_opsin_Megoura_vi
XP_001951588_UV_A_Acyrthosipho
XP_001951613_UV_B_Acyrthosipho
UV5_rhoPro_UV_opsin_Rhodnius_p
BAE19946_PrB_opsin_Pieris_rapa
BAE19945_PrV_opsin_Pieris_rapa
AAD34223_Rh6_blue_Papilio_glau
AAP49026_AF414075_1_blue_opsin
AEG78685_blue_opsin_Gryllus_bi
BAF45422_blue_opsin_Dianemobiu
CAA56378_lop2_UV_opsin_Schisto
AAC13417_AmBLop_Apis_mellifera
XP_319247_AGAP010089_PA_GPROP9
AAC47426_Rh5_Drosophila_melano
BAG80978_RhC_Triops_granarius
EFX75461_blue_wavelength_opsin
BAG80984_opsin_RhA_Branchinell
ARTHROPODA_Candidate_36073_60739503_0
ARTHROPODA_Candidate_44763_87656719_4
ARTHROPODA_Candidate_24506_35602197_2
ARTHROPODA_Candidate_30235_47993342_3
ARTHROPODA_Candidate_30268_47993343_3
ARTHROPODA_Candidate_43984_84490324_1
ARTHROPODA_Candidate_35897_60665962_0
ARTHROPODA_Candidate_9384_23176822_1
ARTHROPODA_Candidate_5900_13589659_4
ARTHROPODA_Candidate_2757_5894168_0
ARTHROPODA_Candidate_2615_5873298_1
ARTHROPODA_Candidate_2624_5873299_1
EFX81332_UV_wavelength_opsin_D
ARTHROPODA_Candidate_35754_60649869_4
ARTHROPODA_Candidate_38749_67008452_0
ARTHROPODA_Candidate_30387_48022048_5
BAG14332_kumopsin3_Hasarius_ad
BAG14335_kumopsin3_Plexippus_p
CCO61975_rhodopsin_3_Cupienniu
XP_001943275_Rh7_likeB_Acyrtho
XP_001944926_Rh7_likeA_Acyrtho
BGIBMGA012539_PA_silkdb_org_UN
AAF49949_Rh7_Drosophila_melano
XP_308329_AGAP007548_PB_GPROP1
UV7_rhoPro_Rh7_like_Rhodnius_p
XP_002432663_GPRop3_UNOP_Pedic
EFX70796_UNOP2_DAPPUDRAFT_3469
EFX70801_UNOP1_DAPPUDRAFT_3469
tetur24g02280_UV_Tetranychus_u
tetur07g05150_UV_Tetranychus_u
XP_002408319_rod_opsin_Ixodes_
ARTHROPODA_Candidate_47599_95532163_1
ARTHROPODA_Candidate_49248_100087588_2
ARTHROPODA_Candidate_68031_146585416_2
ARTHROPODA_Candidate_11770_29609674_1
ARTHROPODA_Candidate_38385_66581421_0
ARTHROPODA_Candidate_5029_12948539_1
ARTHROPODA_Candidate_5399_13332505_1
ARTHROPODA_Candidate_5093_12988172_0
MZ277595_Macrobiotus_ropsin_III
MZ277599_Macrobiotus_ropsin_III
MZ277593_Macrobiotus_ropsin_III
MZ277594_Macrobiotus_ropsin_III
MZ277596_Macrobiotus_ropsin_III
MZ277579_Paramacrobiotus_ropsin_II2
MZ277580_Paramacrobiotus_ropsin_III
MZ277578_Paramacrobiotus_ropsin_II2
MZ277581_Paramacrobiotus_ropsin_II
MZ277597_Dactylobiotus_ropsin_III
MZ277616_Ramazzottius_ropsin_III_g10358
Hypsibius_dujardini_Hd_r_opsin
BV898_10375_t01_hypsibius_dujardini_nhd315_core_32_85_1_cds_ropsin_F1_ropsinKM086335
MZ277615_Acutuncus_ropsin_III
MZ277572_Acutuncus_ropsin_III2
MZ277576_Acutuncus_ropsin_III2

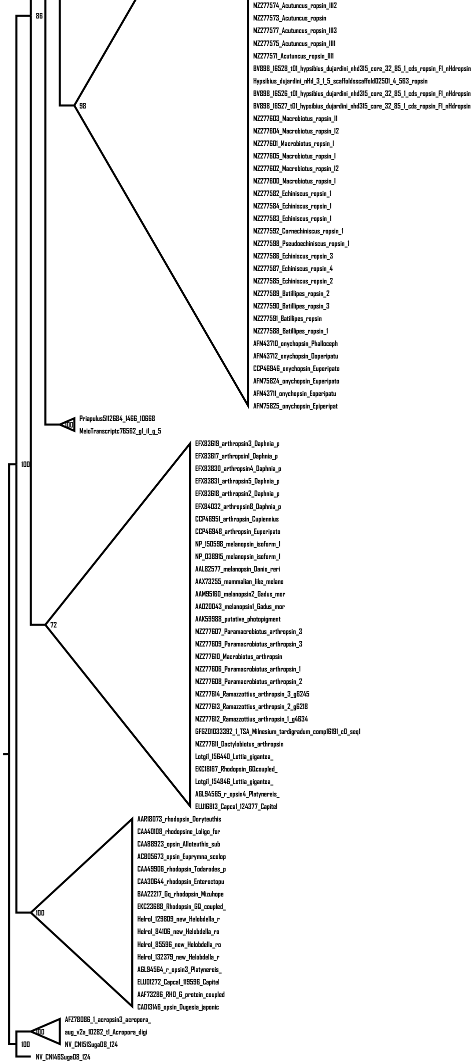

MZ277574_Acutuncus_ropsin_III2
MZ277573_Acutuncus_ropsin
MZ277577_Acutuncus_ropsin_III3
MZ277575_Acutuncus_ropsin_IIII
MZ277571_Acutuncus_ropsin_IIII
BV898_16528_t01_hypsibius_dujardini_nhd315_core_32_85_1_cds_ropsin_F1_nHdropsin
Hypsibius_dujardini_nHd_3_1_5_scaffoldsscaffold02501_4_563_ropsin
BV898_16526_t01_hypsibius_dujardini_nhd315_core_32_85_1_cds_ropsin_F1_nHdropsin
BV898_16527_t01_hypsibius_dujardini_nhd315_core_32_85_1_cds_ropsin_F1_nHdropsin
MZ277603_Macrobiotus_ropsin_I1
MZ277604_Macrobiotus_ropsin_I2
MZ277601_Macrobiotus_ropsin_I
MZ277605_Macrobiotus_ropsin_I
MZ277602_Macrobiotus_ropsin_I2
MZ277600_Macrobiotus_ropsin_I
MZ277582_Echiniscus_ropsin_1
MZ277584_Echiniscus_ropsin_1
MZ277583_Echiniscus_ropsin_1
MZ277592_Cornechiniscus_ropsin_1
MZ277598_Pseudoechiniscus_ropsin_1
MZ277586_Echiniscus_ropsin_3
MZ277587_Echiniscus_ropsin_4
MZ277585_Echiniscus_ropsin_2
MZ277589_Batillipes_ropsin_2
MZ277590_Batillipes_ropsin_3
MZ277591_Batillipes_ropsin
MZ277588_Batillipes_ropsin_1
AFM43710_onychopsin_Phalloceph
AFM43712_onychopsin_Ooperipatu
CCP46946_onychopsin_Euperipato
AFM75824_onychopsin_Euperipato
AFM43711_onychopsin_Eoperipatu
AFM75825_onychopsin_Epiperipat
86
98
Priapulus5112684_1466_10668
MeioTranscriptc76562_g1_i1_g_5
100
EFX83619_arthropsin3_Daphnia_p
EFX83617_arthropsin1_Daphnia_p
EFX83830_arthropsin4_Daphnia_p
EFX83831_arthropsin5_Daphnia_p
EFX83618_arthropsin2_Daphnia_p
EFX84032_arthropsin8_Daphnia_p
CCP46951_arthropsin_Cupiennius
CCP46948_arthropsin_Euperipato
NP_150598_melanopsin_isoform_1
NP_038915_melanopsin_isoform_1
AAL82577_melanopsin_Danio_reri
AAX73255_mammalian_like_melano
AAM95160_melanopsin2_Gadus_mor
AAO20043_melanopsin1_Gadus_mor
AAK59988_putative_photopigment
MZ277607_Paramacrobiotus_arthropsin_3
MZ277609_Paramacrobiotus_arthropsin_3
MZ277610_Macrobiotus_arthropsin
MZ277606_Paramacrobiotus_arthropsin_1
MZ277608_Paramacrobiotus_arthropsin_2
MZ277614_Ramazzottius_arthropsin_3_g6245
MZ277613_Ramazzottius_arthropsin_2_g6218
MZ277612_Ramazzottius_arthropsin_1_g4634
GFGZ01033392_1_TSA_Milnesium_tardigradum_comp16191_c0_seq1
MZ277611_Dactylobiotus_arthropsin
Lotgi1_156440_Lottia_gigantea_
EKC18167_Rhodopsin_GQcoupled_
Lotgi1_154846_Lottia_gigantea_
AGL94565_r_opsin4_Platynereis_
ELU16813_Capca1_124377_Capitel
72
100
AAR18073_rhodopsin_Doryteuthis
CAA40108_rhodopsine_Loligo_for
CAA88923_opsin_Alloteuthis_sub
ACB05673_opsin_Euprymna_scolop
CAA49906_rhodopsin_Todarodes_p
CAA30644_rhodopsin_Enteroctopu
BAA22217_Gq_rhodopsin_Mizuhope
EKC23688_Rhodopsin_GQ_coupled_
Helro1_129809_new_Helobdella_r
Helro1_84106_new_Helobdella_ro
Helro1_85596_new_Helobdella_ro
Helro1_132379_new_Helobdella_r
AGL94564_r_opsin3_Platynereis_
ELU01272_Capca1_119596_Capitel
AAF73286_RHO_G_protein_coupled
CAD13146_opsin_Dugesia_japonic
100
AFZ78086_1_acropsin3_acropora_
aug_v2a_10282_t1_Acropora_digi
100
NV_CN151Suga08_124
100
NV_CN146Suga08_124
