# Supplementary material for: An Ocean of Opsins
Source: Genome Biol Evol. 2025 Nov 4;17(11):evaf189. doi: 10.1093/gbe/evaf189 (PMC12584886; doi:10.1093/gbe/evaf189)
Supplement: evaf189_Supplementary_Data [file Figure_S4.pdf › Figure_S4.pdf]

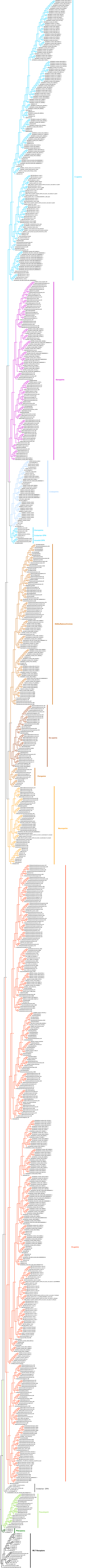

C-opsins
Xenopsins
Cnidopsins
RGRs/Retinochromes
Go-opsins
Peropsins
Neuropsins
R-opsins
Cnidarian OPN
Pseudopsin
Placopsins
MLT Receptors
